# Five new limonoids isolated from *Walsura robusta*

**DOI:** 10.1007/s13659-023-00371-6

**Published:** 2023-02-23

**Authors:** Li Hou, Cui-Xuan Mei, Chun-Mao Yuan, Gui-Hua Tang, Duo-Zhi Chen, Qing Zhao, Hong-Ping He, Ming-Ming Cao, Xiao-Jiang Hao

**Affiliations:** 1 State Key Laboratory of Photochemistry and Plant Resources in West China, and Yunnan Key Laboratory of Natural Medicinal Chemistry, Kunming Institute of Botany, Chinese Academy of Sciences, Kunming, 650201 China; 2 School of Ethnic Medicine, and School of Chinese Materia Medical, Yunnan University of Chinese Medicine, Kunming, 650500 China; 3 University of Chinese Academy of Sciences, Beijing, 100049 China; 4 Bureau of Commerce and Market Supervision of Management Committee of Hunan Xiangjiang New Area, Changsha, 410205 China; 5 The Key Laboratory of Chemistry for Natural Products of Guizhou Province and Chinese Academy of Sciences, Guiyang, 550014 China; 6 Research Unit of Chemical Biology of Natural Anti-Virus Products, Chinese Academy of Medical Sciences, Beijing, 100730 China; 7 Yunnan Characteristic Plant Extraction Laboratory, Kunming, 650106 China

**Keywords:** Limonoids, Tetranortriterpenoids, *Walsura robusta*, Cytotoxicity

## Abstract

Five new toosendanin limonoids with highly oxidative furan ring walsurobustones A-D (**1–4**), and one new furan ring degraded limonoid walsurobustone E (**5**) together with one known compound toonapubesic acid B (**6**) were isolated from the leaves of *Walsura robusta.* Their structures were elucidated by NMR and MS data. Especially, the absolute configuration of toonapubesic acid B (**6**) was confirmed by X-ray diffraction study. Compounds **1–6** exhibited good cytotoxicity against the cancer cell lines HL-60, SMMC**-**7721, A**-**549, MCF**-**7, and SW480.

**Supplementary Information:**

The online version contains supplementary material available at 10.1007/s13659-023-00371-6.

## Introduction

Limonoids, well known as tetranortriterpenoids, were widely found in the families of Meliaceae and Rutaceae, which have attracted great interests from the chemical and biological research communities [1, 2]. The genus *Walsura* (Meliaceae), comprising about 16 species, distributes in subtropical regions such as Southern China, India, and Indonesia [3]. In previous literature, kinds of natural products such as triterpenoids, phenols, and steroids have been identified in this genus. Some triterpenoids and phenols exhibited cell protection, antioxidation and antimalarial activities [4–14]. However, the chemical constituents from *Walsura robusta* and their activities study are few. We reported herein the isolation, characterization as well as cytotoxic activity of these highly oxidized limonoids and a plausible biosynthesis pathway of **5** and **6**.

## Results and discussion

The MeOH extract of *Walsura yunnanensis* was filtered and concentrated in *vacuo* to afford a residue, which was then defatted by petroleum ether and extracted with EtOAc. The EtOAc fraction was subjected to column chromatography on silica gel, ODS, HPLC, and Sephadex LH-20 to yield compounds **1–6** (detailed procedures see Extraction and Isolation part).

Walsurobustone A (**1**) was isolated as a white amorphous powder. Its molecular formula was determined to be C_28_H_38_O_9_ by the [M + Na]^+^ ion peak at *m/z* 541.2418 (calcd 541.2413 for C_28_H_38_O_9_Na) in the HRESIMS, with 16 mass units less than that of the known compound yunnanol A, isolated from the congener plant *W. yunnanensis* [15]. The ^13^C NMR data (Table 2) of **1** showed high similarity to those reported for yunnanol A, with the main difference is the absence of signals for the hydroxyl at C-11 (*δ*_H_ 1.81, 1H, m and 1.56, 1H, m; *δ*_C_ 19.2) in **1**, while *δ*_H_ 5.03 (1H, m) and *δ*_C_ 67.0 in yunnanol A. The proton at H-22 (*δ*_H_ 3.79), coupled with the proton at H-23 (*δ*_H_ 4.77) in the COSY, indicated the two hemiacetal carbons at C-21 (*δ*_C_ 109.5) and C-23 (*δ*_C_ 110.4). A combined analysis of HSQC and HMBC data showing 21-OCH_3_ (*δ*_H_ 3.22, 3H, s), 22-OH (*δ*_H_, 5.33, s) and 23-OCH_3_ (*δ*_H_ 3.30, 3H, s) attached to C-21 (*δ*_C_ 109.5), C-22 (*δ*_C_ 77.5) and C-23 (*δ*_C_ 110.5) respectively revealed identical substituted furan ring when compared to yunnanol A. The HMBC correlations networks of 20-OH (*δ*_H_ 4.35, s) to C-20 (*δ*_C_ 80.4), C-21 (*δ*_C_ 109.5), C-17 (*δ*_C_ 46.8), and C-22 (*δ*_C_ 77.5) confirmed the furan ring attached at C-17. According to the above information, the planar structure of **1** was elucidated as shown.

**Fig. 1:**
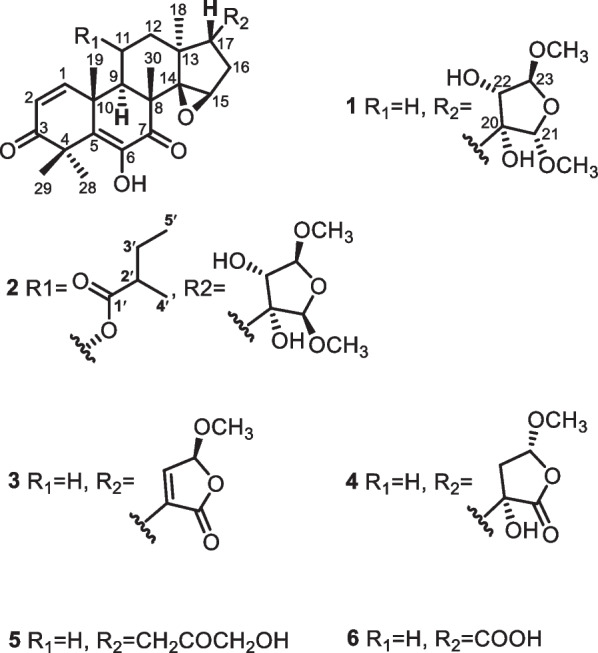
The chemical structures of **1–6**

The absolute stereochemistry of the C-17 was inferred by biosynthetic path as **1** was the homolog with compound **6** whose absolute configuration was determined by X-ray diffraction study. The relative stereochemistry of the tetrahydrofuran ring of **1** was achieved with the aid of ROESY experiment, in which, the correlations of H-21/OMe-23, and H-21/H-22 suggested these protons and the methoxy were homolateral of the tetrahydrofuran ring. The ROESY cross-peaks of H-23/OH-20 indicated that they are on the other face of the tetrahydrofuran ring. The structure of **1** was elucidated as shown in Fig. 1.

Walsurobustone B (**2**), a white amorphous powder, showed a molecular ion peak at *m/z* 641.2938 [M + Na]^+^ in the HRESIMS (calcd for C_33_H_46_O_11_Na 641.2937), consistent with the molecular formula C_33_H_46_O_11_, which revealed that **2** possessed five more carbon and two more oxygen than compound **1**. The difference part was identified as a 2-methylbutyric ester moiety by analysis of ^1^H and ^13^C NMR data (Tables 1 and 2). The correlations of H_3_-5′/H_2_-3′; H_2_-3′/ H-2′; H_3_-4′/H-2′ in ^1^H–^1^H COSY spectra confirmed the presence of 2-methylbutanoate moiety. The location of 2-methylbutyric ester moiety at C-11 was proved by COSY correlation of H-11 (*δ*_H_ 5.16, m)/ H-9 (*δ*_H_ 3.06, d, 11.5 Hz), as well as HMBC correlation from H-11 to C-1′ (*δ*_C_ 174.9). The relative configuration of **2** was established by NOESY experiment. The NOESY correlations H-17/H-21, H-17/ H-22, OH-22/H-23, and H-23/OH-20 indicated the former three protons were on the same side of the tetrahydrofuran ring, while the latter three protons were on another side. The strong cross-peaks of H-11/Me-19 and H-11/Me-30 demonstrated that H-11 was *β*-configuration. Accordingly, the structure of **2** was assigned as depicted in Fig. 1.

**Table 1:** ^1^H NMR spectroscopic data for **1**–**6**

Position	**1**^a^	**2**^a^	**3**^b^	**4**^b^	**5**^b^	**6**^b^
1	7.13, d (10.0)	7.51, d (10.0)	6.89, d (10.0)	7.13, d (10.0)	6.90, d (10.0)	6.91, d (10.0)
2	6.06, d (10.0)	6.10, d (10.0)	6.11, d (10.0)	6.08, d (10.0)	6.11, d (10.0)	6.11, d (10.0)
9	2.59, d (12.0)	3.06, d (11.5)	2.62, m	2.59, d (12.0)	2.63, m	2.60, d (10.6)
11	1.81, m 1.56, m	5.16, m	1.84, m	1.85, m 1.58, m	1.81, m	1.83, m
12	1.74, m 1.89, m	2.19, dd (13.4, 6.8) 1.71, m	2.39, m 1.67, m	1.73, m 1.66, m	1.85, m 1.54, m	2.15, m 1.83, m
15	3.56, s	3.64, s	3.77, s	3.62, s	3.69, s	3.73, s
16	1.94, m 1.76, m	1.94, m 1.77, m	2.25, dd (13.2, 6.0) 1.97, dd (13.6, 11.2)	1.91, m 1.82, m	2.23, t (6.4) 1.46, m	2.25, dd (14.1, 6.9) 2.11, m
17	1.77, m	1.79, m	2.67, m	1.82, m	2.06, m	2.54, dd (10.1, 7.1)
18	1.04, s	1.09, s	0.80, s	0.97, s	0.88, s	0.99, s
19	1.14, s	1.18, s	1.28, s	1.14, s	1.27, s	1.27, s
20					2.36, dd (10.0, 5.5) 2.27, t (10.0)	
21	4.48, s	4.49, s				
22	3.79, dd (7.2, 4.0)	3.72, dd (8.0, 4.4)	6.74, m	2.32, d (17.0) 2.65, d (17.0)		
23	4.77, d (4.0)	4.77, d (4.4)	5.72, d (1.7)	4.96, s	4.23, s	
28	1.45, s	1.45, s	1.57, s	1.45, s	1.48, s	1.56, s
29	1.37, s	1.37, s	1.48, s	1.37, s	1.57, s	1.48, s
30	0.93, s	1.00, s	1.10, s	0.94, s	1.07, s	1.08, s
21-OCH_3_	3.22, s	3.23, s				
23-OCH_3_	3.30, s	3.30, s	3.55, s	3.40, s		
6-OH	8.35, s	8.55, s	6.41, s	8.38, s	6.44, s	
20-OH	4.35, s	4.62, d (3.8)		5.29, s		
22-OH	5.33, d (8.0)	5.43, d (8.0)				
2′		2.39, m				
3′		1.60, m 1.44, m				
4′		1.09, d (1.5)				
5′		0.83, t (7.4)				

^a^Measured in DMSO, ^b^measured in CDCl_3_. Chemical shifts (*δ*) are in ppm from TMS

**Table 2:** ^13^C NMR spectroscopic data for **1**–**6**

Position	**1**^a^	**2**^a^	**3**^b^	**4**^b^	**5**^b^	**6**^b^
1	154.0	155.9	152.5	154.0	152.5	152.4
2	126.2	125.6	127.5	126.3	127.4	127.5
3	203.3	203.3	203.7	203.4	203.8	203.8
4	47.9	48.5	48.7	47.9	48.7	48.7
5	132.7	131.9	134.3	132.8	134.0	134.2
6	142.1	141.8	141.2	142.2	141.3	141.2
7	197.2	195.8	198.1	197.1	198.0	197.8
8	46.7	46.2	46.9	46.7	46.8	46.7
9	42.3	44.3	43.3	42.1	43.5	43.2
10	39.6	40.1	40.4	39.5	40.4	40.4
11	19.2	69.3	19.6	19.2	19.5	19.5
12	35.0	43.1	35.2	35.5	34.9	35.3
13	41.6	40.9	42.2	41.6	41.4	42.5
14	69.1	68.7	69.9	68.8	69.4	69.1
15	54.0	55.2	54.8	54.3	55.1	55.0
16	28.2	27.8	31.9	27.4	33.5	29.6
17	46.8	46.9	42.3	46.1	40.9	51.0
18	22.8	22.5	23.3	23.1	22.4	22.9
19	23.6	24.2	24.1	23.7	24.0	24.0
20	80.4	80.2	138.1	77.3	37.7	178.8
21	109.5	109.3	171.0	175.6		
22	77.5	77.6	144.8	38.6	208.9	
23	110.5	110.3	102.4	109.8	68.5	
28	26.5	26.9	26.9	26.5	26.9	26.9
29	20.8	20.7	21.3	20.9	21.3	21.3
30	19.8	20.9	20.3	19.7	20.4	20.3
21-OCH_3_	54.5	54.5				
23-OCH_3_	55.4	55.6	57.1	56.5		
1′		174.9				
2′		41.0				
3′		26.0				
4′		16.6				
5′		11.5				

^a^Measured in DMSO, ^b^measured in CDCl_3_. Chemical shifts (*δ*) are in ppm from TMS

Walsurobustone C (**3**), a white amorphous powder, had the molecular formula of C_27_H_32_O_7_ as established by the HRESIMS at *m/z* 491.2048 [M + Na]^+^ (calcd. 491.2045). The ^1^H and ^13^C NMR data (Tables 1 and 2) suggested that the compound **3** was structurally related to walsunoid B [16] as the NMR data of both the compounds are almost similar. The main difference between **3** and walsunoid B is the absence of signals for the hydroxyl at C-11(*δ*_H_ 1.84, 2H, m; *δ*_C_ 19.6) in **3**, while C-11 (*δ*_H_ 5.05, 1H, d, 6.2 Hz; *δ*_C_ 66.5) in walsunoid B. Particularly, 23-OMe in **3** was assigned to be *α*-oriented by comparing the relevant NMR data with a reported compound walsunoid D [16] (*δ*_H_ 5.72 (1H, m) and *δ*_C_ 102.4 of CH-23 in **3**; δ_H_ 5.73 (1H, brs) and *δ*_C_ 102.5 of CH-23 in walsunoid D). Thus compound **3** was deduced as walsurobustone C (Fig. 1).

Walsurobustone D (**4**) also exhibited a molecular formula ion peak at *m/z* 509.2141 [M + Na]^+^ (calcd for C_27_H_34_O_8_Na 509.2151) in its HRESIMS. The ^1^H and ^13^C NMR data of **4** is similar to that of isowalsuranolide [4] except for additional NMR signals (*δ*_C_ 56.5 and *δ*_H_ 3.40) due to a methoxy group and the hydrated double bond in the E ring. The location of the methoxy group was established by the HMBC correlation of methoxy methyl protons to the hemiacetal carbon signal at C-21 (*δ*_C_ 109.8). Furthermore, the HMBC correlations from *δ*_H_ 5.29 (s, –OH) to C-20 (*δ*_C_ 77.3), C-21 (*δ*_C_ 109.8), C-17 (*δ*_C_ 46.1) and C-22 (*δ*_C_ 38.6) indicated that the hydroxyl group *δ*_H_ 5.29 attached to the characteristic quaternary carbon C-20 (*δ*_C_ 77.3). The relative configuration of the tetrahydrofuran ring of **4** was established by analysis of the ROESY spectrum. ROESY correlation between H-17 and H-23 indicated that they were co-facial and arbitrarily assigned as *β*-oriented. The correlations of Me-18 with OH-20, OH-20 with OMe-23 supported the *α*-orientation of OH-20 and OMe-23. Thus, the structure of **4** has been determined as showed in Fig. 1.

**Fig. 2:**
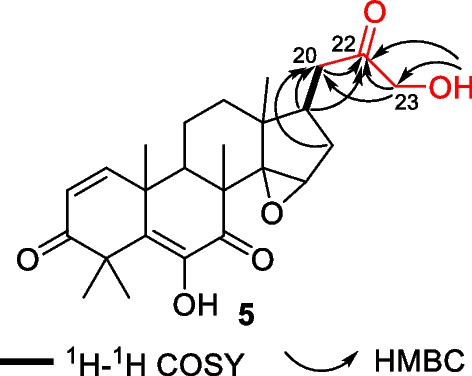
Key ^1^H–^1^H COSY and HMBC (H → C) correlations of **5**

Walsurobustone E (**5**) was isolated as a white amorphous powder, and the molecular formula C_25_H_32_O_6_ was assigned through its HRESIMS, at *m/z* 451.2096 [M + Na]^+^ (calcd for C_25_H_32_O_6_Na, 451.2091). The ^1^H and ^13^C NMR spectra showed signals for five methyls (*δ*_H_ 0.88, 1.07, 1.27, 1.48, and 1.57, each 3H), five methylenes, five methines, four high-field quaternary carbons (*δ*_C_ 48.7, 46.8, 40.4 and 41.4), two double bonds (*δ*_C_ 152.5, 141.3, 127.4, and 134.0) and three carbonyl carbons (*δ*_C_ 208.9, 203.8, and 198.0). These data indicated that **5** has the same core structure as cedrelone [17], except the absence of furan ring signal feature in compound **5**. Based on the HSQC and HMBC data analysis, a hydroxyl acetone moiety was revealed by a hydroxylmethyl group C-23 (*δ*_C_ 68.5, *δ*_H_ 4.23, and hydroxyl *δ*_H_ 5.31) attached to carbonyl C-22 (*δ*_C_ 208.7) (Fig. 2). The carbonyl C-22 linked to C-17 via C-20 was established by the HMBC correlations from H-17 (*δ*_H_ 2.06) to C-20 (*δ*_C_ 37.7) and C-22 (*δ*_C_ 208.7), and H-16a (*δ*_H_ 1.46) to C-20. Accordingly, the structure of **7** was assigned as depicted in Fig. 1.

**Fig. 3:**
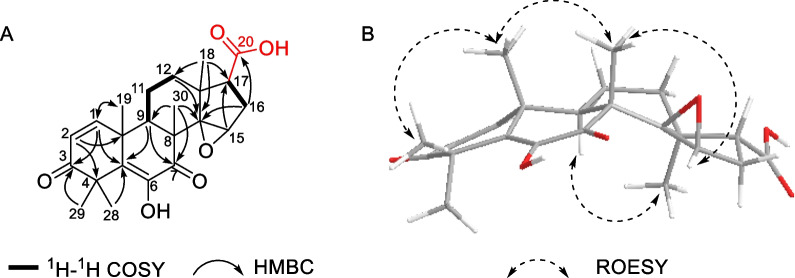
Key ^1^H–^1^H COSY and HMBC (H → C) correlations (A), and selective ROESY correlations (B) of **6**

Toonapubesic acid B [18] (**6**) was obtained as a colorless crystal (recrystallized in MeOH/CH_3_COCH_3_) and the molecular formula C_23_H_28_O_6_ was assigned through its HRESIMS, at *m/z* 423.1777 [M + Na]^+^ (calcd for C_23_H_28_O_6_Na, 423.1783), suggesting 10 degrees of unsaturation. The ^13^C NMR spectrum (Table 2) of **6** were very close to those for **5**, except that the furan ring was oxidized to a carboxylic acid in compound **6**. The inferred structure was further established through analysis of its MS and HMBC data. In the HMBC spectrum, H-17 (*δ*_H_ 2.54) and H-16*β* (*δ*_H_ 2.11) were correlated with the carbon resonance at *δ*_C_ 178.8, which was assigned to the C-20 carboxyl group. According to the above information, the planar structure of **6** was elucidated as shown (Fig. 3A).

**Fig. 4:**
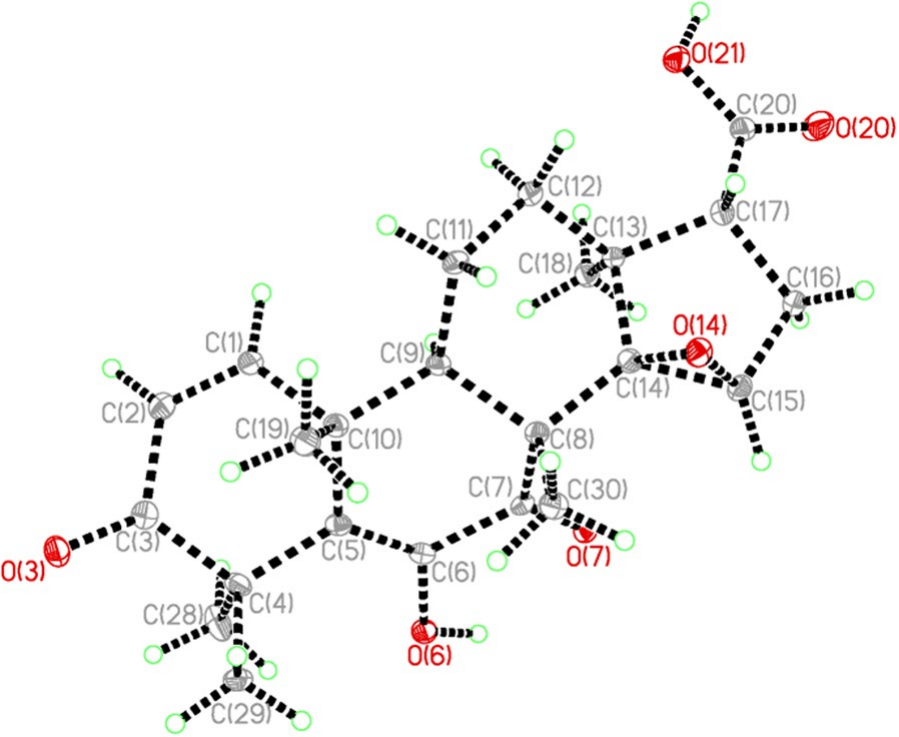
Single-crystal X-ray structure of **6**

The relative configuration of **6** was determined by ROESY spectrum (Fig. 3B). As shown in ROESY data, the correlations of H_3_-18/H-9, H_3_-18/H-16*α*, and H_3_-18/H-11α indicated that they were co-facial, arbitrarily assigned as *α*-orientated. The ROESY correlations of H_3_-30/H_3_-19, H_3_-19/H_3_-29 revealed that H_3_-30, H_3_-19, and H_3_-29 were *β*-oriented, thus H_3_-28 was *α*-orientated. The configuration of five methyls was the same as other reported limonoids [4]. In order to fix the orientation of the epoxide, **6** was recrystallized in methanol: acetone = 1: 1 mixture solvent and been structurally determined by X-ray diffraction analysis (Fig. 4). The X-ray diffraction data were collected by using CuK_α_ radiation and its absolute configuration has been determined as *8R*, *9R*, *10R*, *13S*, *14R*, *15R*, and *17R*. Although the structure of toonapubesic acid B (**6**) was reported in literature [18], while its NMR data was not available. Here we report the X-ray structure of toonapubesic acid B and its NMR data together to facilitate the community.

**Scheme 1:**
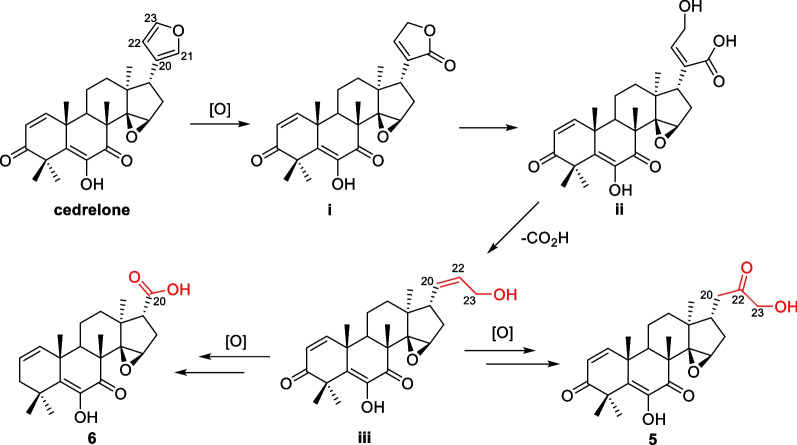
Hypothesis route of **5** and **6**

Hypothetic biosynthesis of *seco*-E ring derivatives **5** and **6** was proposed based on the common structural characteristics of discovered intermediates (Scheme 1). The furan ring of cedrelone was oxidized to form butenolactone, which was hydrolyzed to give free carboxyl and hydroxyl groups subsequently. After decarboxylation and a few steps oxidation, **5** and **6** formed respectively.

**Fig. 5:**
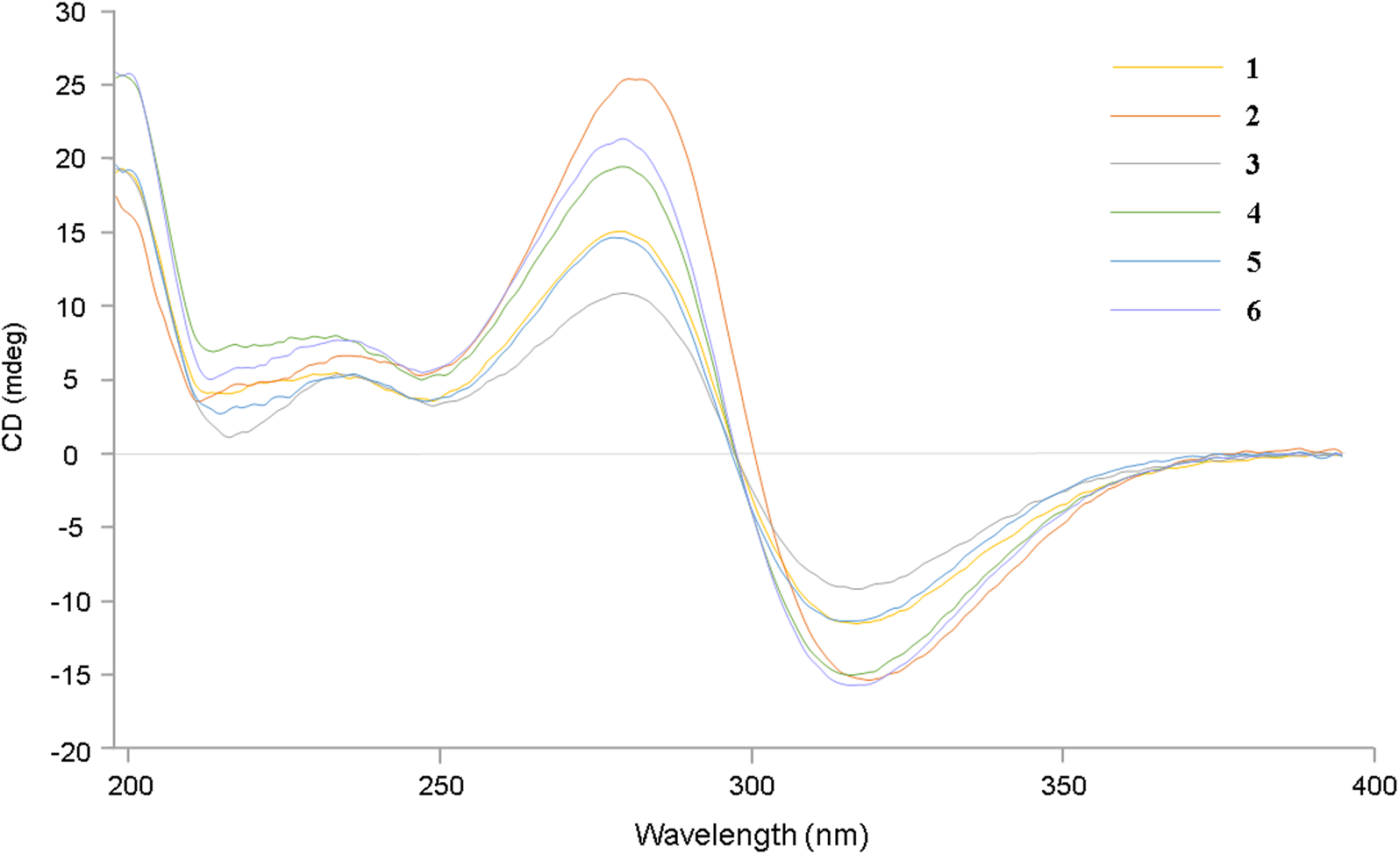
CD spectra for walsurobustones A-E (**1**–**5**) and Toonapubesic acid B (**6**) (in MeOH)

The similar patterns of Cotton Effects in the CD spectra of walsurobustones A-E (**1**–**5**) and toonapubesic acid B (**6**) (Fig. 5) indicated that the former’s chiral centers in rings A, B, C and D have absolute configurations identical to the latter.

The cytotoxicity of **1**–**6** against the cancer cell lines (HL-60, SMMC**-**7721, A**-**549, MCF**-**7, and SW480) were evaluated by using the MTT method with taxol and cisplatin as the positive control. While compound **6** only showed moderate effects for all cell lines, other five compounds all showed obvious activity, especially to SMMC-7721, A-549 and MCF-7 (Table 3).

**Table 3:** Cytotoxicity Data for Compounds **1**–**6** against the cancer cell lines^a^

Compounds	HL-60	SMMC-7721	A-549	MCF-7	SW480
Walsurobustone A (**1**)	16.10	3.51	5.86	3.59	4.70
Walsurobustone B (**2**)	5.12	6.10	4.56	2.76	6.95
Walsurobustone C (**3**)	2.58	0.44	2.11	2.09	3.78
Walsurobustone D (**4**)	17.54	5.81	5.68	9.67	13.10
Walsurobustone E (**5**)	5.62	4.09	3.63	5.70	11.48
Toonapubesic acid B (**6**)	20.31	10.24	13.41	13.81	15.00
Cisplatin	1.81	8.86	11.68	15.92	16.65
Taxol	< 0.008	< 0.008	< 0.008	< 0.008	< 0.008

^a^Results are expressed as IC_50_ values in μM. HL-60 leukemia cancer; SMMC-7721 liver cancer; A-549 lung cancer; MCF-7 breast cancer; SW480 colon cancer. All the compounds exhibited potential cytotoxicity

## Experimental section

### General experimental procedure

Optical rotations were measured with a Horiba SEPA-300 polarimeter. UV spectra were detected on a Shimadzu UV-2401 spectrophotometer. IR spectra were determined on a Tenor 27 spectrophotometer with KBr pellets, whereas CD spectra were recorded with an Applied Photophysics Chirascan spectrometer. ESIMS and HRESIMS were measured on a Finnigan MAT 90 instrument and VG Auto Spec-3000 spectrometer, respectively. 1D and 2D NMR spectra were recorded on Bruker AM-400, Bruker DRX-500 and Bruker Avance III 600 spectrometers with TMS as internal standard. Semipreparative HPLC was performed on an Agilent 1100 liquid chromatographer with a Zorbax SB-C18 (9.4 mm 25 cm) column. Column chromatography was performed with silica gel (300–400 mesh, Qingdao Marine Chemical, Inc., Qingdao, People’s Republic of China), and MCI gel (75–150 μM, Mitsubishi Chemical Corporation, Tokyo, Japan). Fractions were monitored by TLC, and spots were visualized by heating silica gel plates sprayed with 10% H_2_SO_4_ in EtOH.

### Plant material

The leaves of* W. robusta* collected in Hainan Province, People’s Republic of China in December 2010. The plant was authenticated by Dr. Guangwan Hu, Kunming Institute of Botany, Chinese Academy of Sciences. A voucher specimen (No. H20101202) was deposited in the State Key Laboratory of Photochemistry and Plant Resources in West China, Kunming Institute of Botany, Chinese Academy of Sciences.

### Extraction and isolation

The dried and powdered leaves (12 kg) of* W. robusta* were extracted with MeOH three times under reflux, and the solvent was evaporated in *vacuo.* The residue was partitioned in H_2_O and extracted successively with petroleum ether and EtOAc. The EtOAc fraction (200 g) was separated by silica gel column chromatography (CC) eluted with a gradient of petroleum ether/Me_2_CO (50:1 to 1:1) and CHCl_3_/MeOH in a gradient (15:1 to 3:1), eight fractions (Fr. A-H) were obtained according to TLC monitor. Fr. C (17 g) was subjected to MCI-gel column (MeOH/H_2_O, 6:4 to 9:1) to give sixteen sub-fractions (C1–C16). C7 (4.1 g) was then chromatographed on a column of reversed-phase C18 silica gel eluted with MeOH/H_2_O (5:5 to 9:1) to get eight parts (C7a–C7h). C7h was purified by CC over silica gel and then applied to a Sephadex LH-20 column using a solvent system acetone to provide **1** (5 mg), **3** (5 mg), **4** (11 mg), **5** (15 mg). C8 was further purified by preparative RP-8 HPLC using a solvent system 60% aqueous MeOH to provide compounds **2** (11 mg). Fr. D (60 g) was subjected to MCI-gel column (MeOH/H_2_O, 4:6 to 9:1) to give twenty fractions (D1–D20), D15 (1 g) was subjected to silica gel column chromatography eluting sequentially with petroleum ether/EtOAc to afford 11 fractions, the second portion was purified by Sephadex LH-20 column to obtain **6** (11 mg).

Walsurobustone A (**1**), a white amorphous powder; [*α*] − 30.6275 (*c* 0.17, CHCl_3_); UV (CHCl_3_) λ_max_ (logε) 283 (3.10) nm; ^1^H NMR (DMSO) and ^13^C NMR (DMSO) (see Tables 1 and 2). IR ν_max_ cm^−1^: 3430, 1630 cm^−1^; HRESIMS at *m/z* 509.2141 [M + Na]^+^ (calcd for C_27_H_34_O_8_Na, 509.2151).

Walsurobustone B (**2**), a white amorphous powder, [*α*] − 20.25(*c* 0.16, CHCl_3_); UV (CHCl_3_) λ_max_ (logε) 283(3.10) nm; ^1^H NMR (DMSO) and ^13^C NMR (DMSO) (see Tables 1 and 2). IR ν_max_ cm^−1^: 3426, 1682, 1630, 1030, 998 cm^−1^; HRESIMS at *m/z* 641.2938 [M + Na]^+^ in the HRESIMS (calcd for C_33_H_46_O_11_Na, 641.2937).

Walsurobustone C (**3**), a white amorphous powder; [*α*] − 21.1905 (*c* 0.14, CHCl_3_); UV (CHCl_3_) λ_max_ (logε) 281 (3.30) nm; ^1^H NMR (CDCl_3_) and ^13^C NMR (CDCl_3_) (see Tables 1 and 2). IR ν_max_ cm^−1^: 3406, 2957, 2923, 1764, 1676, 1034 cm^−1^; HRESIMS at *m/z* 491.2048 [M + Na]^+^ (calcd for C_27_H_32_O_7_Na, 491.2045).

Walsurobustone D (**4**), a white amorphous powder; [*α*] − 68.6111 (*c* 0.12, CHCl_3_); UV (CHCl_3_) λ_max_ (logε) 283 (3.12) nm; ^1^H NMR (CDCl_3_) and ^13^C NMR (CDCl_3_) (see Tables 1 and 2). IR ν_max_ cm^−1^: 3431, 1630 cm^−1^; HRESIMS at *m/z* 509.2141 [M + Na]^+^ (calcd for C_27_H_34_O_8_Na, 509.2151).

Walsurobustone E (**5**), a white amorphous powder; [*α*] − 54.5641 (*c* 0.14, CHCl_3_); UV (CHCl_3_) λ_max_ (logε) 281 (3.28) nm; ^1^H NMR (CDCl_3_) and ^13^C NMR (CDCl_3_) (see Tables 1 and 2). IR ν_max_ cm^−1^: 3482, 3406, 2924, 2854, 1718, 1680, 1356, 1249, 1031 cm^−1^; HRESIMS *m/z* 451.2103 [M + Na] ^+^ (calcd for C_23_H_28_O_6_Na, 451.2096).

Toonapubesic acid B (**6**), a colorless crystal, deposited to CCDC with No.2235264; [*α*] − 79.4872 (*c* 0.13, CHCl_3_); UV(CHCl_3_) λ_max_ (logε) 280 (3.26) nm; ^1^H NMR (CDCl_3_) and ^13^C NMR (CDCl_3_) (see Tables 1 and 2). IR ν_max_ cm^−1^: 3418, 2926, 1713, 1686, 1240, 1031 cm^−1^; HRESIMS *m/z* 423.1777 [M + Na]^+^ (calcd for C_23_H_28_O_6_Na, 423.1783).

## Supplementary Information

Supplementary file 1
