# Supplementary material for: Five new limonoids isolated from Walsura robusta
Source: Nat Prod Bioprospect. 2023 Feb 23;13(1):7. doi: 10.1007/s13659-023-00371-6 (PMC9947198; doi:10.1007/s13659-023-00371-6)
Supplement: Supplementary file 1 [file 13659_2023_371_MOESM1_ESM.doc]

Supporting Imformation

Five new limonoids isolated from *Walsura robusta*

Li Hou^1, 2^, Cui-Xuan Mei^1^, Gui-Hua Tang^1^, Yu Zhang^1^, Yan Li^1^, Chun-Mao Yuan^1,3^, Qing Zhao^2^, Hong-Ping He^1,2^, Mingming Cao^1,*^, and Xiao-Jiang Hao^1,3,4*^

1. State Key Laboratory of Photochemistry and Plant Resources in West China, Kunming Institute of Botany, Chinese Academy of Sciences, Kunming Yunnan 650201, China;

2. School of Ethnic Medicine and School of Chinese Materia Medica, Yunnan University of Chinese Medicine, Kunming Yunnan 650500, China;

3. The Key Laboratory of Chemistry for Natural Products of Guizhou Province and Chinese Academy of Sciences, Guiyang 550014, China;

4. Research Unit of Chemical Biology of Natural Anti-Virus Products, Chinese Academy of Medical Sciences, Beijing, 100730, China.

**CONTENT**

[**Figure S1. ^1^H NMR spectrum (400 MHz) of walsurobustone A (**1**) in (CD_3_)_2_SO** 4](#__RefHeading___Toc123649702)

[**Figure S2. ^13^C NMR spectrum (100 MHz) of walsurobustone A (**1**) in (CD_3_)_2_SO** 4](#__RefHeading___Toc123649703)

[**Figure S3. ^1^H-^1^H COSY spectrum (500 MHz) of walsurobustone A (**1**) in (CD_3_)_2_SO** 4](#__RefHeading___Toc123649704)

[**Figure S4. HSQC spectrum (500 MHz) of walsurobustone A (**1**) in (CD_3_)_2_SO** 5](#__RefHeading___Toc123649705)

[**Figure S5. HMBC spectrum (500 MHz) of walsurobustone A (**1**) in (CD_3_)_2_SO** 5](#__RefHeading___Toc123649706)

[**Figure S6. ROESY spectrum (500 MHz) of walsurobustone A (**1**) in (CD_3_)_2_SO** 6](#__RefHeading___Toc123649707)

[**Figure S7. ESIMS spectrum of walsurobustone A (**1**)** 7](#__RefHeading___Toc123649708)

[**Figure S8. HRESIMS spectrum of walsurobustone A (**1**)** 7](#__RefHeading___Toc123649709)

[**Figure S9. IR (KBr disc) spectrum of walsurobustone A (**1**)** 7](#__RefHeading___Toc123649710)

[**Figure S10.^1^H NMR spectrum (600 MHz) of walsurobustone B (**2**) in (CD_3_)_2_SO** 8](#__RefHeading___Toc123649711)

[**Figure S11.^13^CNMR spectrum (150 MHz) of walsurobustone B (**2**) in (CD_3_)_2_SO** 8](#__RefHeading___Toc123649712)

[**Figure S12. ^1^H-^1^H COSY spectrum (600 MHz) of walsurobustone B (**2**) in (CD_3_)_2_SO** 9](#__RefHeading___Toc123649713)

[**Figure S13. HSQC spectrum (600 MHz) of walsurobustone B (**2**) in (CD_3_)_2_SO** 9](#__RefHeading___Toc123649714)

[**Figure S14. HMBC spectrum (600 MHz) of walsurobustone B (**2**) in (CD_3_)_2_SO** 10](#__RefHeading___Toc123649715)

[**Figure S15. ROESY spectrum (600 MHz) of walsurobustone B (**2**) in (CD_3_)_2_SO** 10](#__RefHeading___Toc123649716)

[**Figure S16. ESIMS spectrum of walsurobustone B (**2**)** 11](#__RefHeading___Toc123649717)

[**Figure S17. HRESIMS spectrum of walsurobustone B (**2**)** 11](#__RefHeading___Toc123649718)

[**Figure S18. IR (KBr disc) spectrum of walsurobustone B (**2**)** 12](#__RefHeading___Toc123649719)

[**Figure S19.^1^H NMR spectrum (400 MHz) of walsurobustone C (**3**) in CDCl_3_** 12](#__RefHeading___Toc123649720)

[**Figure S20.^13^CNMR spectrum (100 MHz) of walsurobustone C(**3**) in CDCl_3_** 13](#__RefHeading___Toc123649721)

[**Figure S21. ^1^H-^1^H COSY spectrum (500 MHz) of walsurobustone C (**3**) in CDCl_3_** 13](#__RefHeading___Toc123649722)

[**Figure S22. HSQC spectrum (500 MHz) of walsurobustone C (**3**) in CDCl_3_** 14](#__RefHeading___Toc123649723)

[**Figure S23. HMBC spectrum (500 MHz) of walsurobustone C (**3**) in CDCl_3_** 14](#__RefHeading___Toc123649724)

[**Figure S24. ROESY spectrum (500 MHz) of walsurobustone C (**3**) in CDCl_3_** 15](#__RefHeading___Toc123649725)

[**Figure S25. ESIMS spectrum of walsurobustone C (**3**)** 15](#__RefHeading___Toc123649726)

[**Figure S26. HRESIMS spectrum of walsurobustone C (**3**)** 16](#__RefHeading___Toc123649727)

[**Figure S27. IR (KBr disc) spectrum of walsurobustone C (**3**)** 16](#__RefHeading___Toc123649728)

[**Figure S28. ^1^H NMR spectrum (500 MHz) of walsurobustone D (**4**) in (CD_3_)_2_SO** 17](#__RefHeading___Toc123649729)

[**Figure S29. ^13^C NMR spectrum (100 MHz) of walsurobustone D (**4**) in (CD_3_)_2_SO** 17](#__RefHeading___Toc123649730)

[**Figure S30. ^1^H-^1^H COSY spectrum (500 MHz) of walsurobustone D (**4**) in (CD_3_)_2_SO** 18](#__RefHeading___Toc123649731)

[**Figure S31. HSQC spectrum (500 MHz) of walsurobustone D (**4**) in (CD_3_)_2_SO** 18](#__RefHeading___Toc123649732)

[**Figure S32. HMBC spectrum (500 MHz) of walsurobustone D (**4**) in (CD_3_)_2_SO** 19](#__RefHeading___Toc123649733)

[**Figure S33. ROESY spectrum (500 MHz) of walsurobustone D (**4**) in (CD_3_)_2_SO** 19](#__RefHeading___Toc123649734)

[**Figure S34. ESIMS spectrum of walsurobustone D (**4**)** 20](#__RefHeading___Toc123649735)

[**Figure S35. HRESIMS spectrum of walsurobustone D (**4**)** 20](#__RefHeading___Toc123649736)

[**Figure S36. IR (KBr disc) spectrum of walsurobustone D (**4**)** 21](#__RefHeading___Toc123649737)

[**Figure S37. ^1^H NMR spectrum (400 MHz) of walsurobustone E (**5**) in CDCl_3_** 21](#__RefHeading___Toc123649738)

[**Figure S38. ^13^CNMR spectrum (100 MHz) of walsurobustone E (**5**) in CDCl_3_** 22](#__RefHeading___Toc123649739)

[**Figure S39. ^1^H-^1^H COSY spectrum (500 MHz) of walsurobustone E (**5**) in CDCl_3_** 22](#__RefHeading___Toc123649740)

[**Figure S40. HSQC spectrum (500 MHz) of walsurobustone E (**5**) in CDCl_3_** 23](#__RefHeading___Toc123649741)

[**Figure S41. HMBC spectrum (500 MHz) of walsurobustone E (**5**) in CDCl_3_** 23](#__RefHeading___Toc123649742)

[**Figure S42. ROESY spectrum (500 MHz) of walsurobustone E (**5**) in CDCl_3_** 24](#__RefHeading___Toc123649743)

[**Figure S43. ESIMS spectrum of walsurobustone E (**5**)** 24](#__RefHeading___Toc123649744)

[**Figure S44. HRESIMS spectrum of walsurobustone E (**5**)** 25](#__RefHeading___Toc123649745)

[**Figure S45. IR (KBr disc) spectrum of walsurobustone E (**5**)** 25](#__RefHeading___Toc123649746)

[**Figure S46.^1^H NMR spectrum (400 MHz) of toonapubesic acid B (**6**) in CDCl_3_** 26](#__RefHeading___Toc123649747)

[**Figure S47.^13^CNMR spectrum (100 MHz) of toonapubesic acid B (**6**) in CDCl_3_** 26](#__RefHeading___Toc123649748)

[**Figure S48. ^1^H-^1^H COSY spectrum (500 MHz) of toonapubesic acid B (**6**) in CDCl_3_** 27](#__RefHeading___Toc123649749)

[**Figure S49. HSQC spectrum (500 MHz) of toonapubesic acid B (**6**) in CDCl_3_** 27](#__RefHeading___Toc123649750)

[**Figure S50. HMBC spectrum (500 MHz) of toonapubesic acid B (**6**) in CDCl_3_** 28](#__RefHeading___Toc123649751)

[**Figure S51. ROESY spectrum (500 MHz) of toonapubesic acid B (**6**) in CDCl_3_** 28](#__RefHeading___Toc123649752)

[**Figure S52. ESIMS spectrum of toonapubesic acid B (**6**)** 29](#__RefHeading___Toc123649753)

[**Figure S53. HRESIMS spectrum of toonapubesic acid B (**6**)** 29](#__RefHeading___Toc123649754)

[**Figure S54. IR (KBr disc) spectrum of toonapubesic acid B (**6**)** 30](#__RefHeading___Toc123649755)

[**Figure S55. Single-crystal X-ray structure of toonapubesic acid B (**6**)** 30](#__RefHeading___Toc123649756)


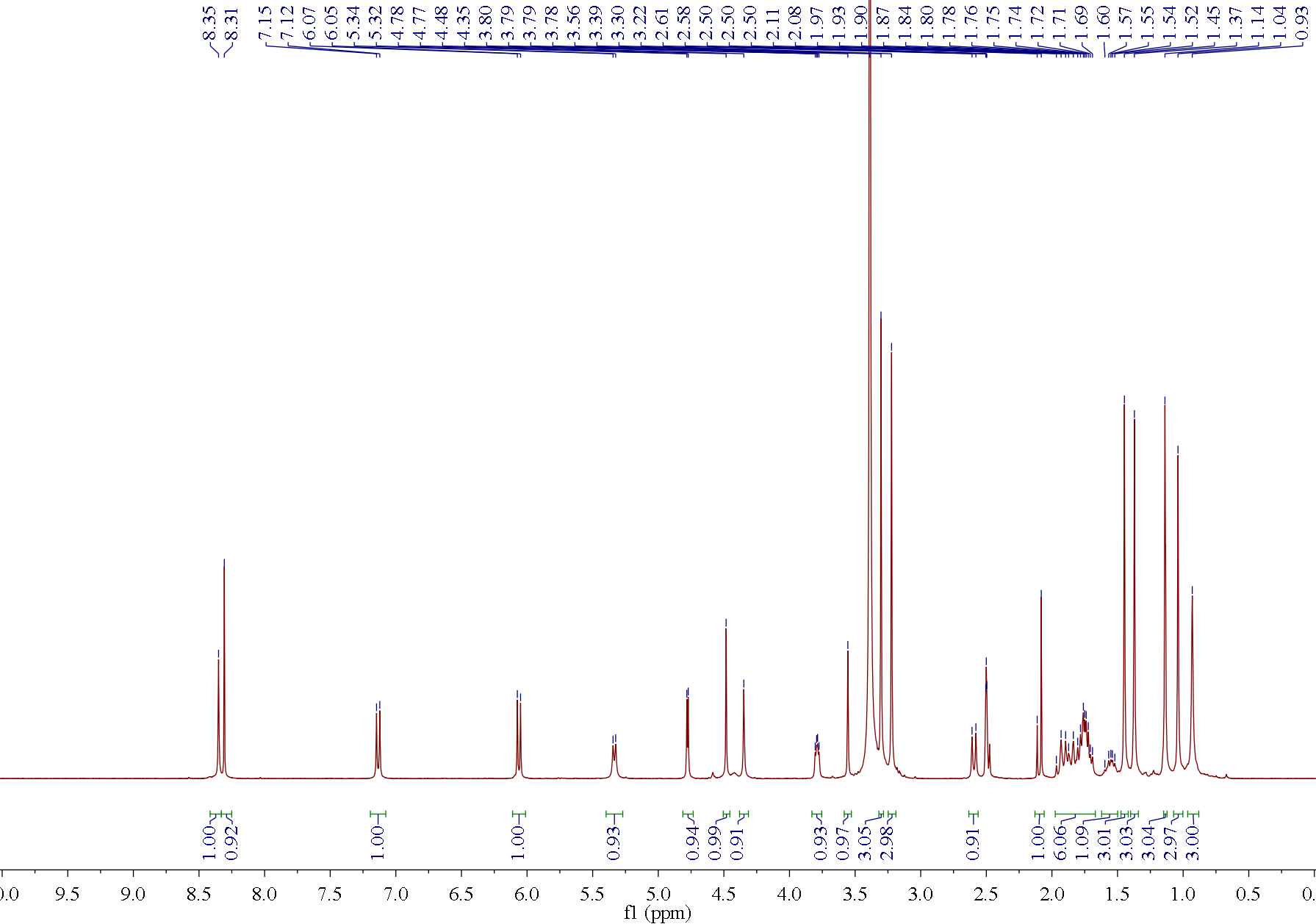
Figure S1. ^1^H NMR spectrum (400 MHz) of walsurobustone A (1) in (CD_3_)_2_SO

Figure S2. ^13^C NMR spectrum (100 MHz) of walsurobustone A (1) in (CD_3_)_2_SO


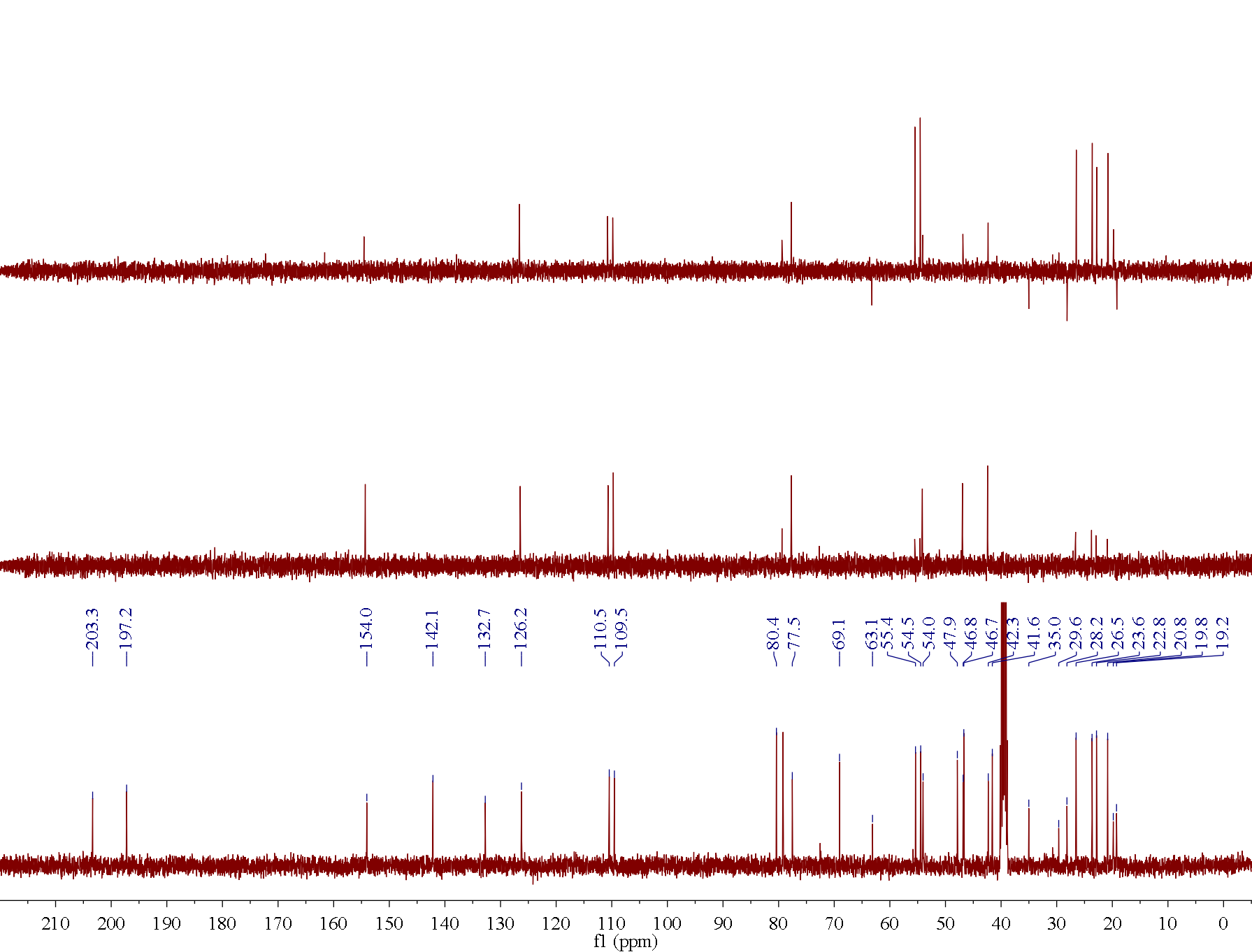


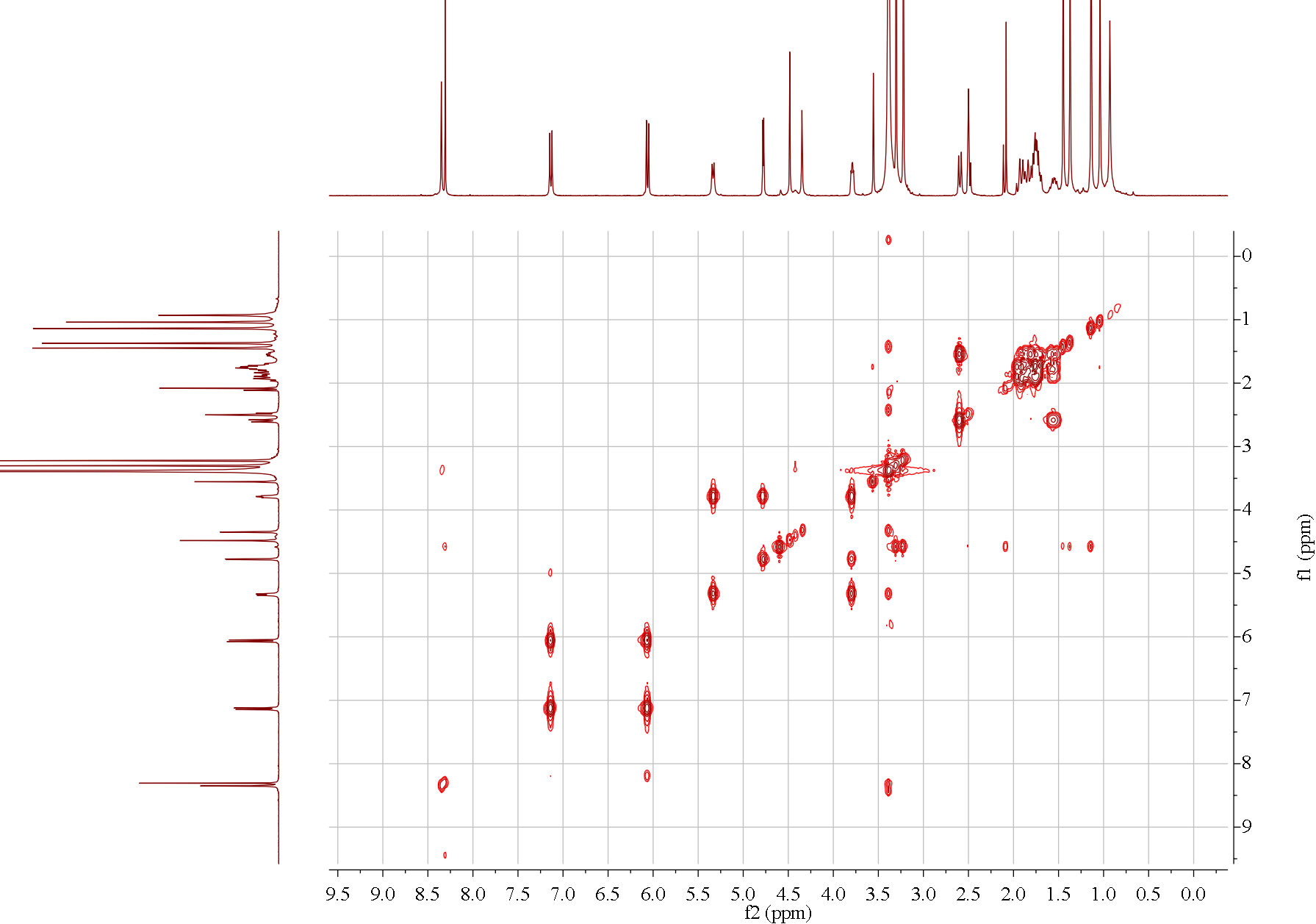
Figure S3. ^1^H-^1^H COSY spectrum (500 MHz) of walsurobustone A (1) in (CD_3_)_2_SO

Figure S4. HSQC spectrum (500 MHz) of walsurobustone A (1) in (CD_3_)_2_SO


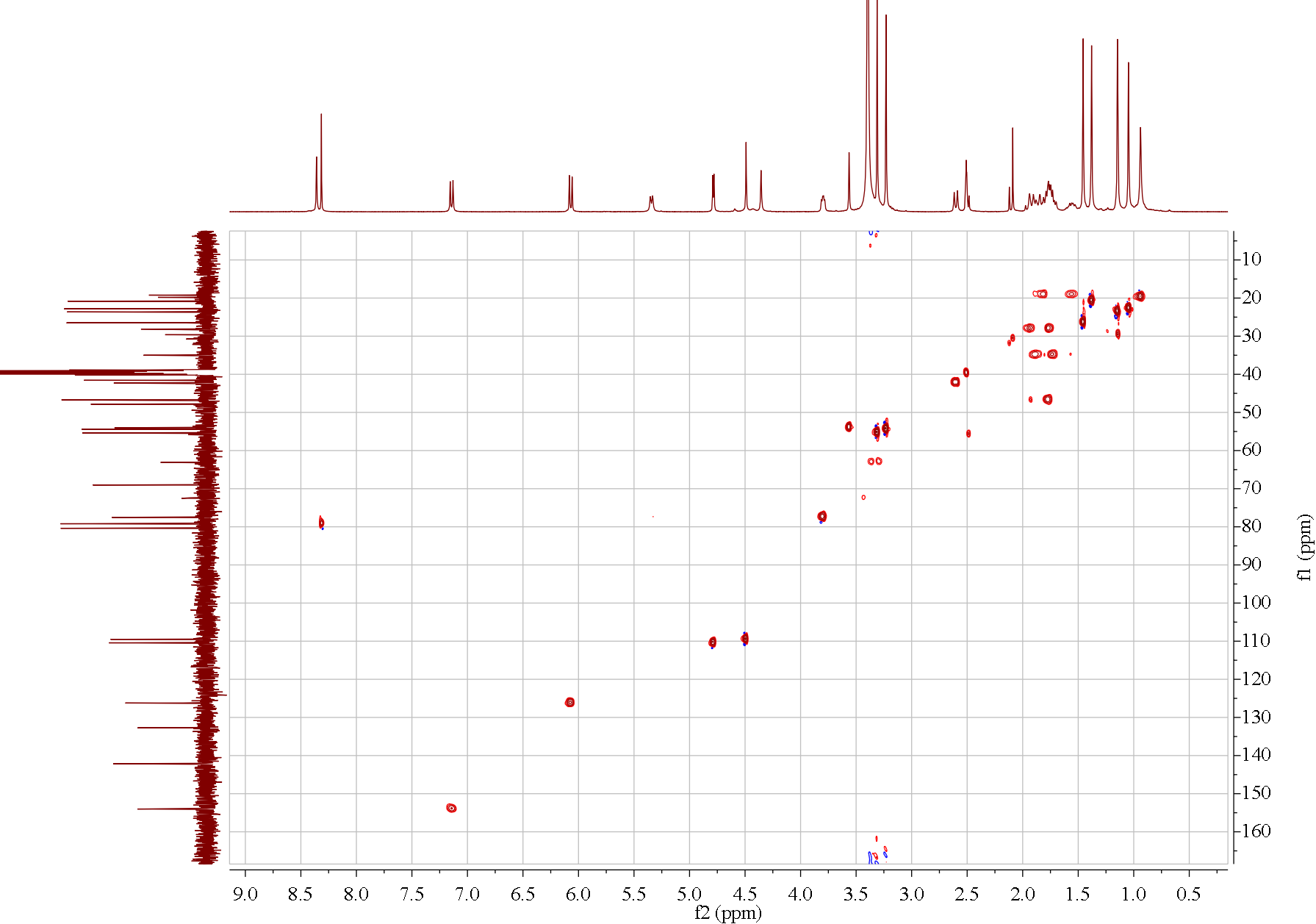


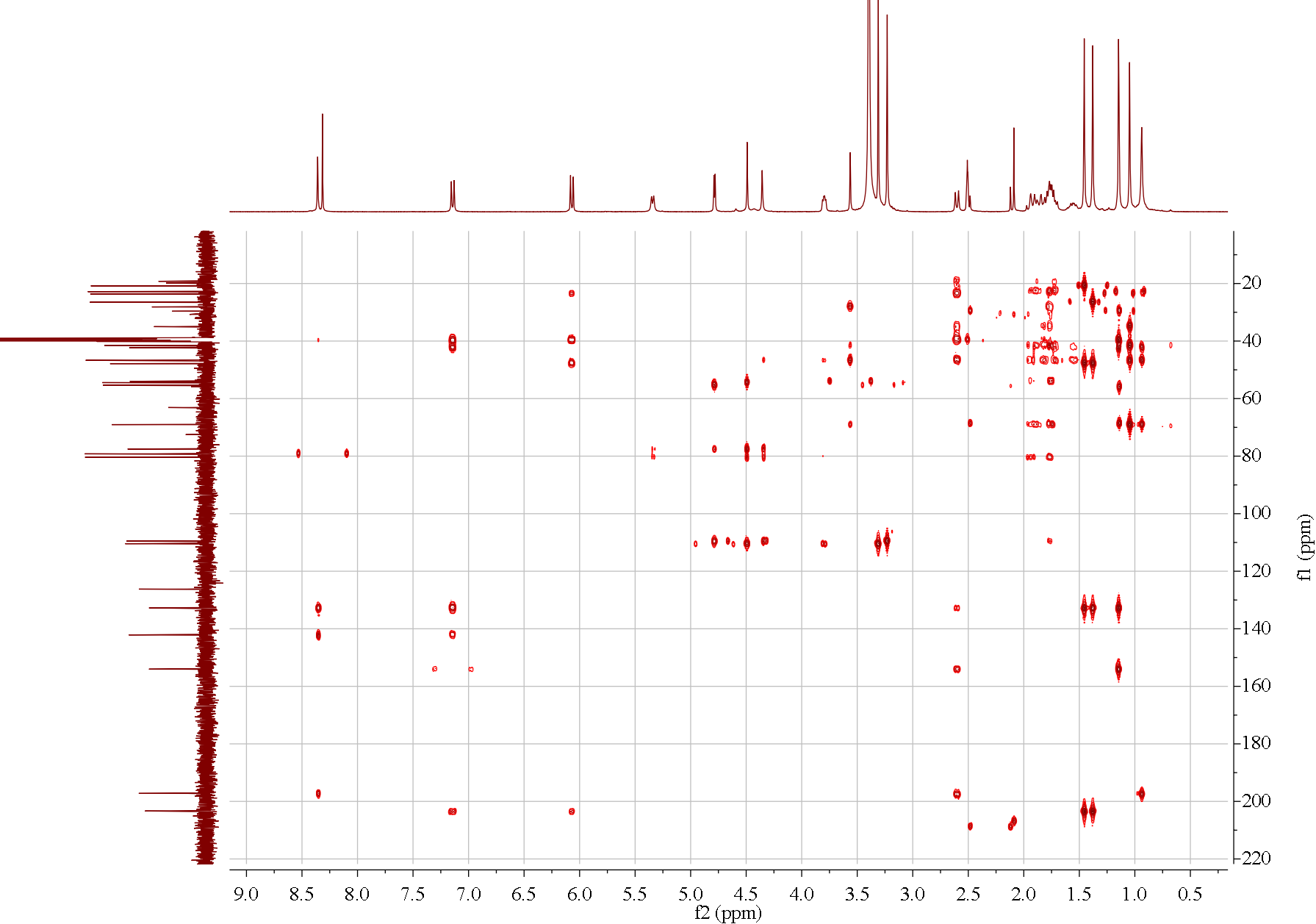
Figure S5. HMBC spectrum (500 MHz) of walsurobustone A (1) in (CD_3_)_2_SO


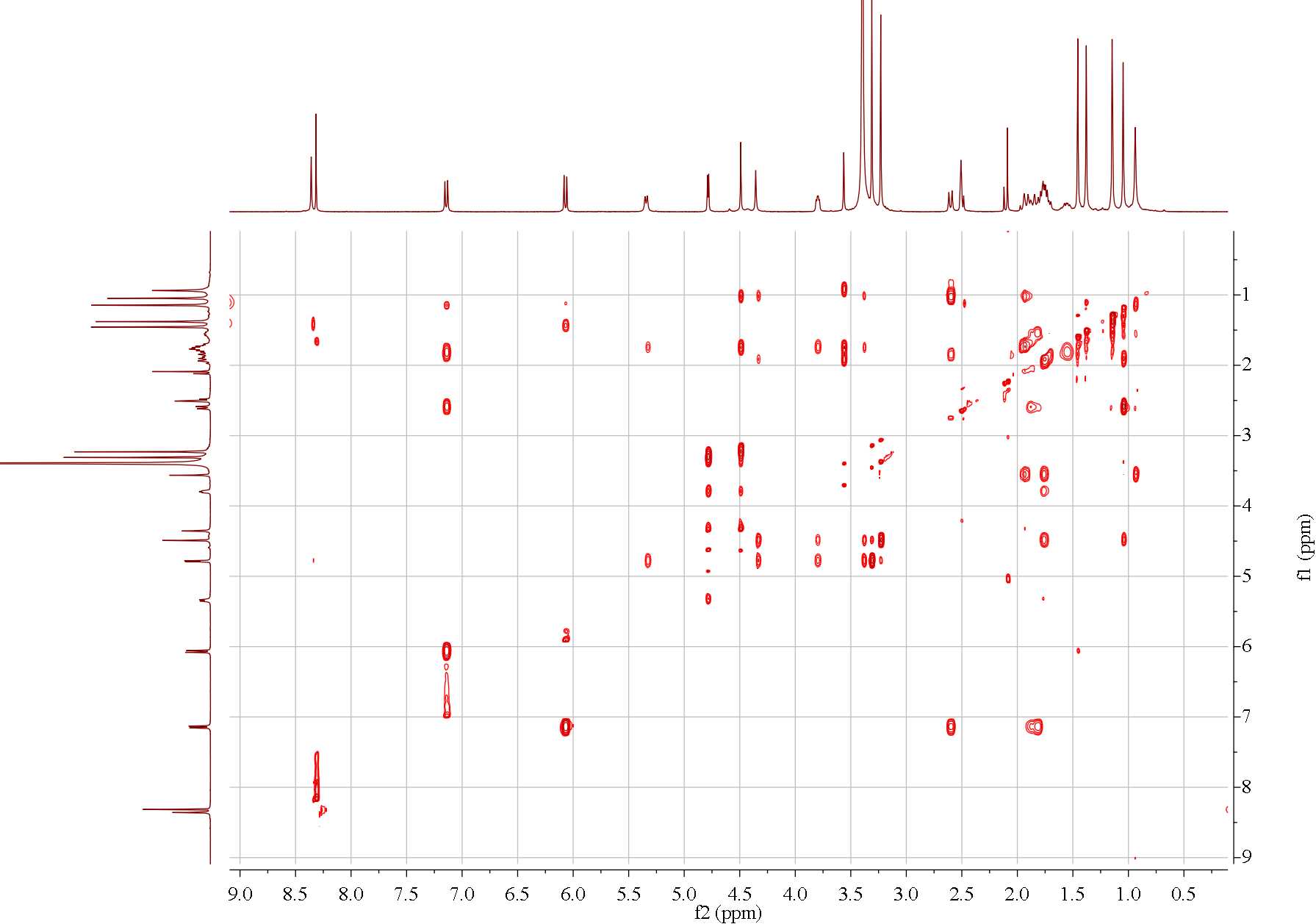
Figure S6. ROESY spectrum (500 MHz) of walsurobustone A (1) in (CD_3_)_2_SO


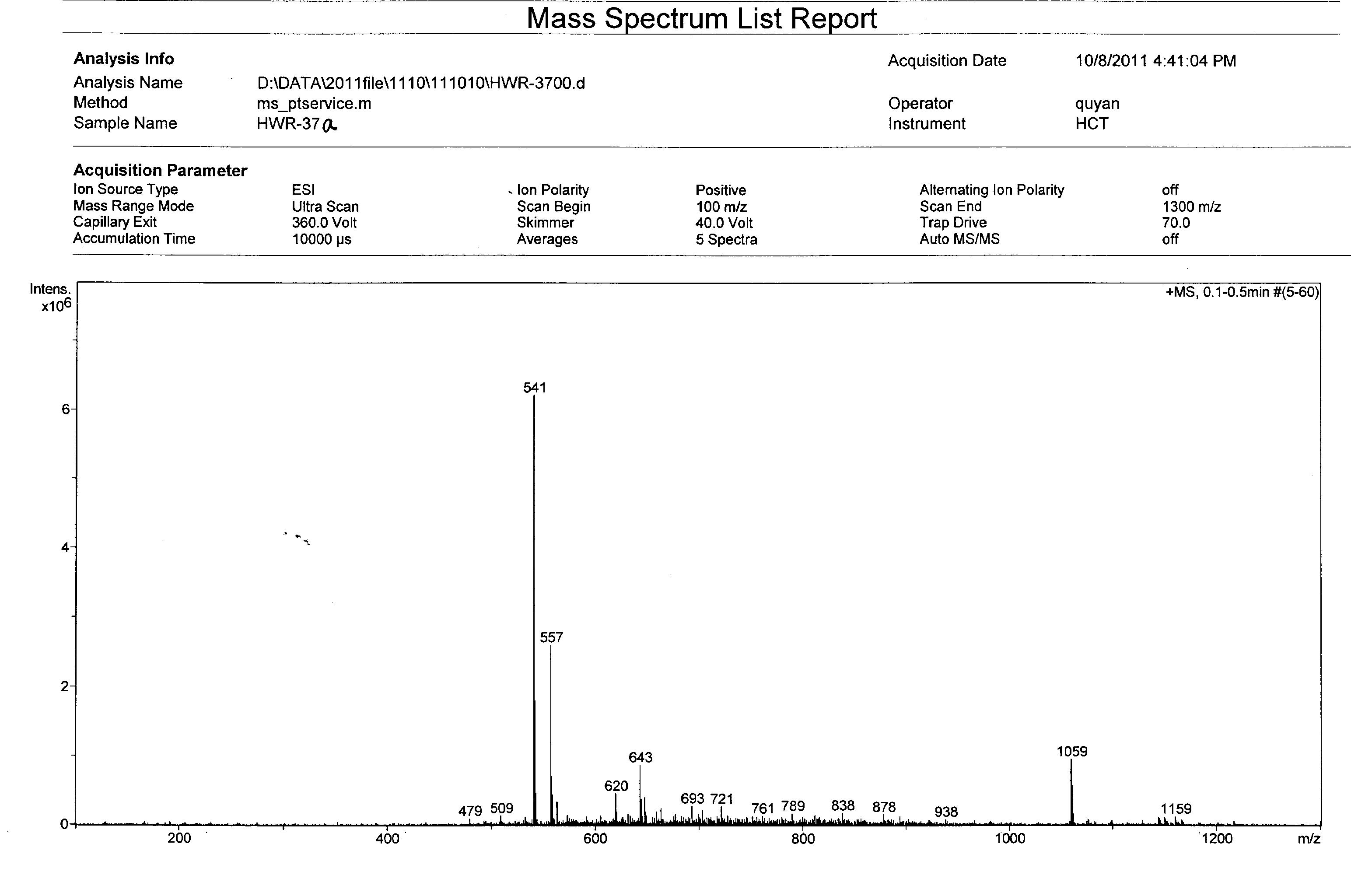
**Figure S7. ESIMS spectrum of walsurobustone A (**1**)**

Figure S8. HRESIMS spectrum of walsurobustone A (1)


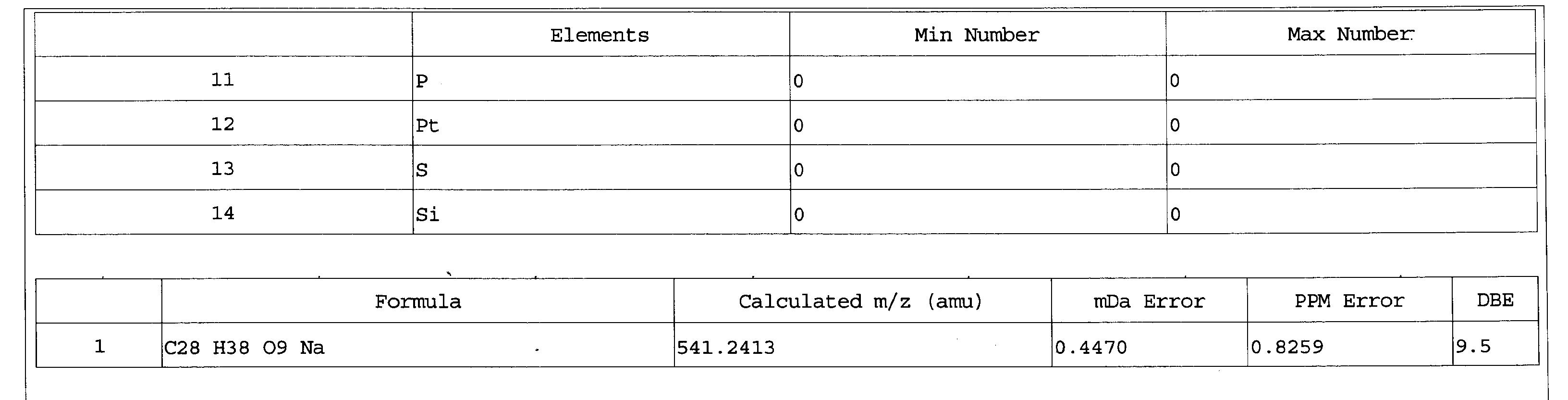

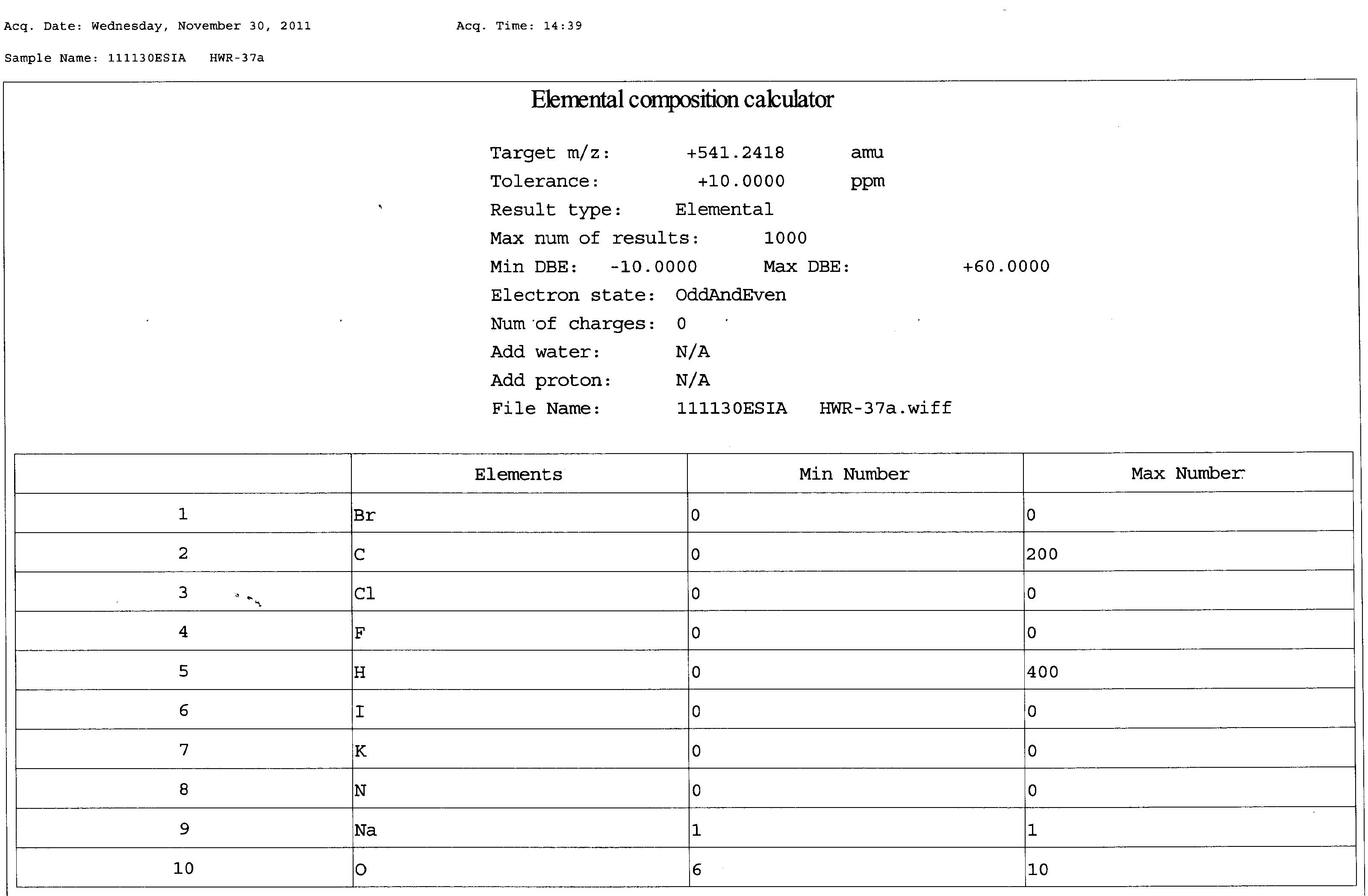


Figure S9. IR (KBr disc) spectrum of walsurobustone A (1)


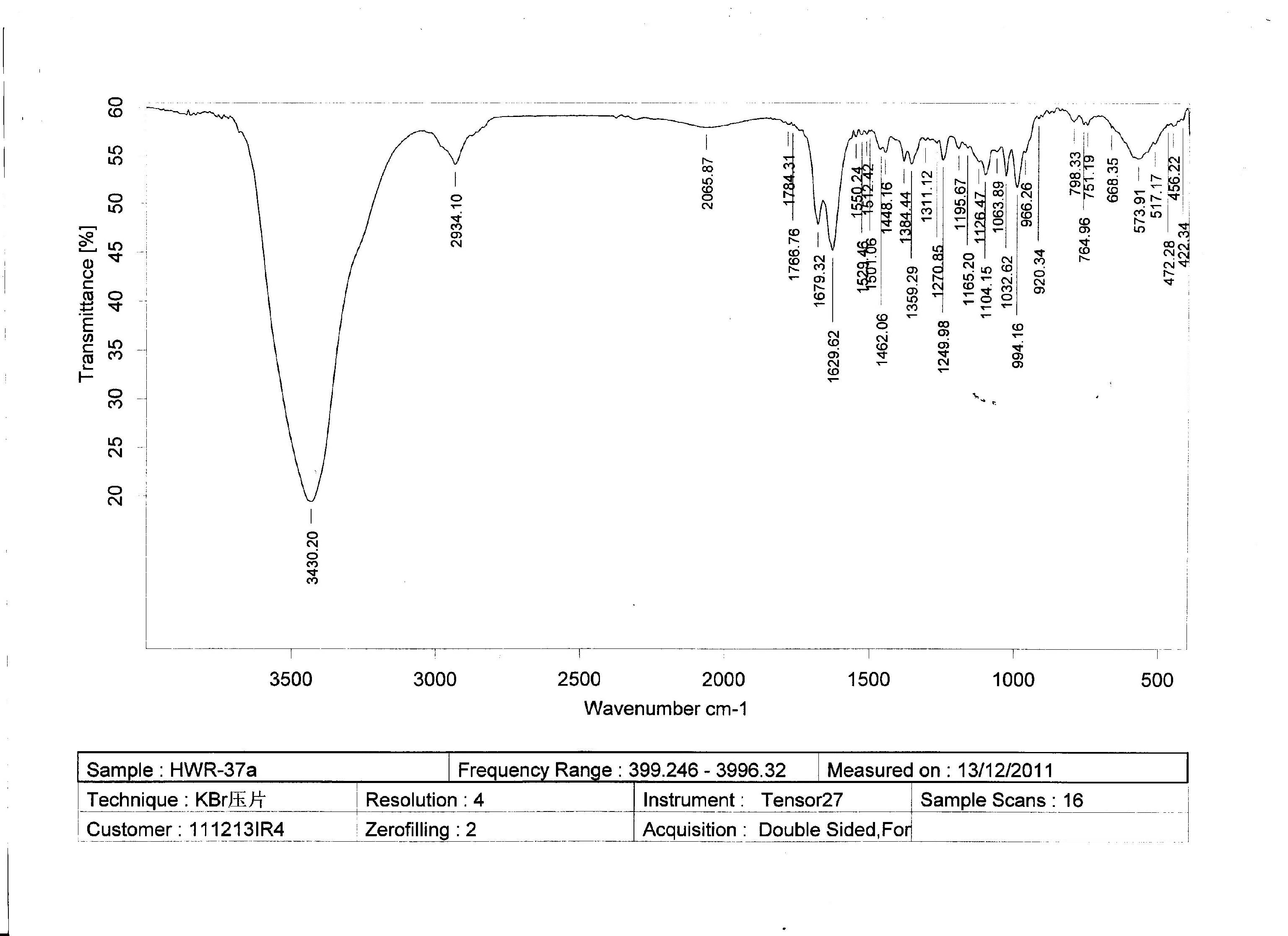


Figure S10.^1^H NMR spectrum (600 MHz) of walsurobustone B (2) in (CD_3_)_2_SO


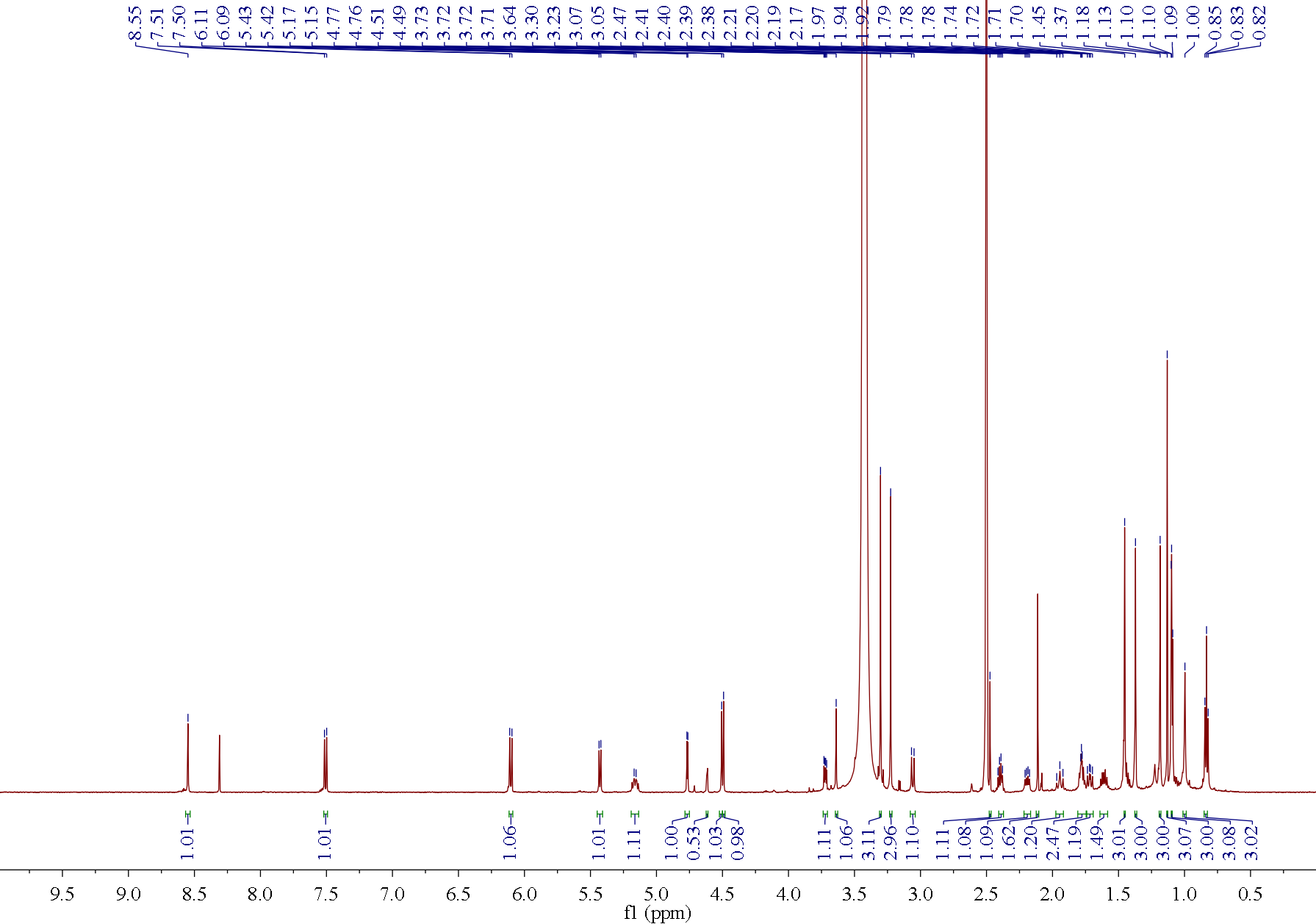


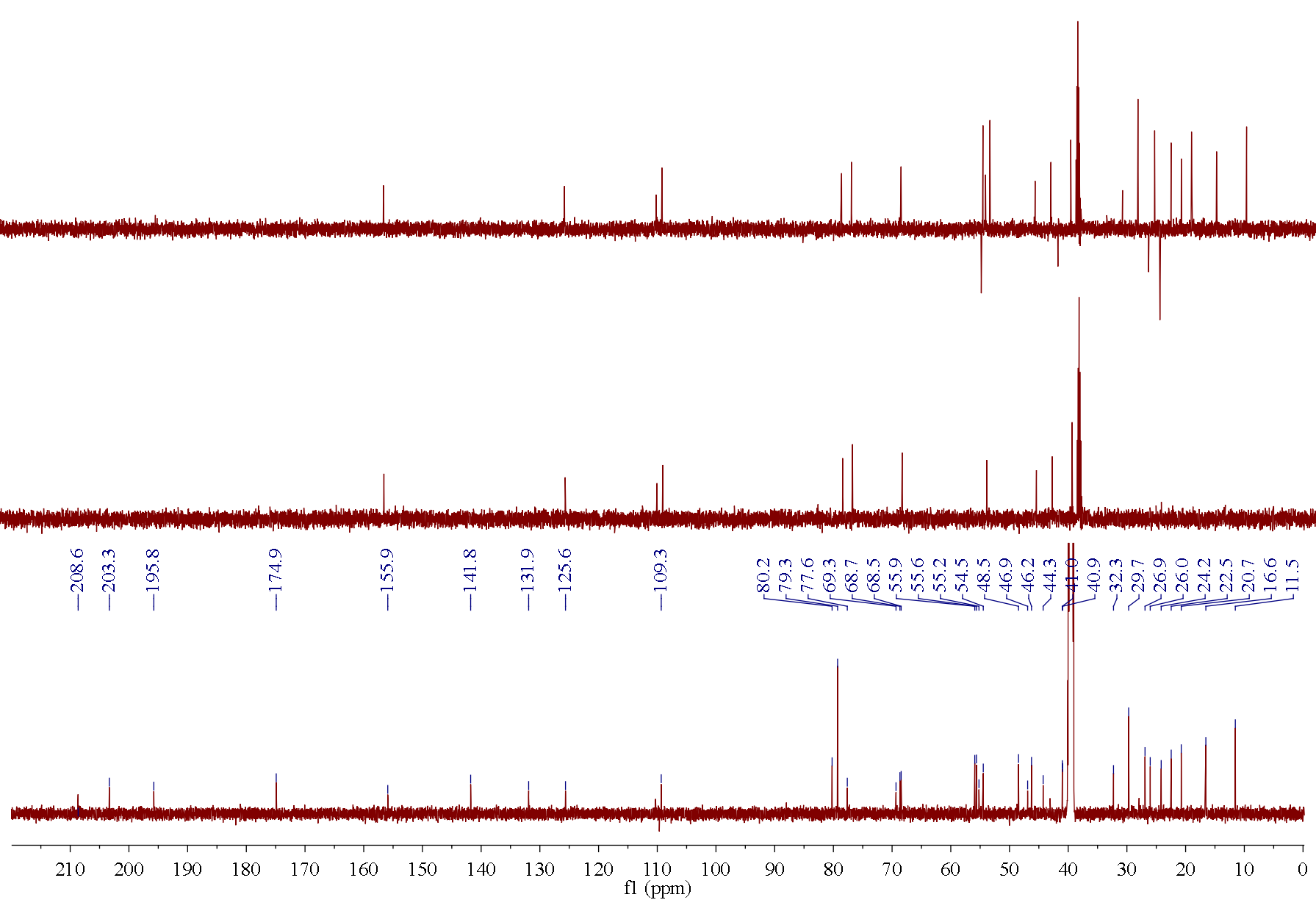
Figure S11.^13^CNMR spectrum (150 MHz) of walsurobustone B (2) in (CD_3_)_2_SO

Figure S12. ^1^H-^1^H COSY spectrum (600 MHz) of walsurobustone B (2) in (CD_3_)_2_SO


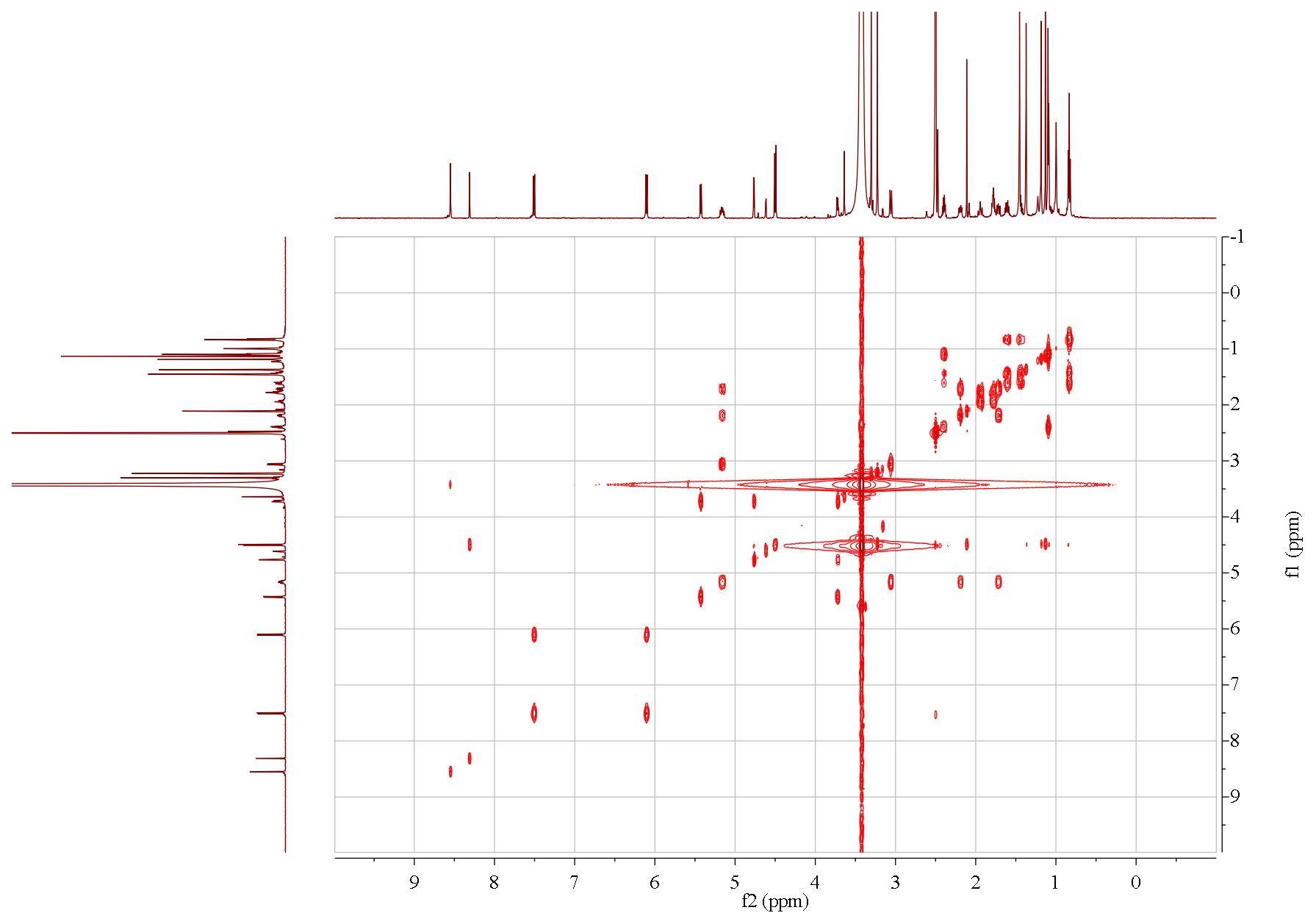


Figure S13. HSQC spectrum (600 MHz) of walsurobustone B (2) in (CD_3_)_2_SO


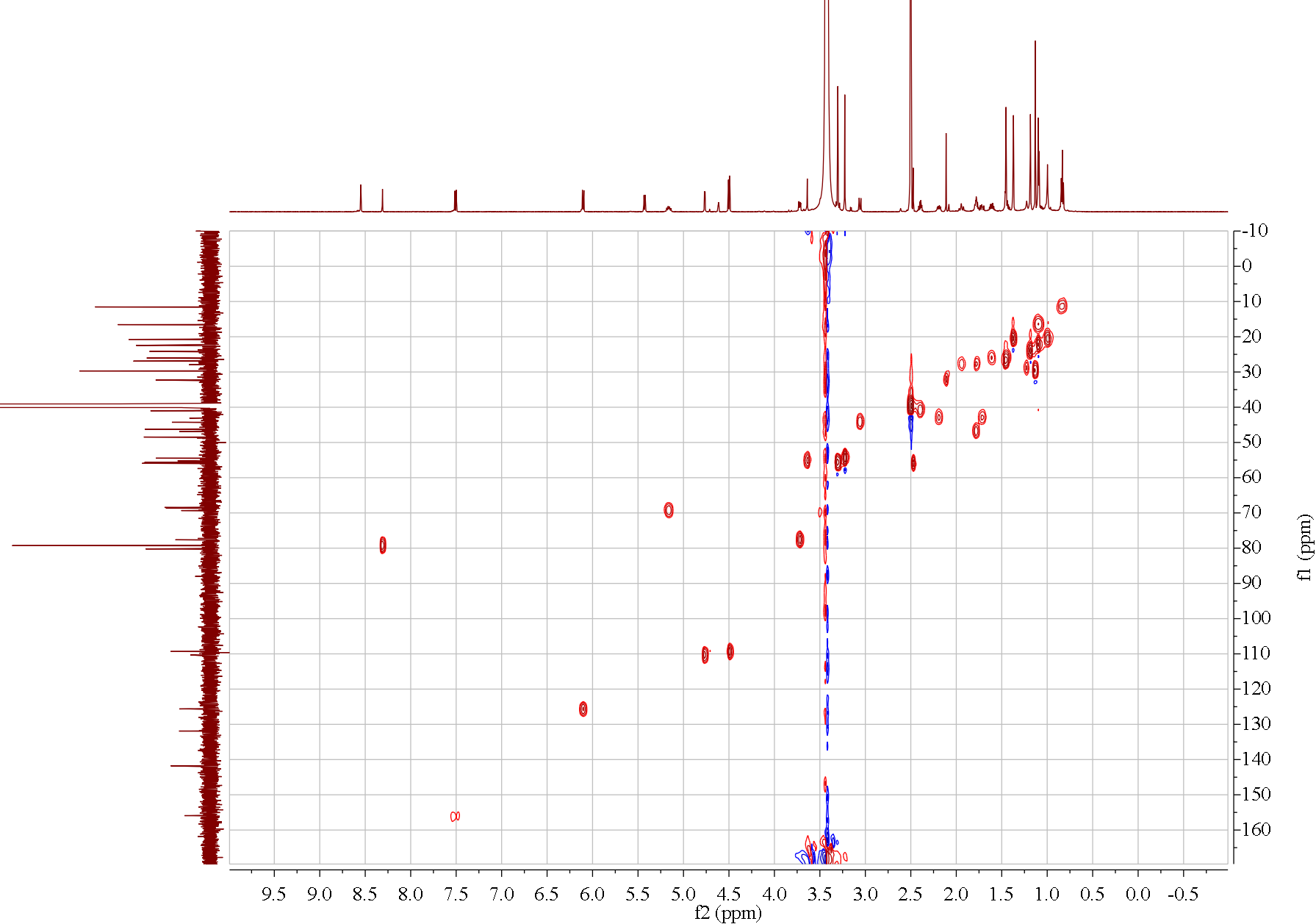
Figure S14. HMBC spectrum (600 MHz) of walsurobustone B (2) in (CD_3_)_2_SO


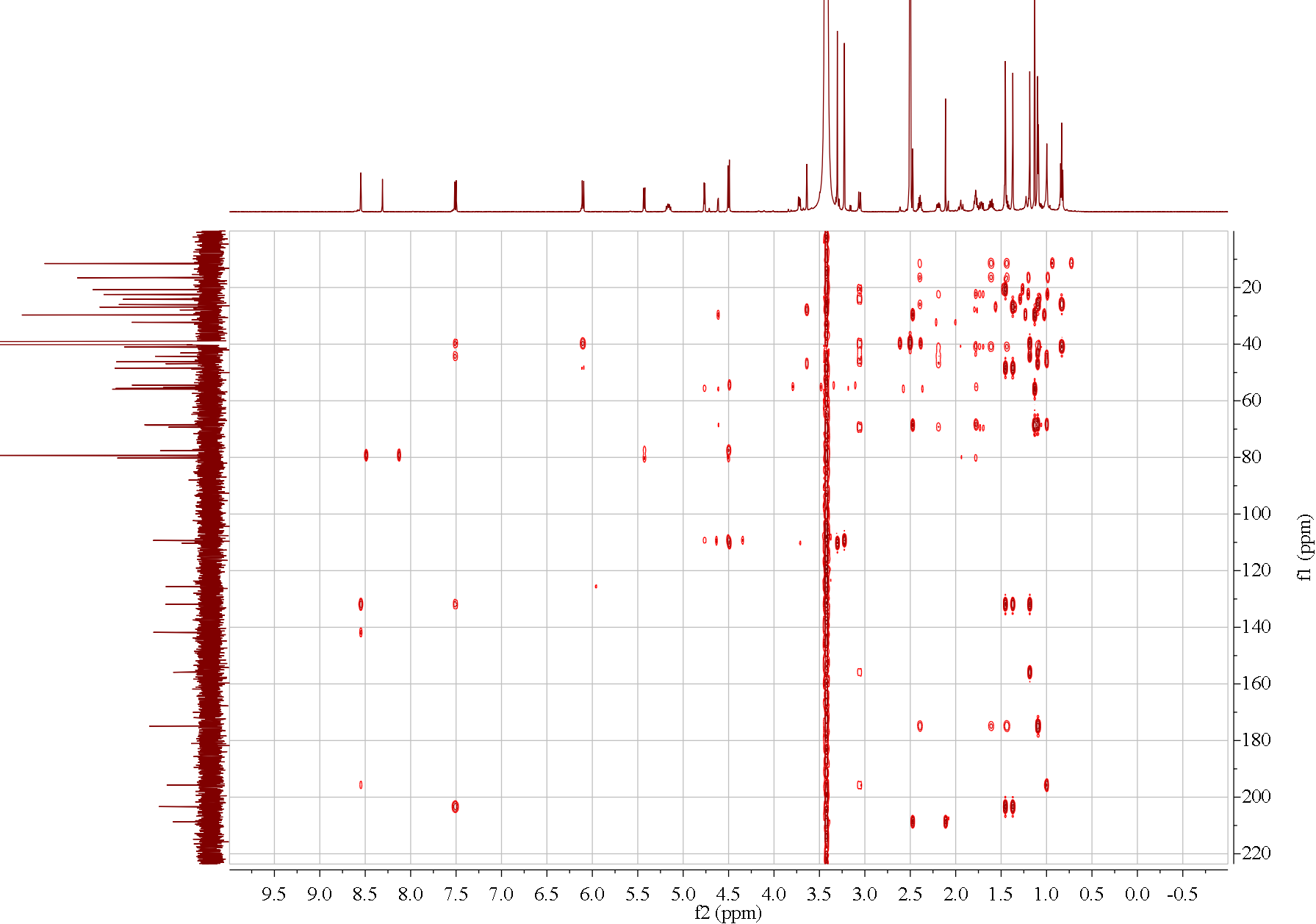


Figure S15. ROESY spectrum (600 MHz) of walsurobustone B (2) in (CD_3_)_2_SO


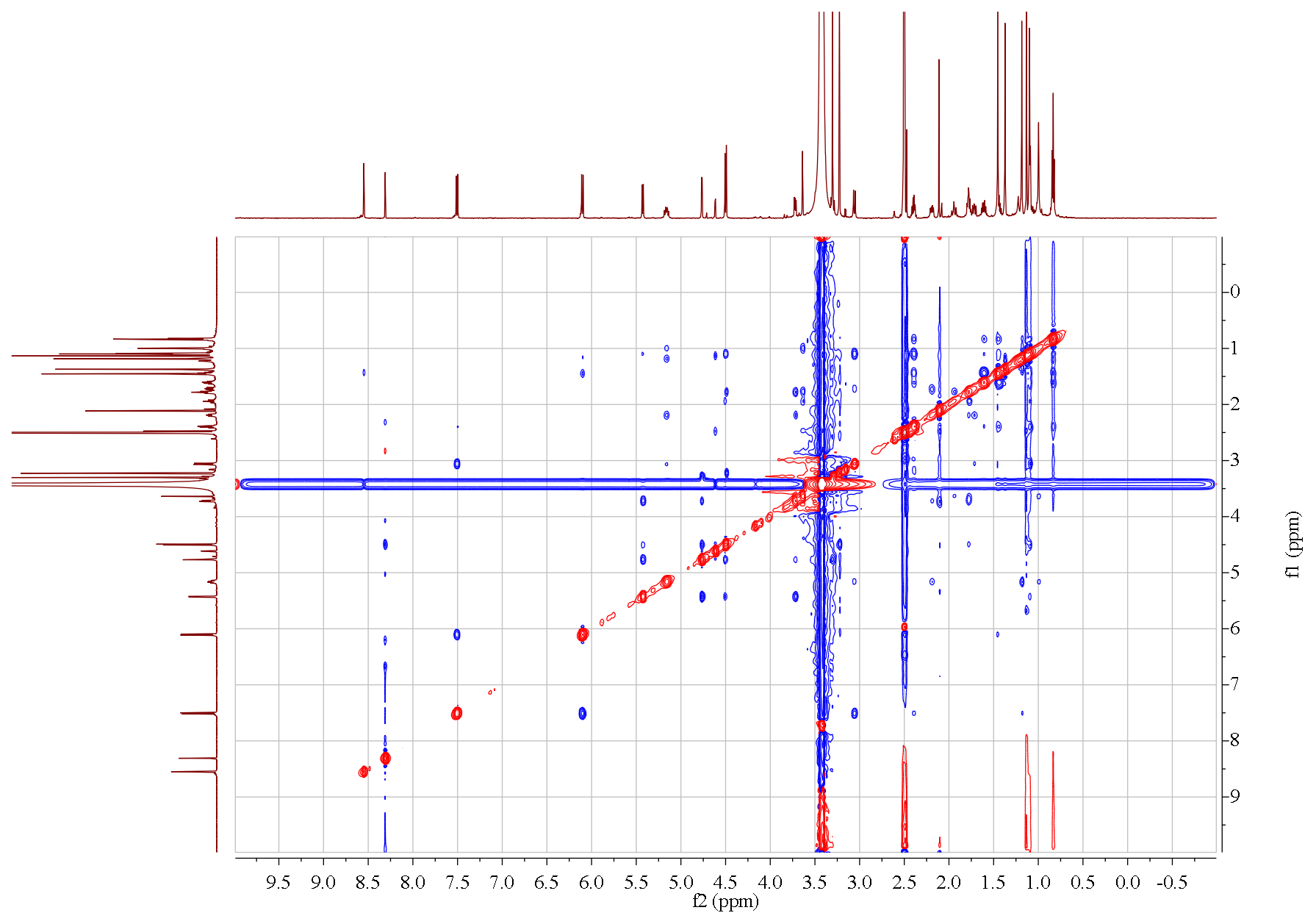
Figure S16. ESIMS spectrum of walsurobustone B (2)


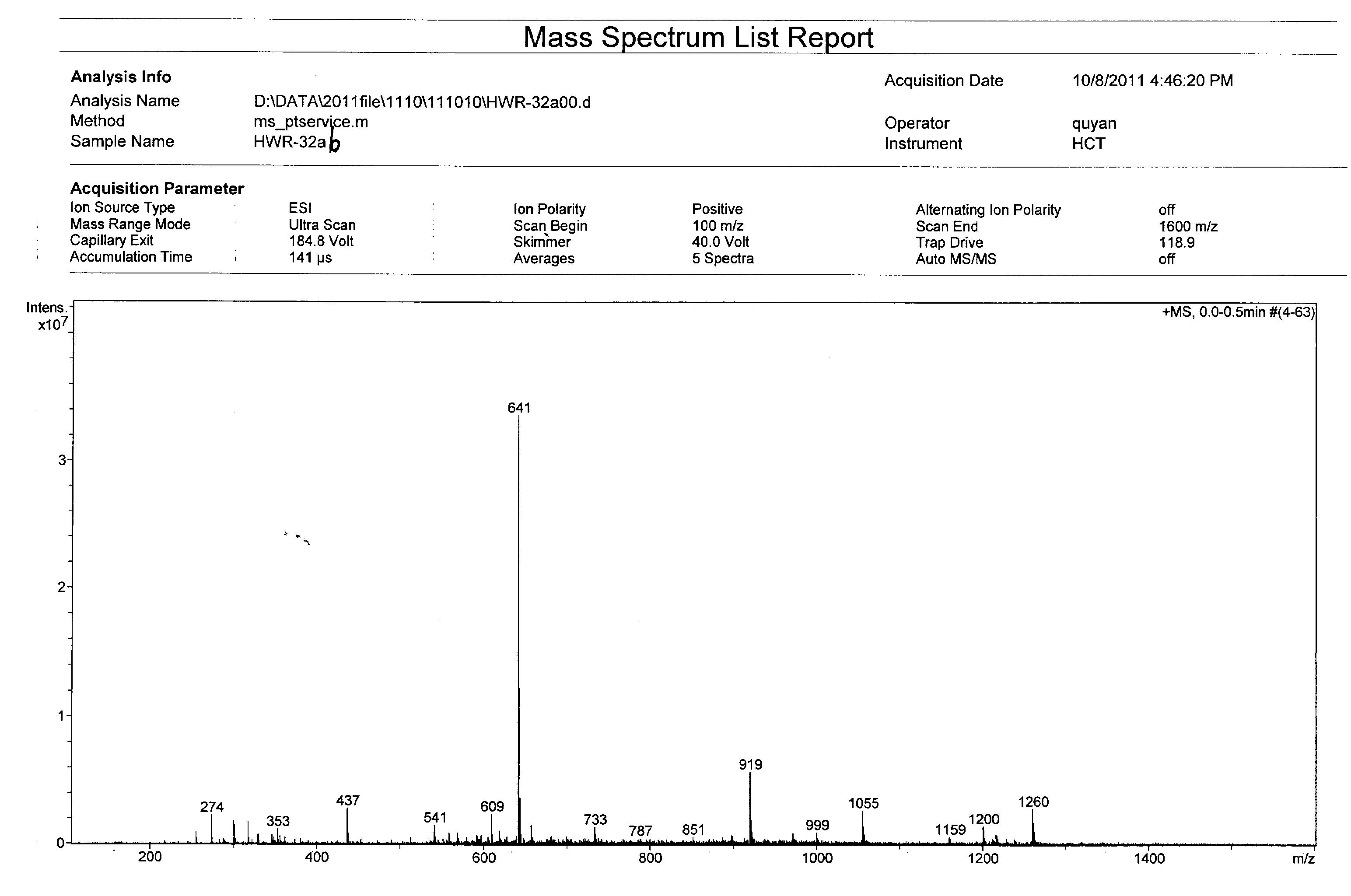


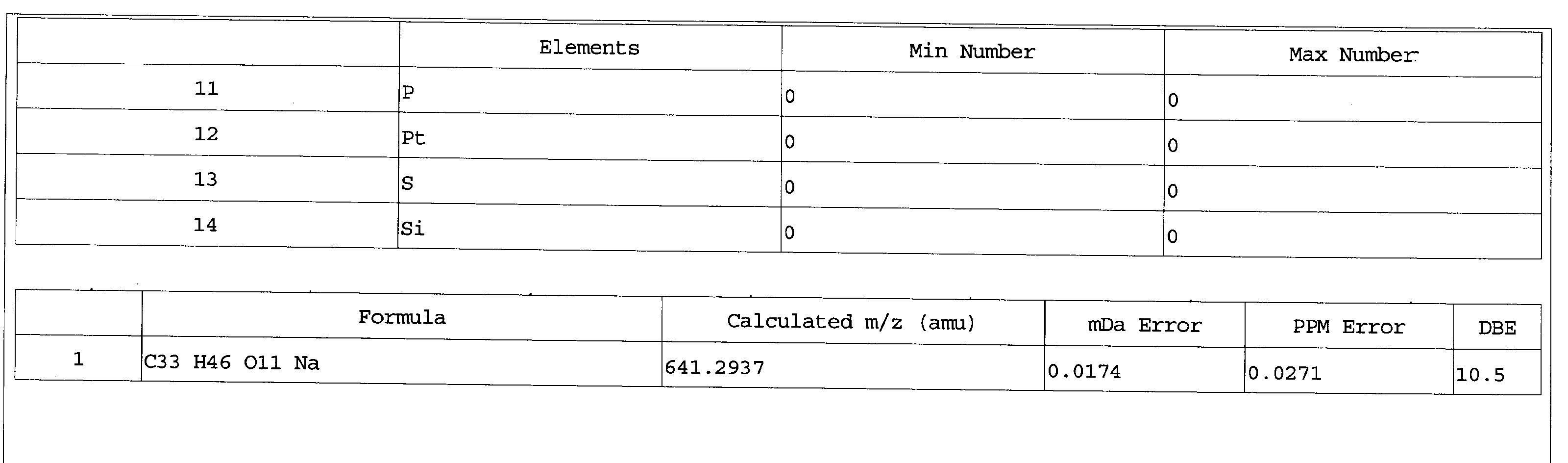

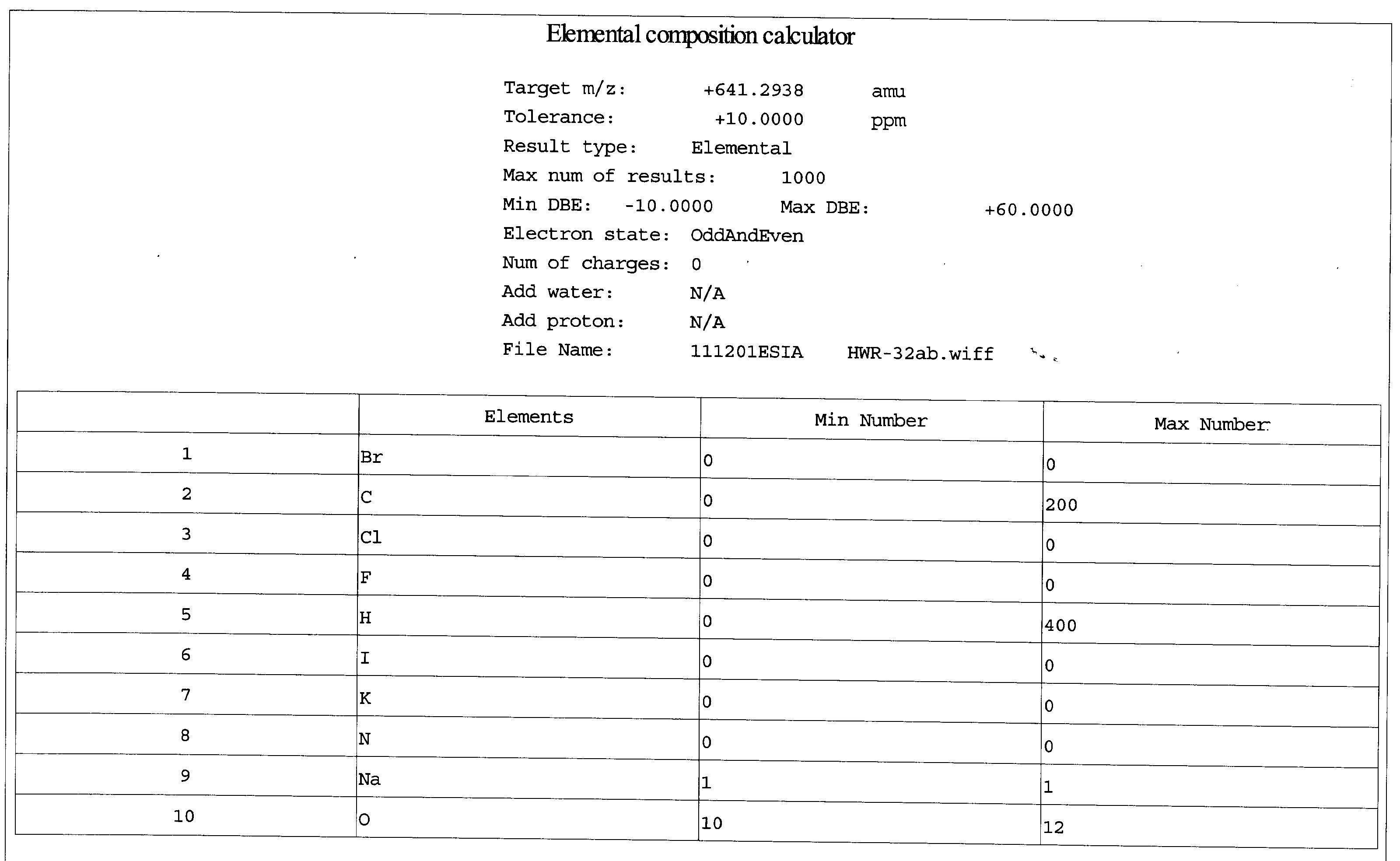
Figure S17. HRESIMS spectrum of walsurobustone B (2)

Figure S18. IR (KBr disc) spectrum of walsurobustone B (2)


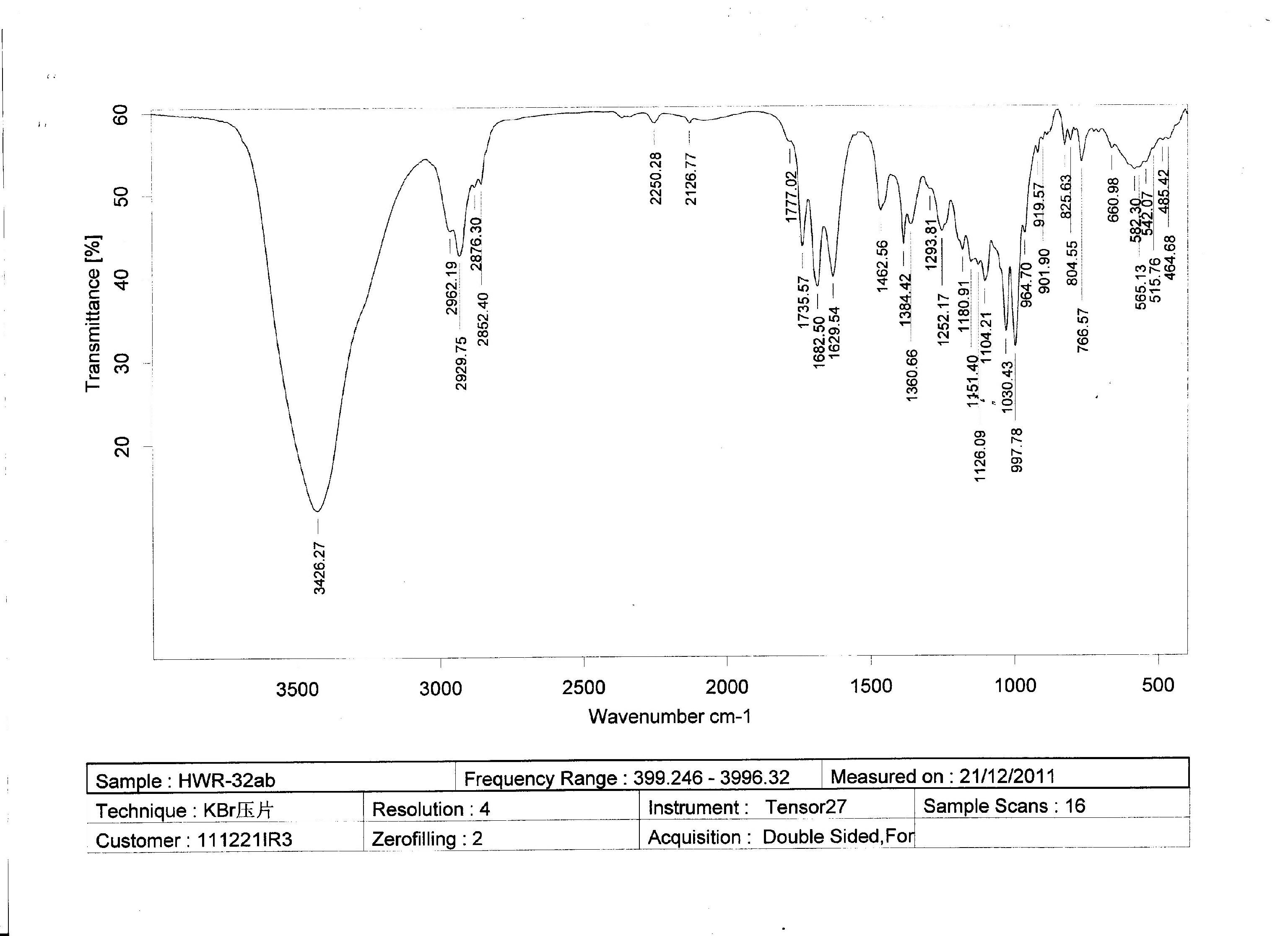


Figure S19.^1^H NMR spectrum (400 MHz) of walsurobustone C (3) in CDCl_3_


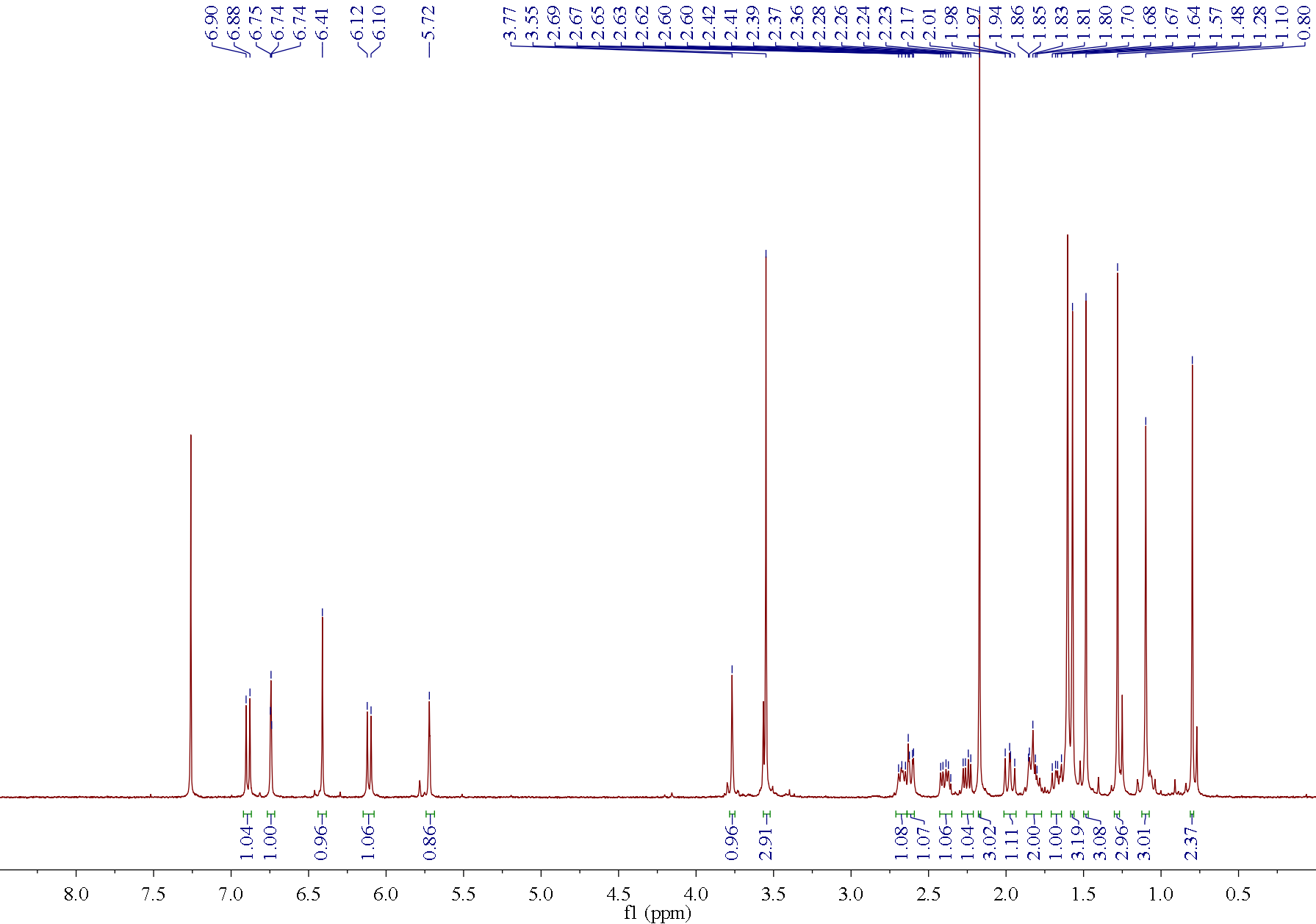


Figure S20.^13^CNMR spectrum (100 MHz) of walsurobustone C(3) in CDCl_3_


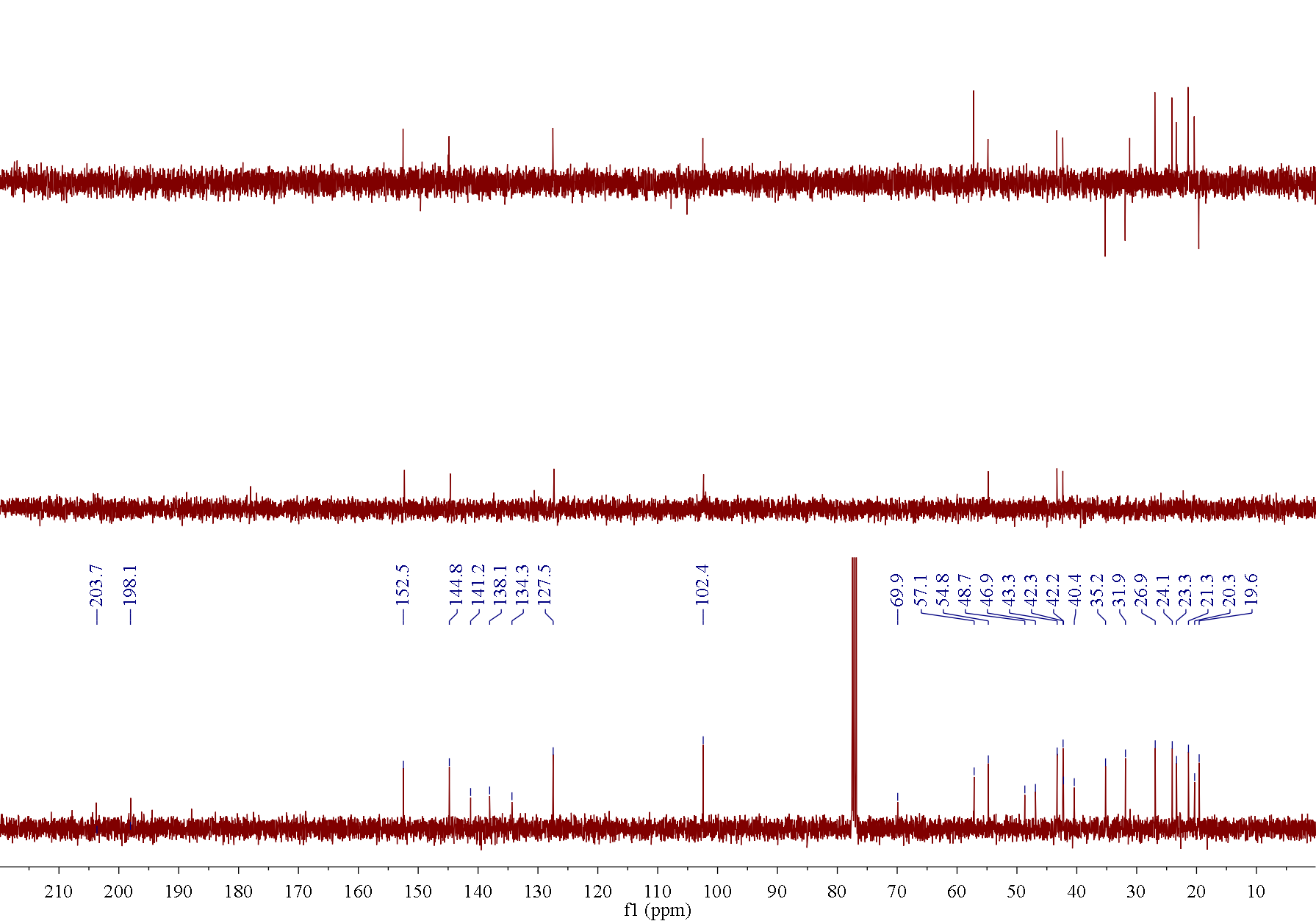


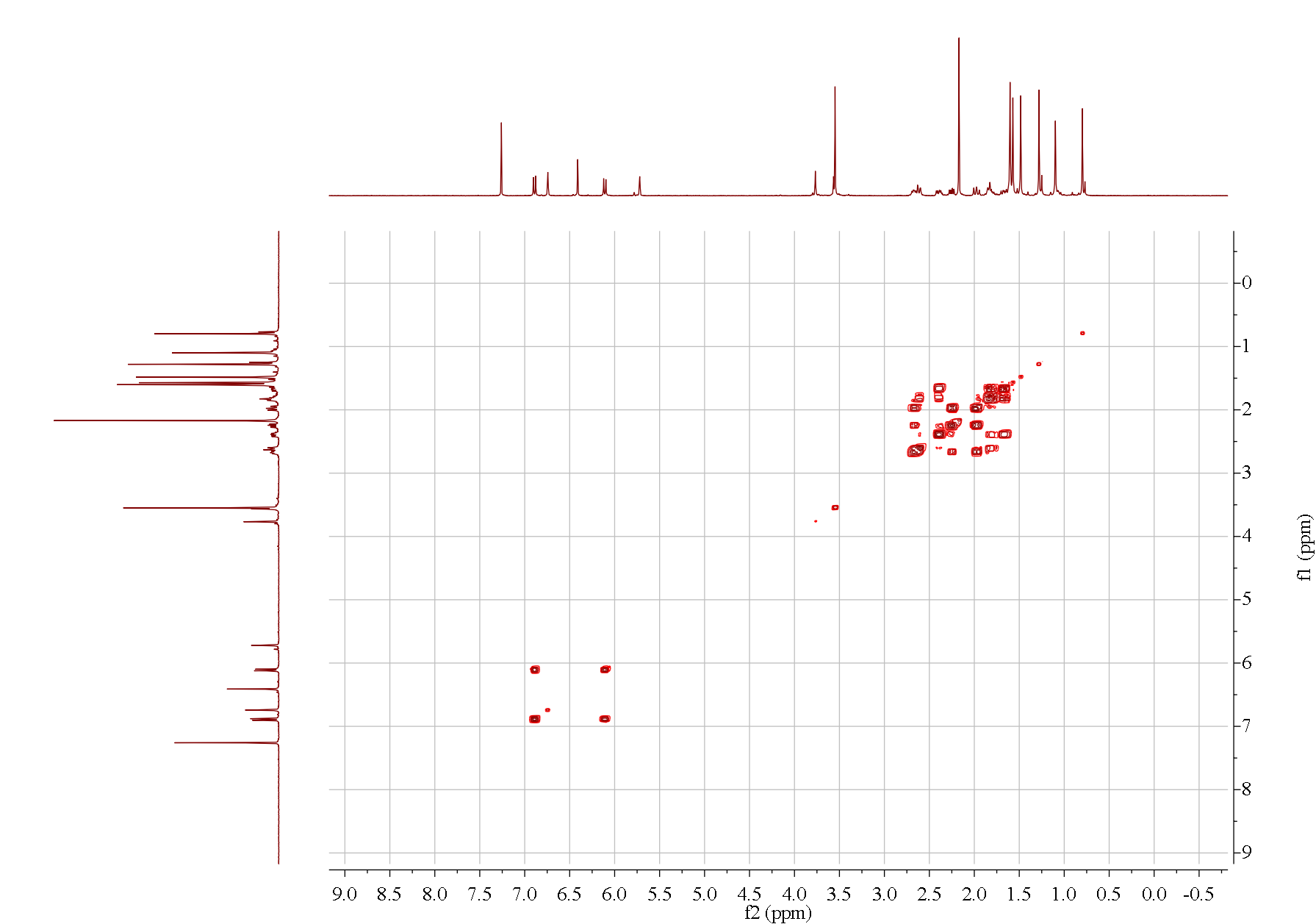
Figure S21. ^1^H-^1^H COSY spectrum (500 MHz) of walsurobustone C (3) in CDCl_3_

Figure S22. HSQC spectrum (500 MHz) of walsurobustone C (3) in CDCl_3_


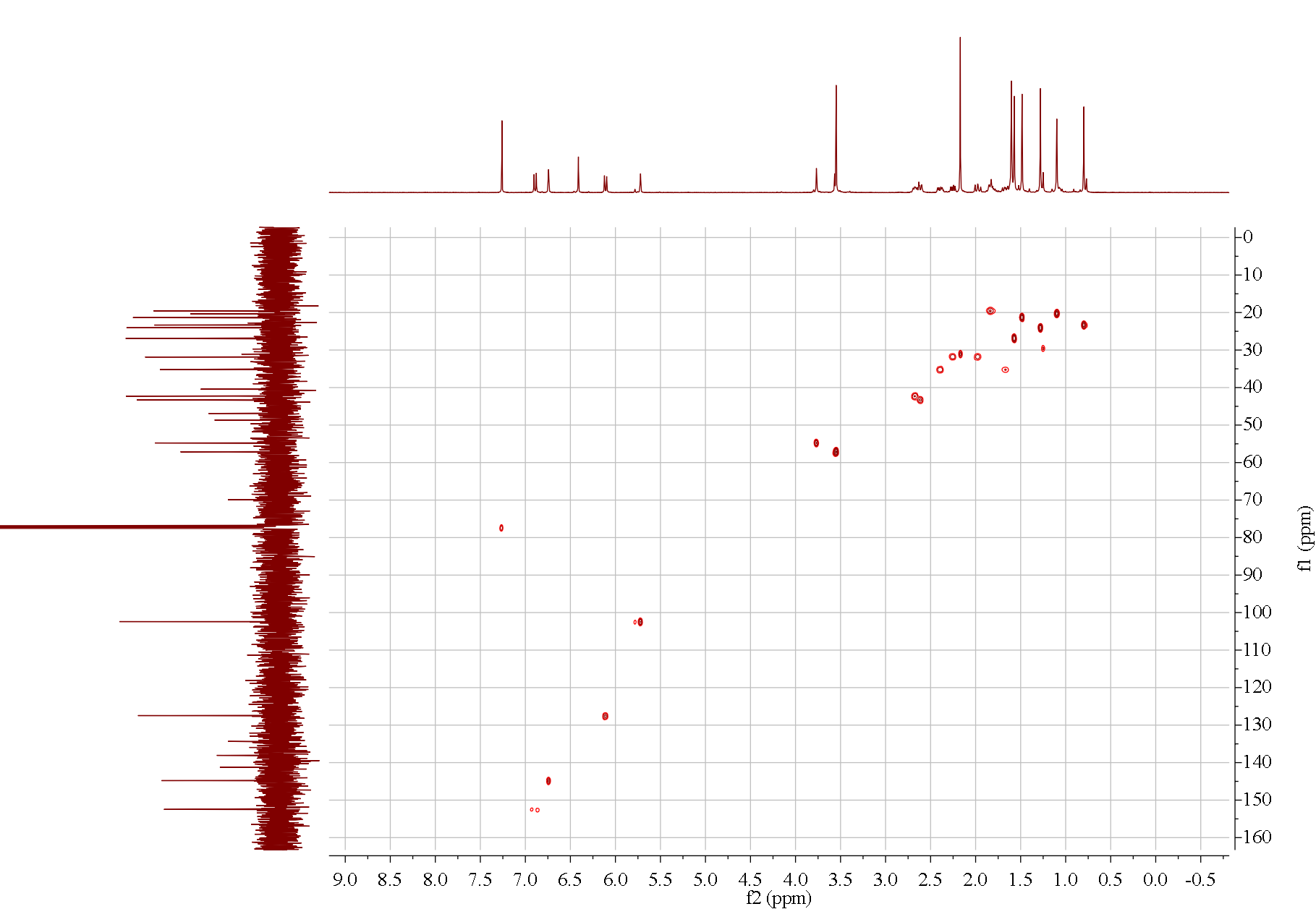


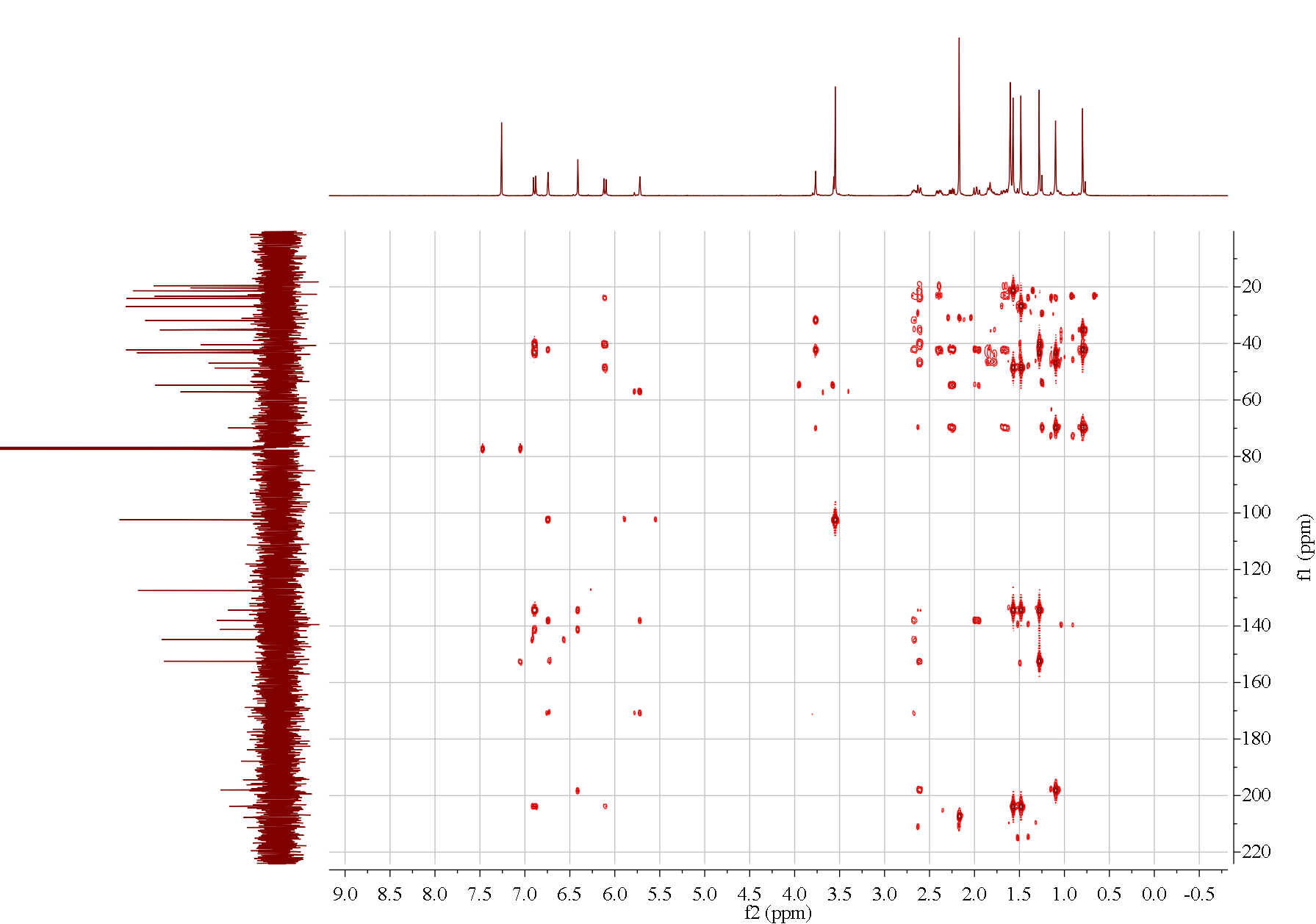
Figure S23. HMBC spectrum (500 MHz) of walsurobustone C (3) in CDCl_3_

Figure S24. ROESY spectrum (500 MHz) of walsurobustone C (3) in CDCl_3_


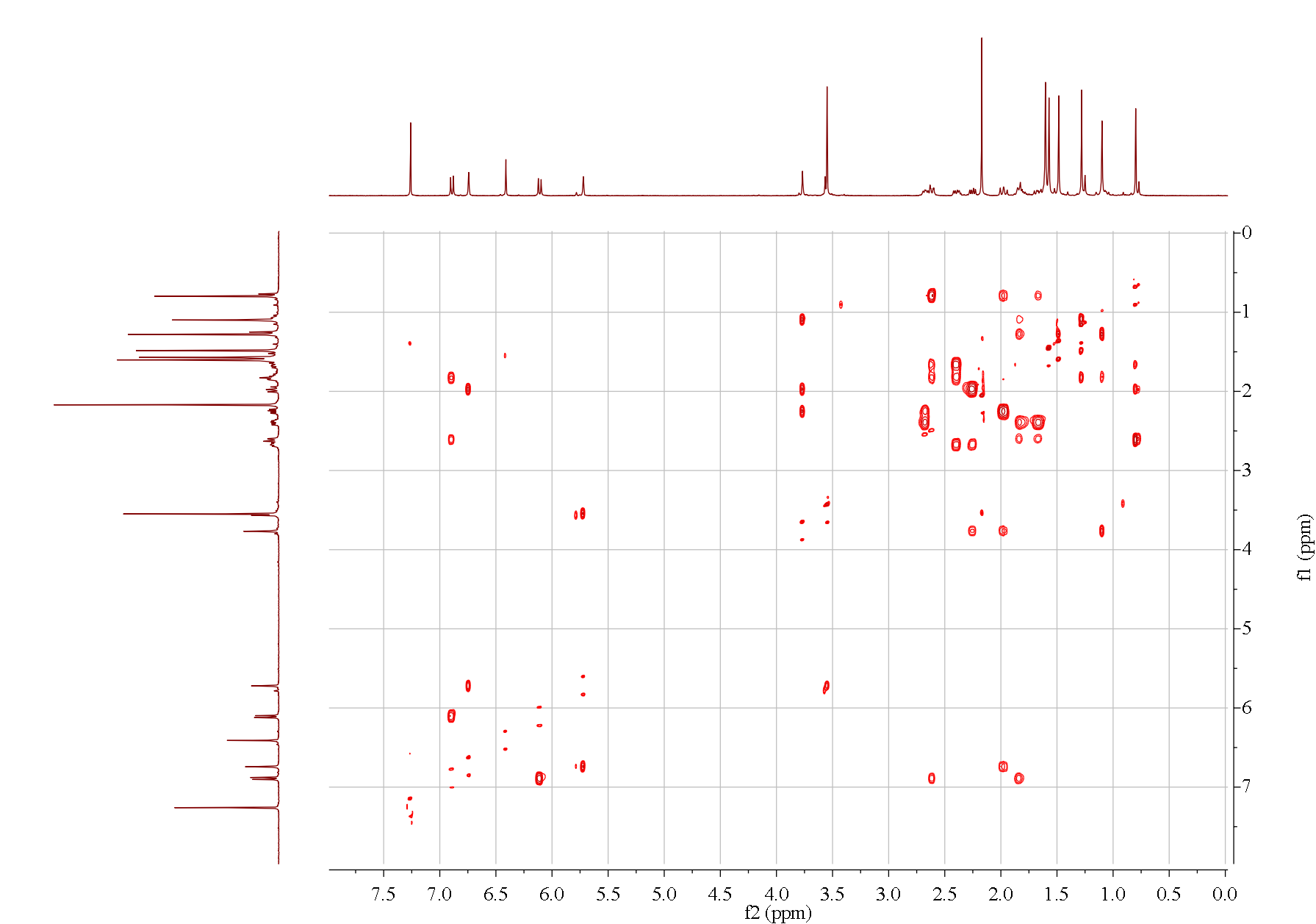


Figure S25. ESIMS spectrum of walsurobustone C (3)


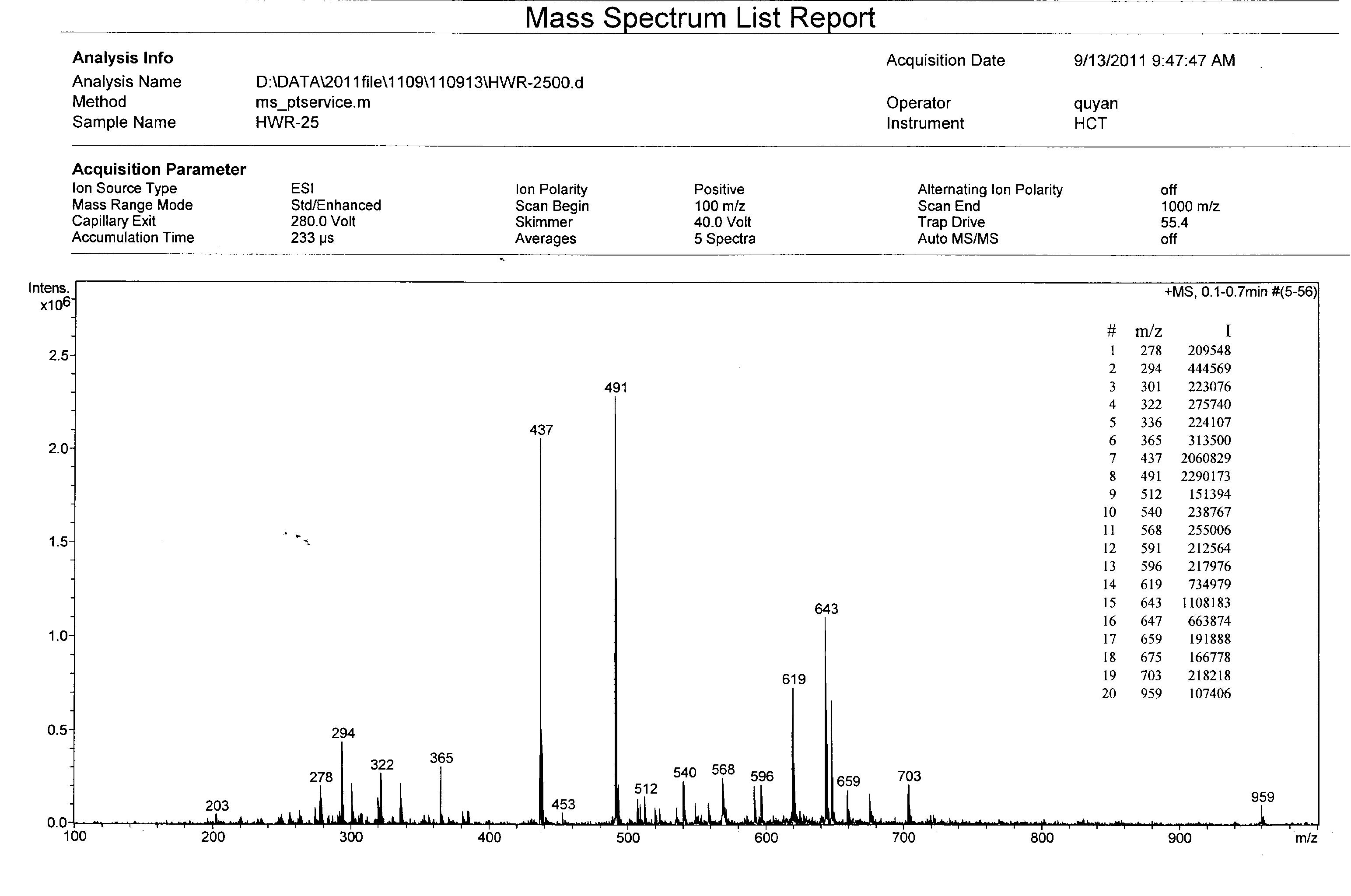
Figure S26. HRESIMS spectrum of walsurobustone C (3)


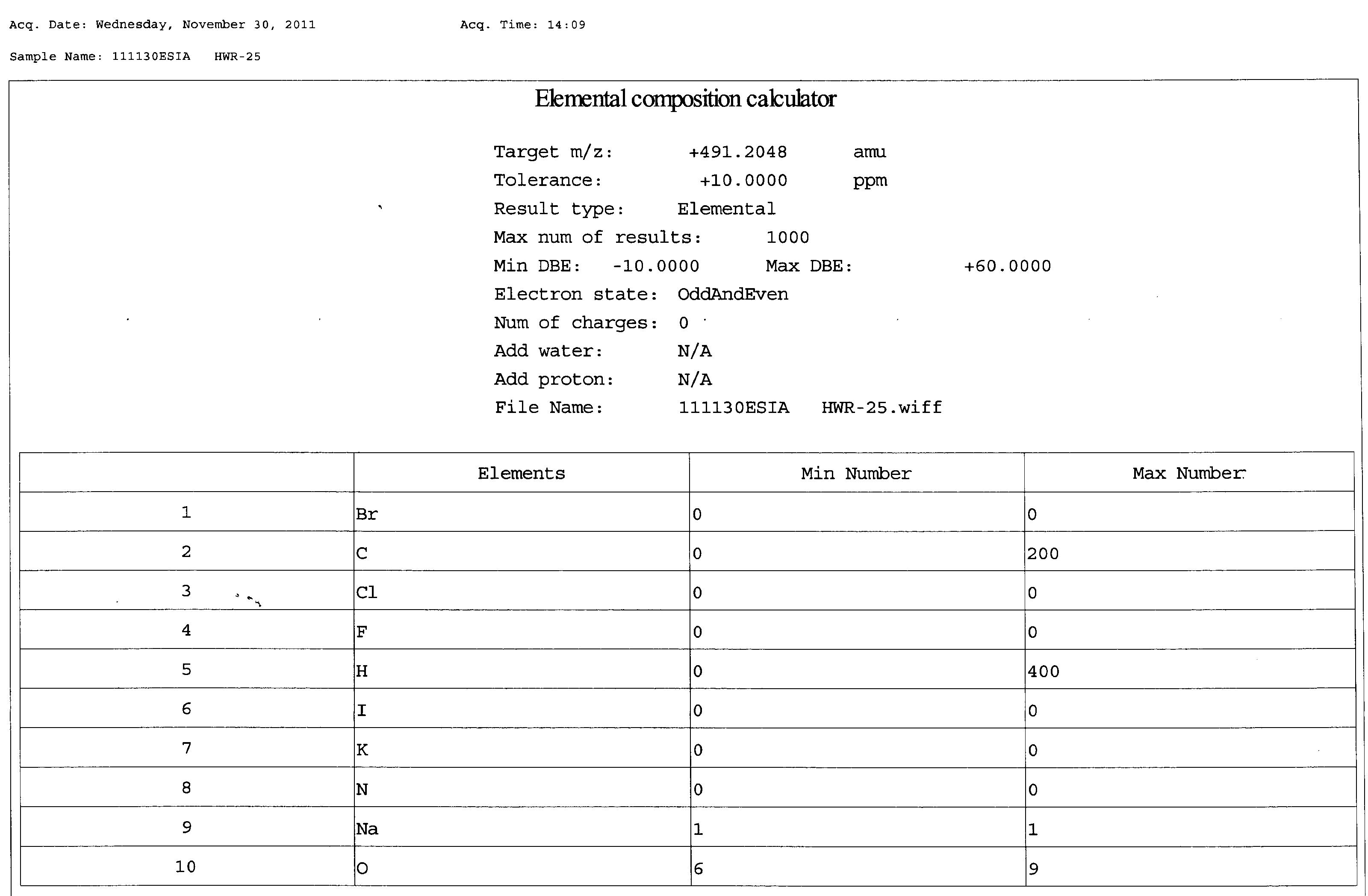

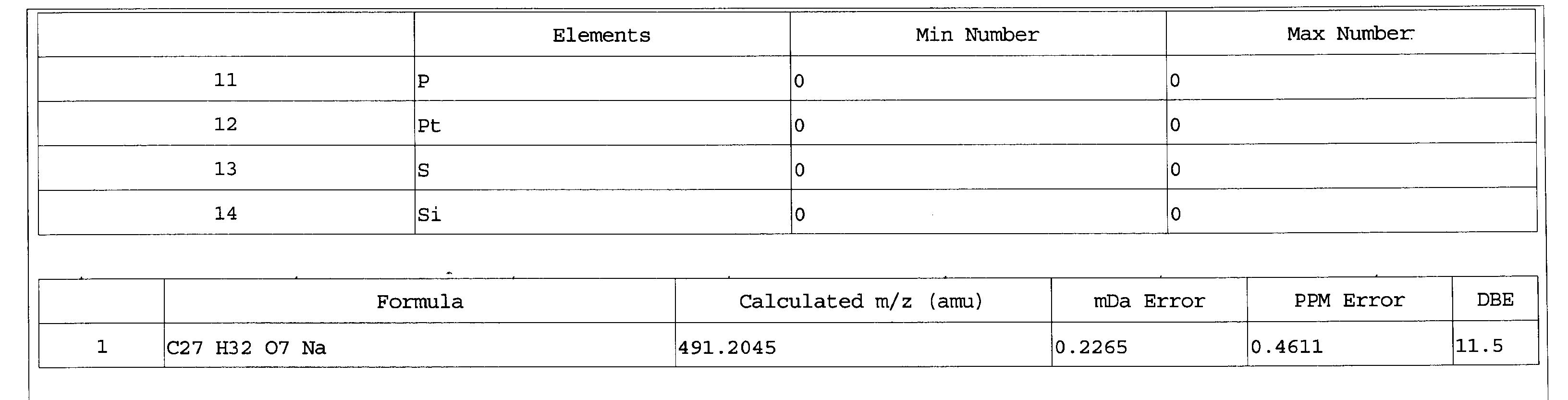


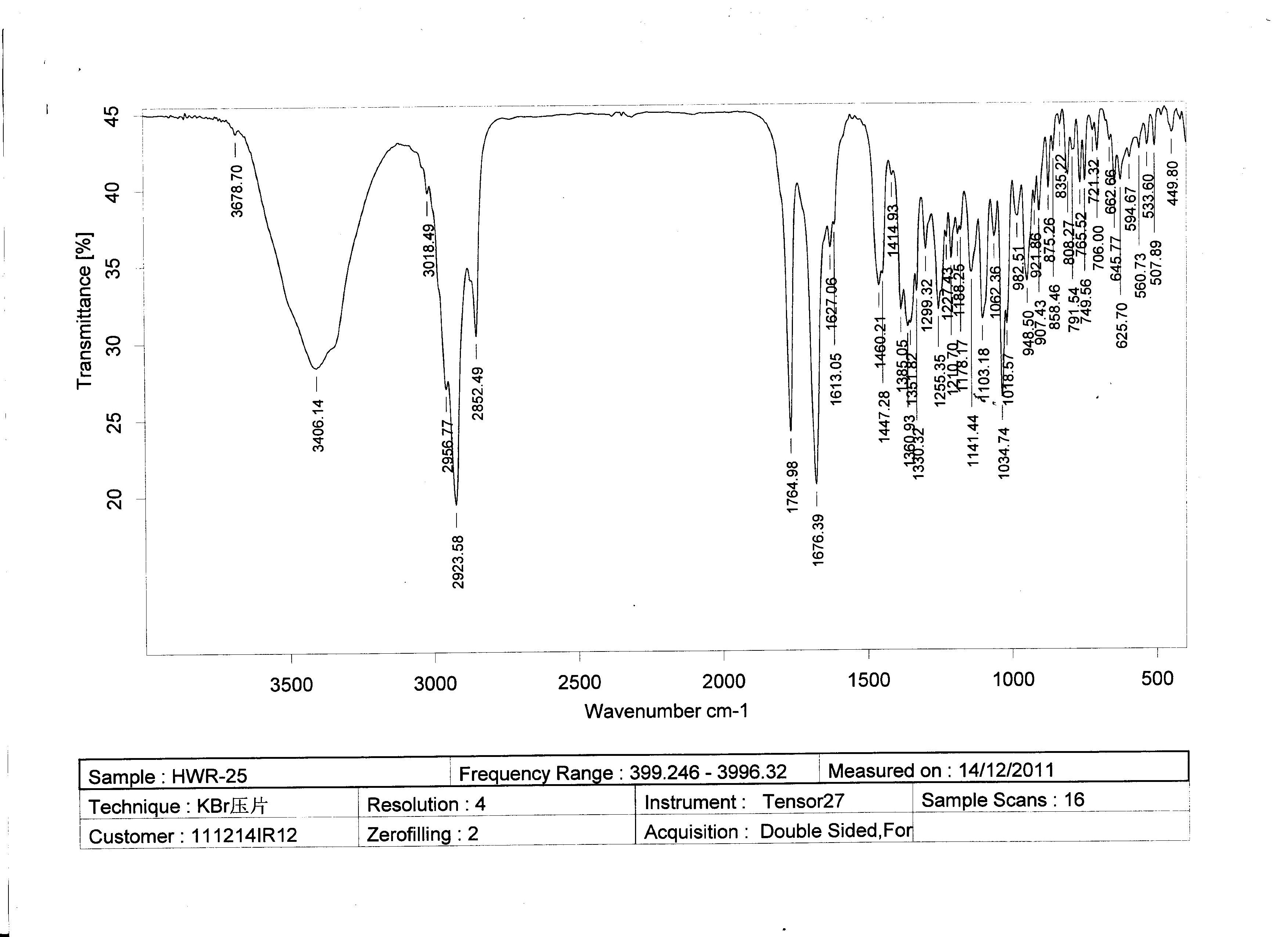
Figure S27. IR (KBr disc) spectrum of walsurobustone C (3)

Figure S28. ^1^H NMR spectrum (500 MHz) of walsurobustone D (4) in (CD_3_)_2_SO


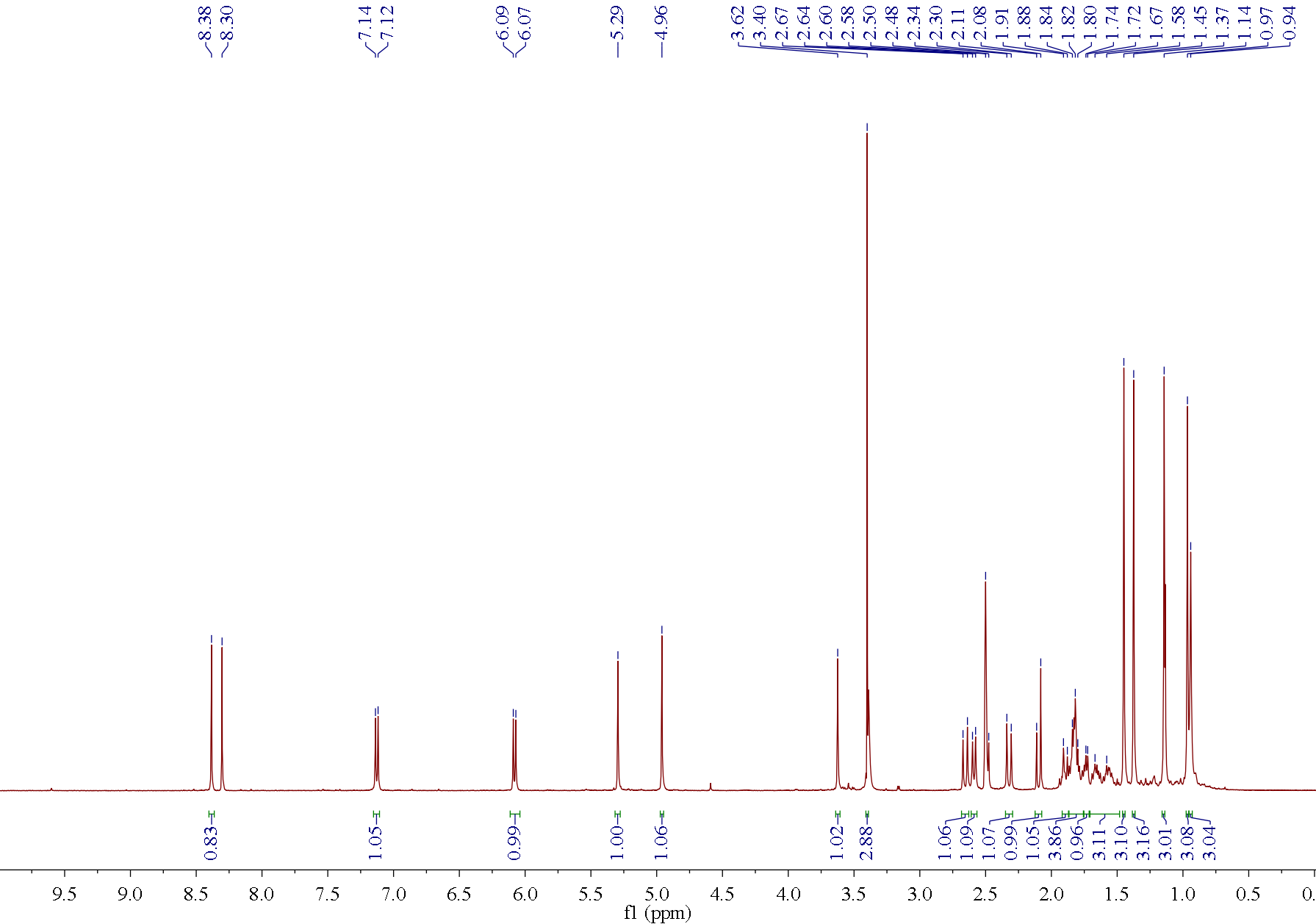


Figure S29. ^13^C NMR spectrum (100 MHz) of walsurobustone D (4) in (CD_3_)_2_SO


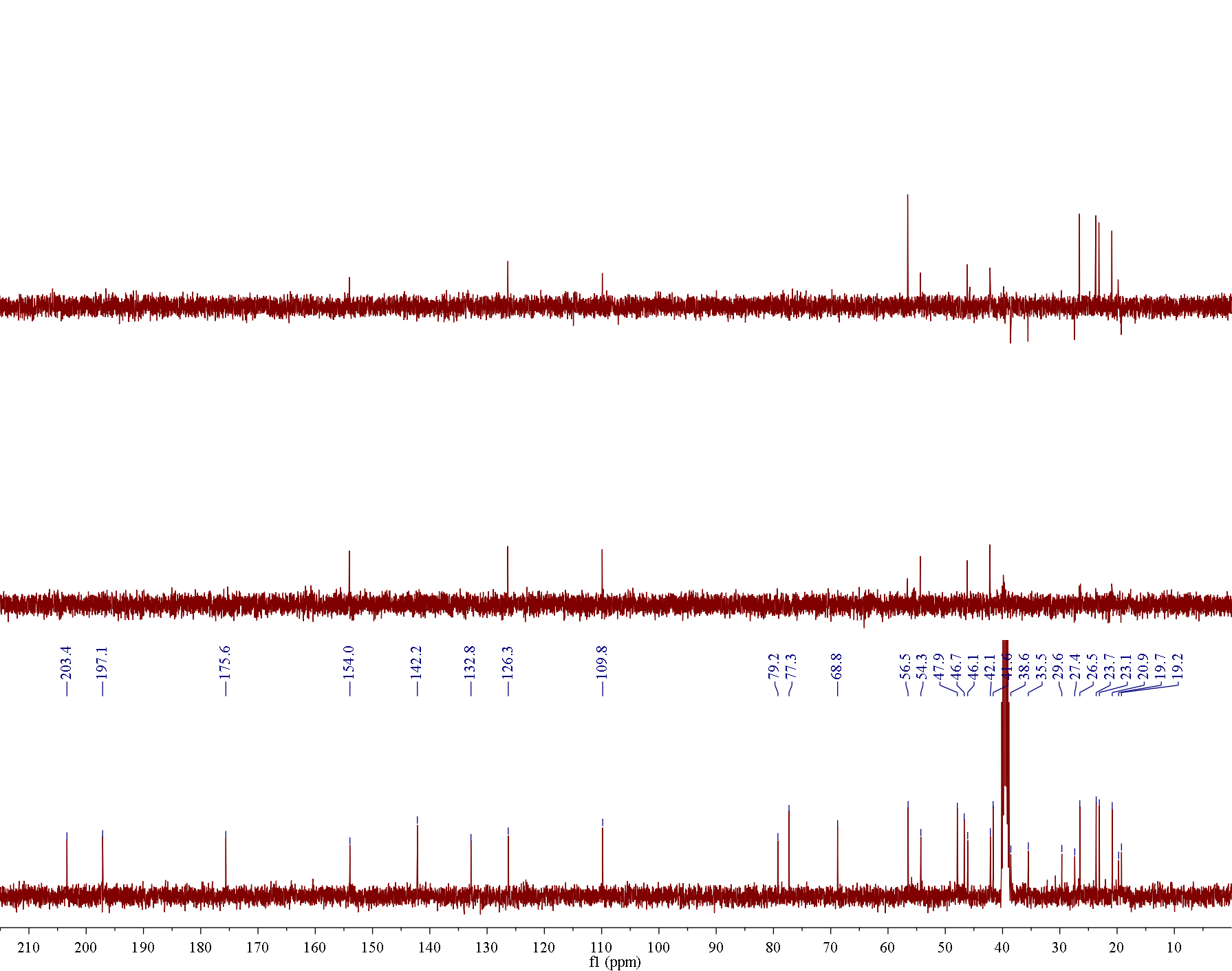


Figure S30. ^1^H-^1^H COSY spectrum (500 MHz) of walsurobustone D (4) in (CD_3_)_2_SO


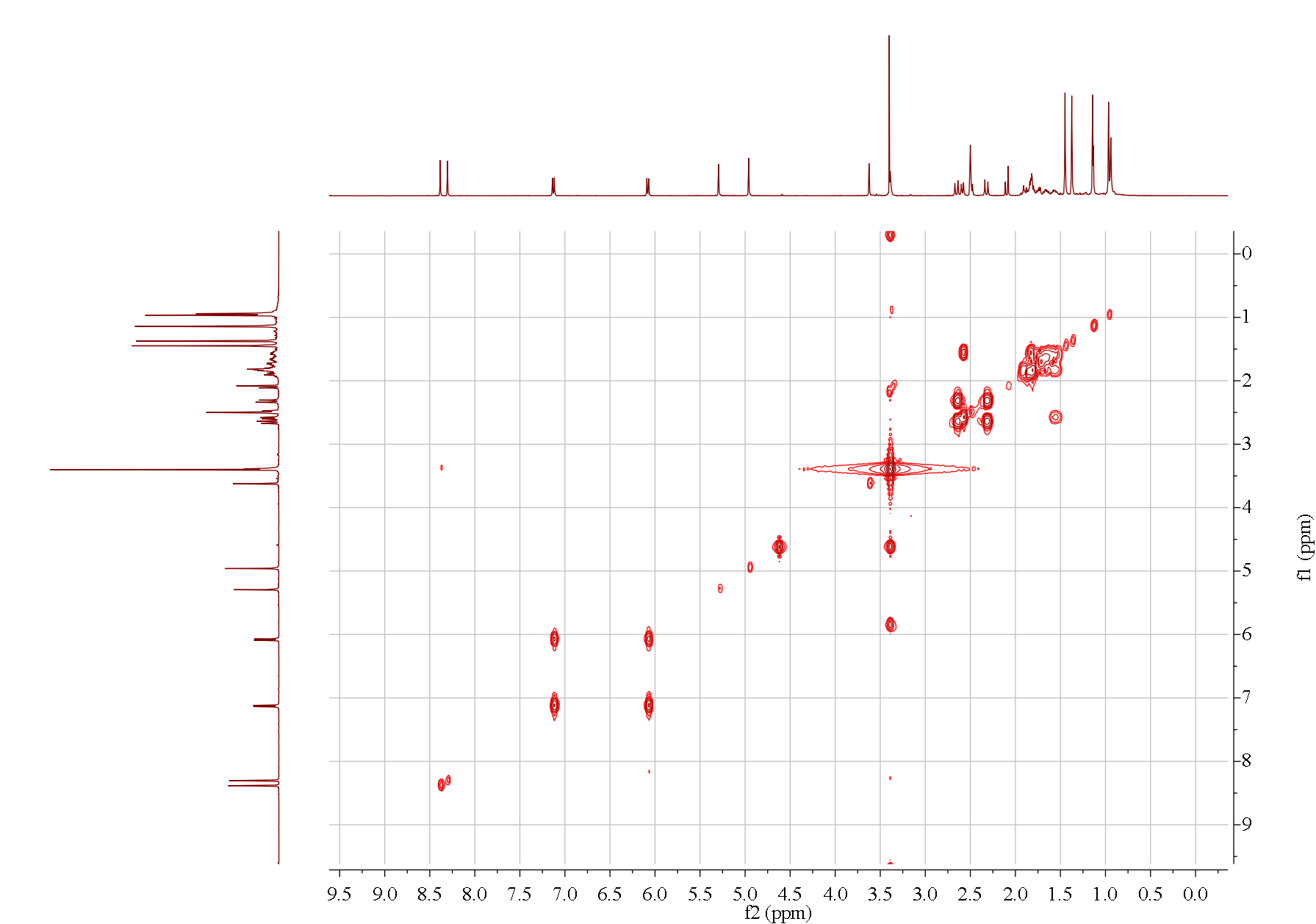


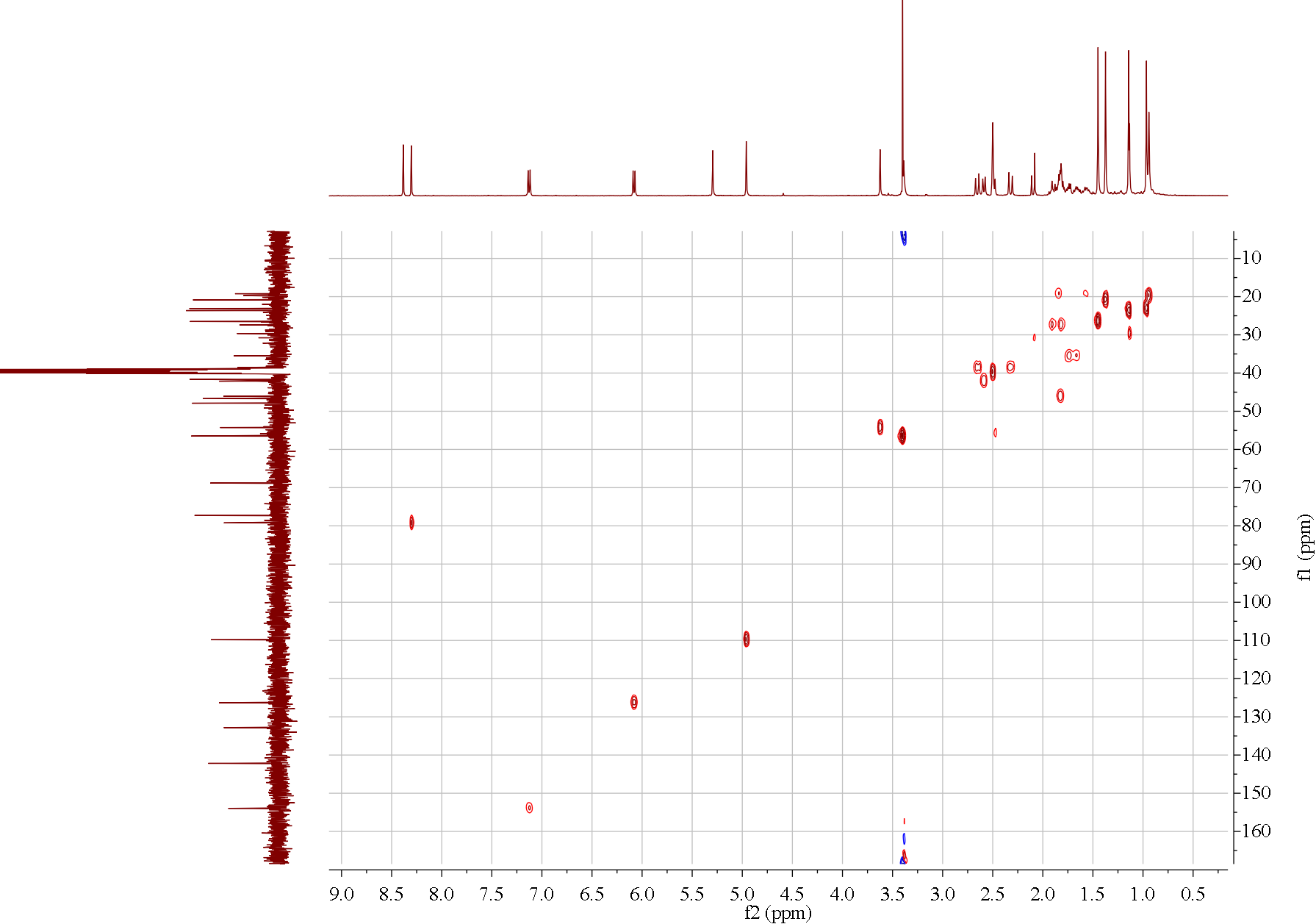
Figure S31. HSQC spectrum (500 MHz) of walsurobustone D (4) in (CD_3_)_2_SO

Figure S32. HMBC spectrum (500 MHz) of walsurobustone D (4) in (CD_3_)_2_SO


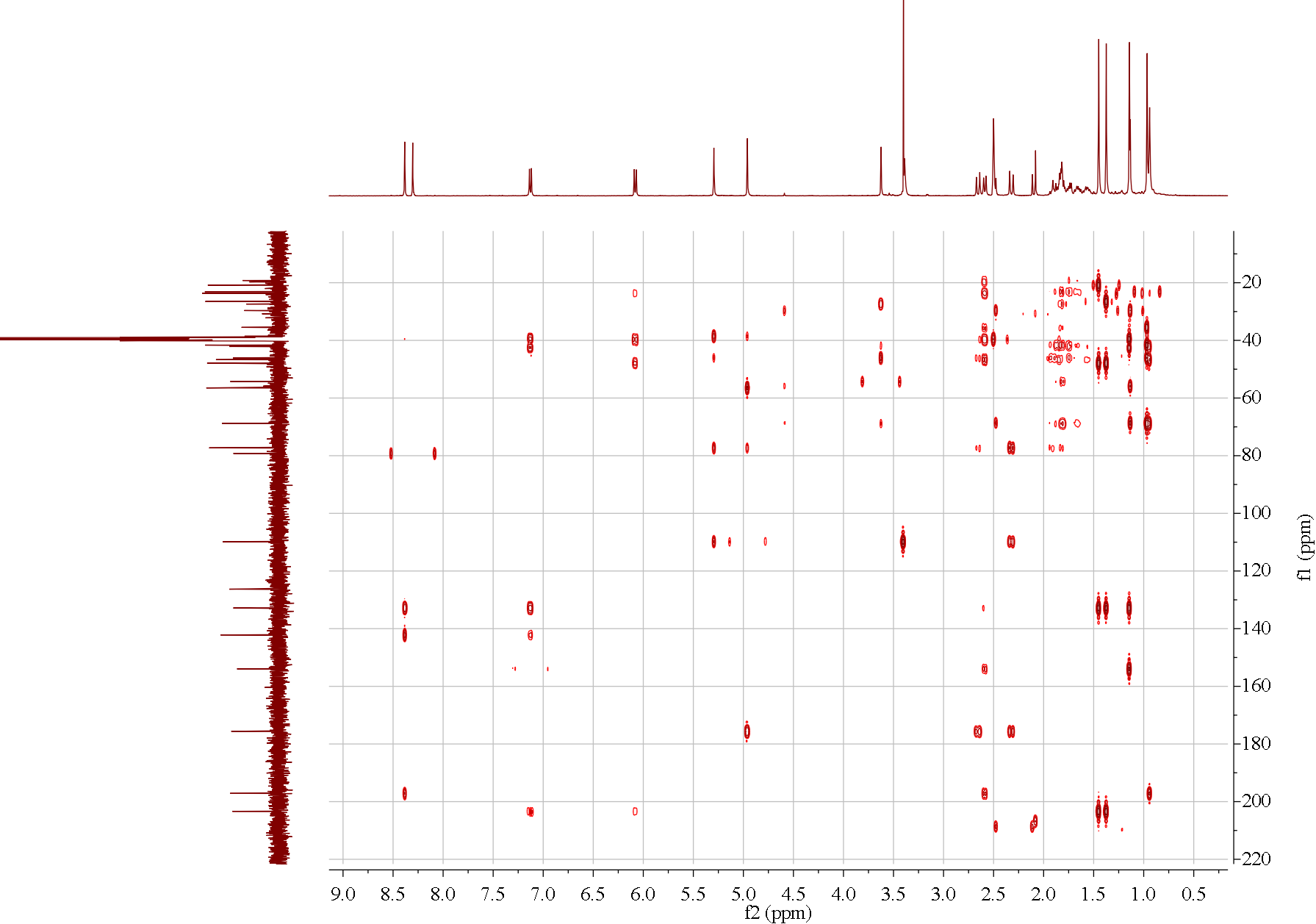


Figure S33. ROESY spectrum (500 MHz) of walsurobustone D (4) in (CD_3_)_2_SO


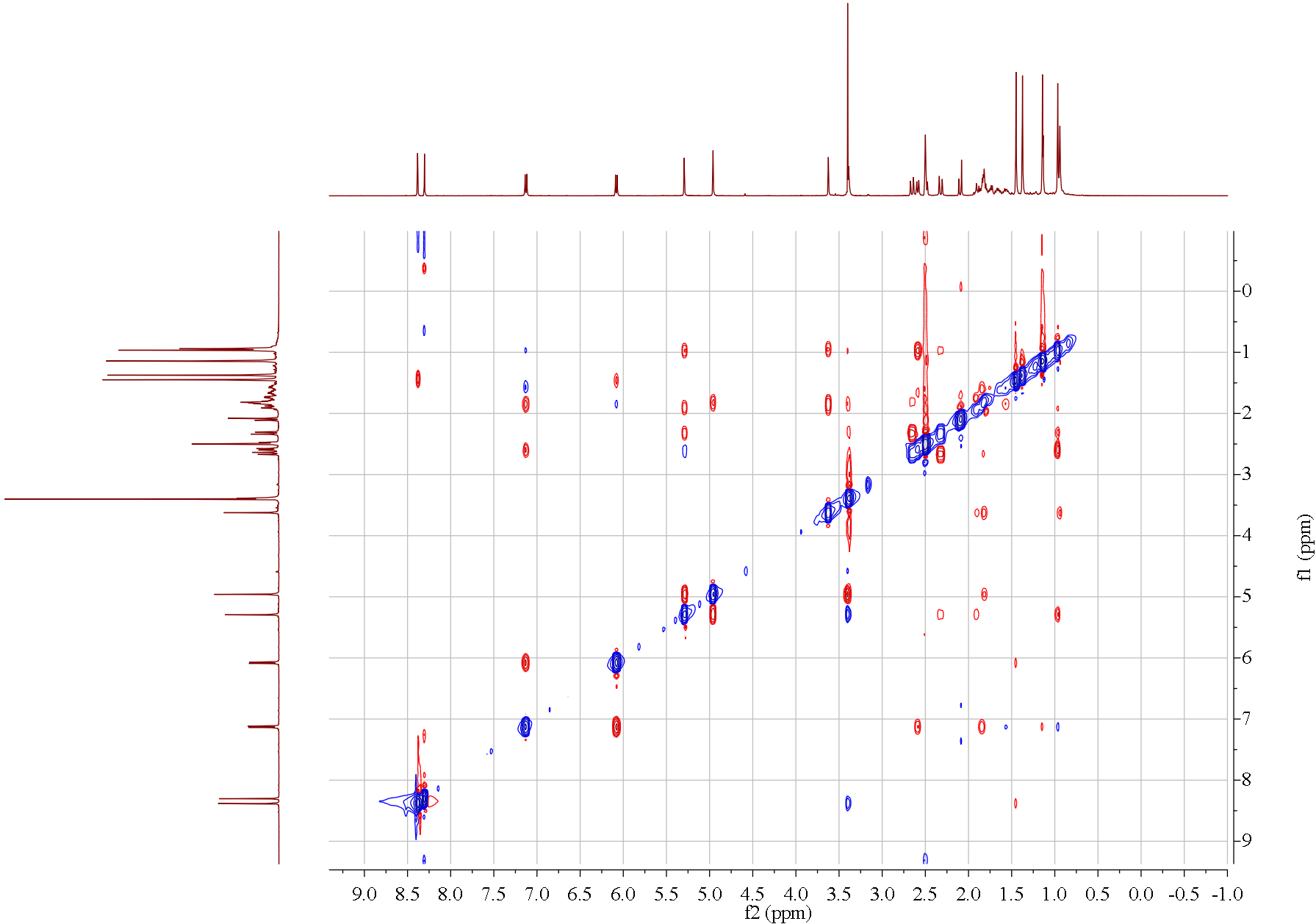


Figure S34. ESIMS spectrum of walsurobustone D (4)


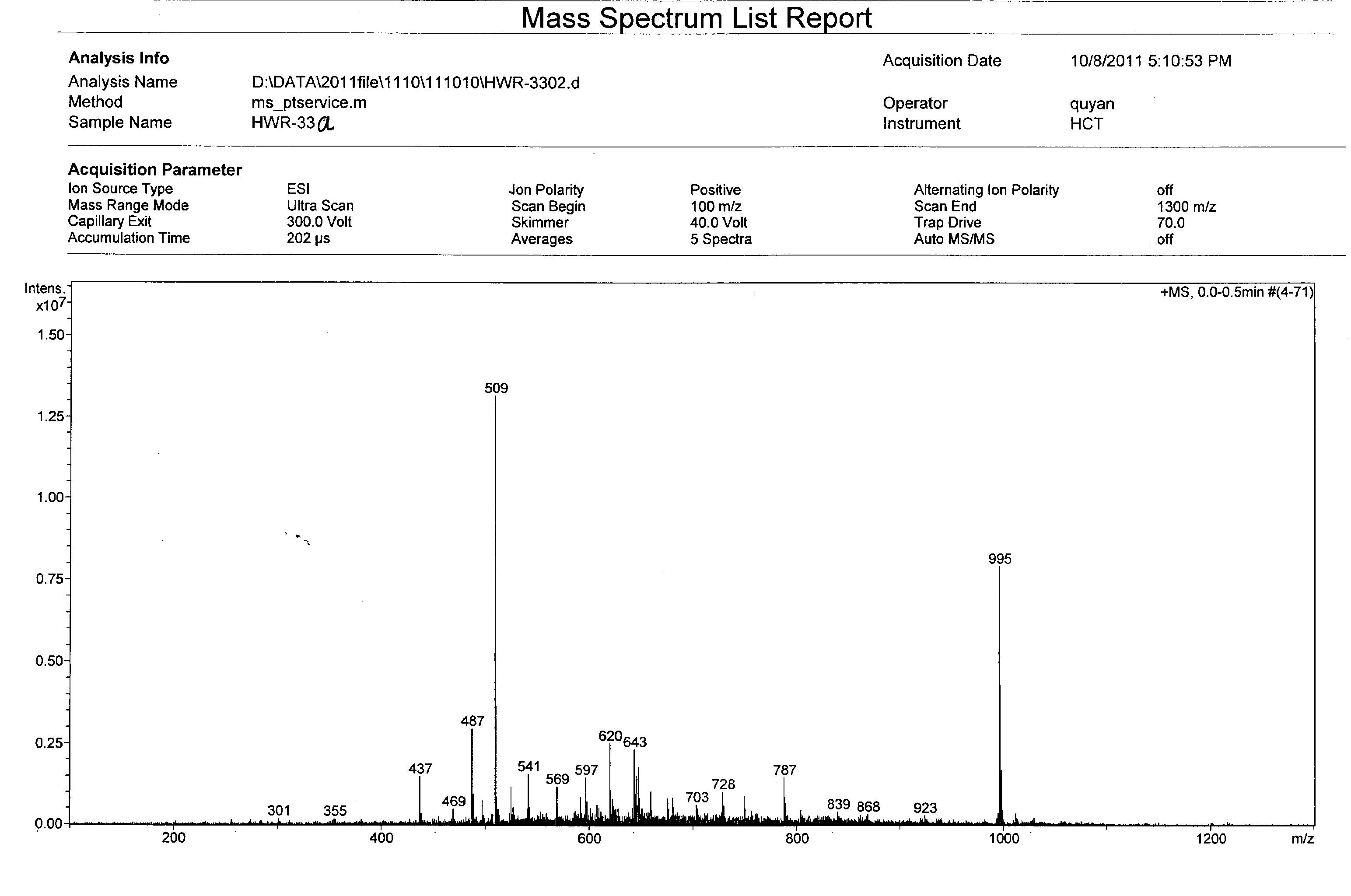


Figure S35. HRESIMS spectrum of walsurobustone D (4)


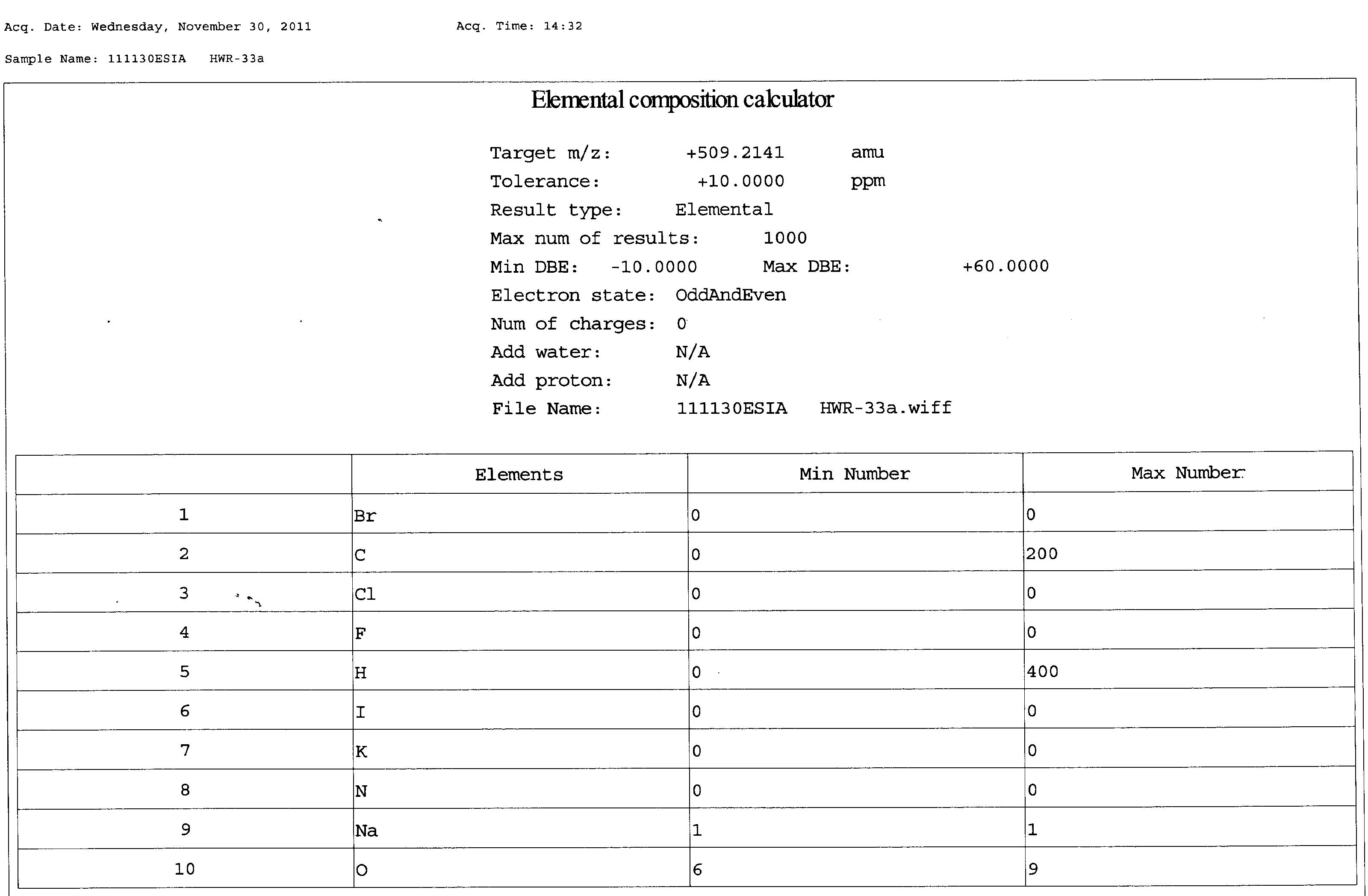

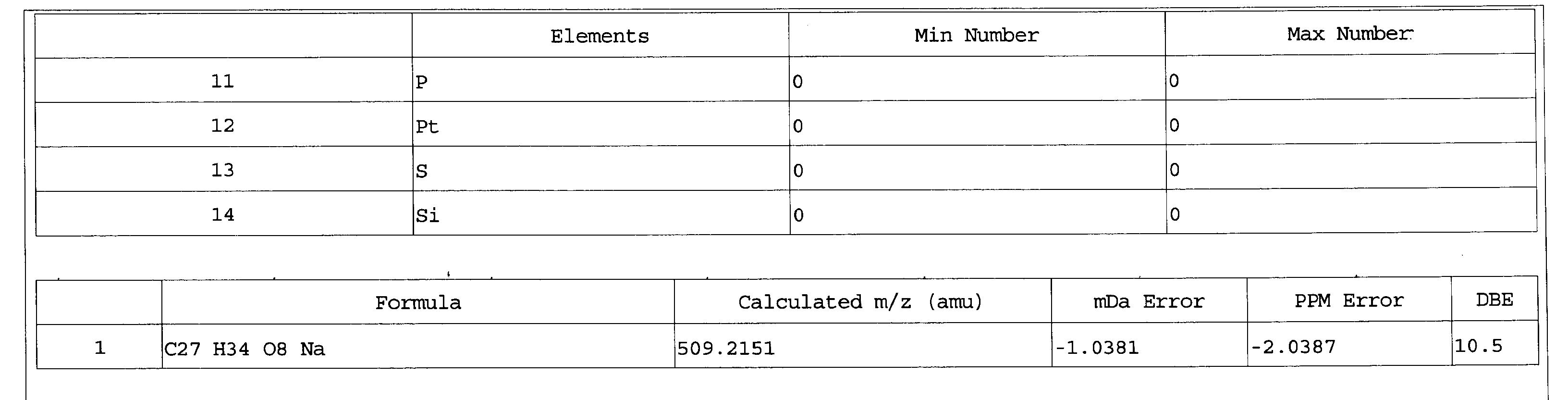


Figure S36. IR (KBr disc) spectrum of walsurobustone D (4)


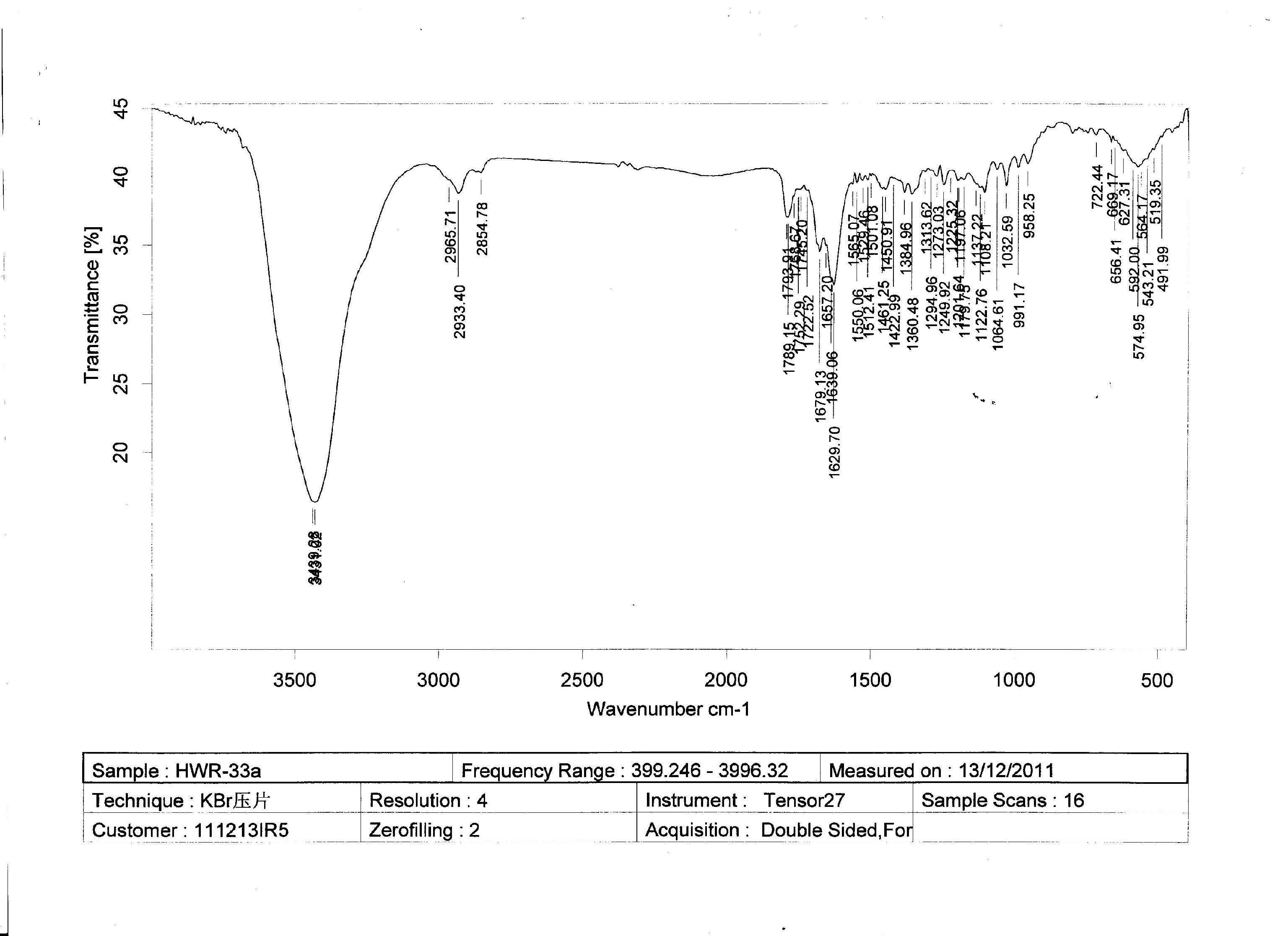


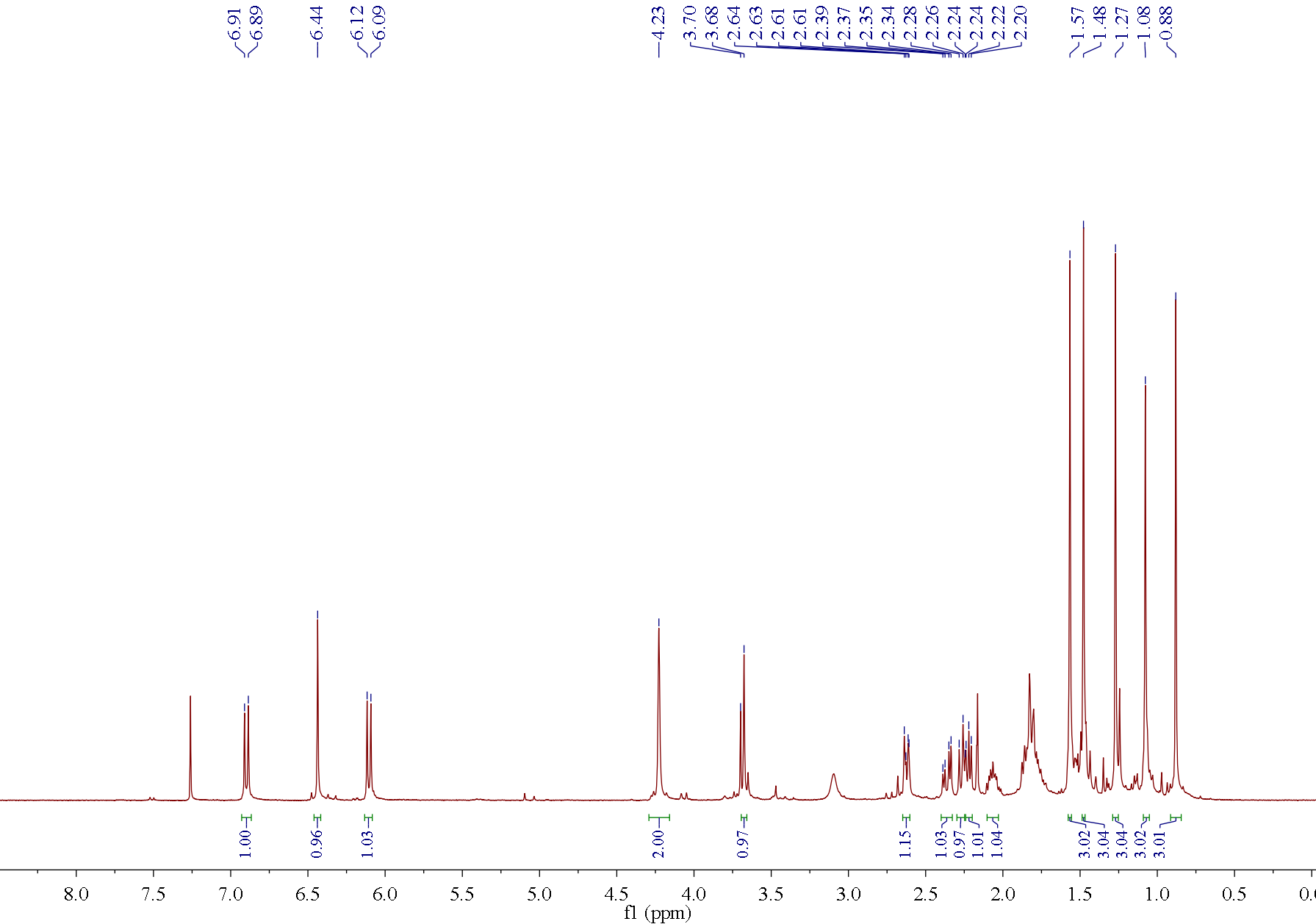
Figure S37. ^1^H NMR spectrum (400 MHz) of walsurobustone E (5) in CDCl_3_

Figure S38. ^13^CNMR spectrum (100 MHz) of walsurobustone E (5) in CDCl_3_


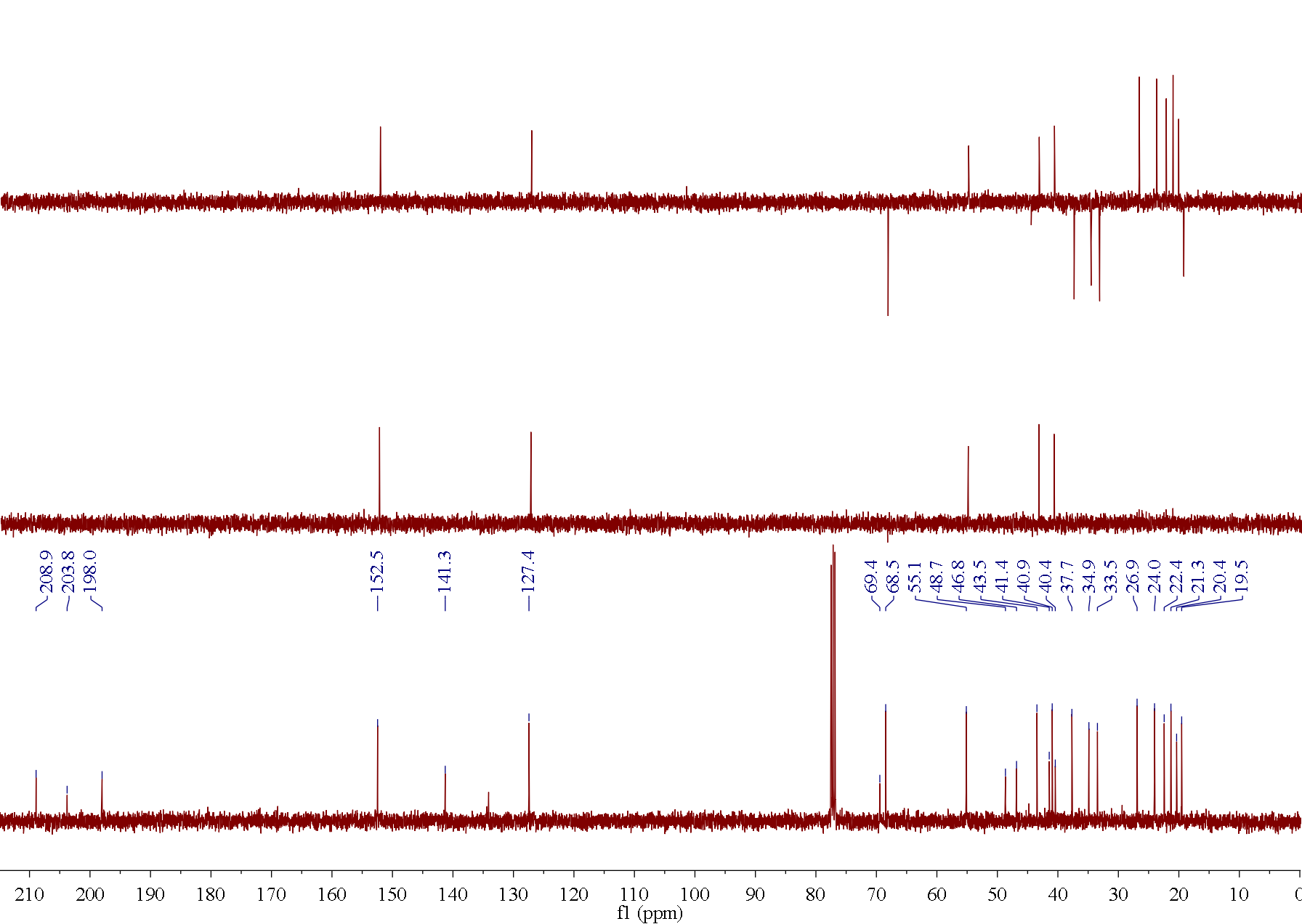


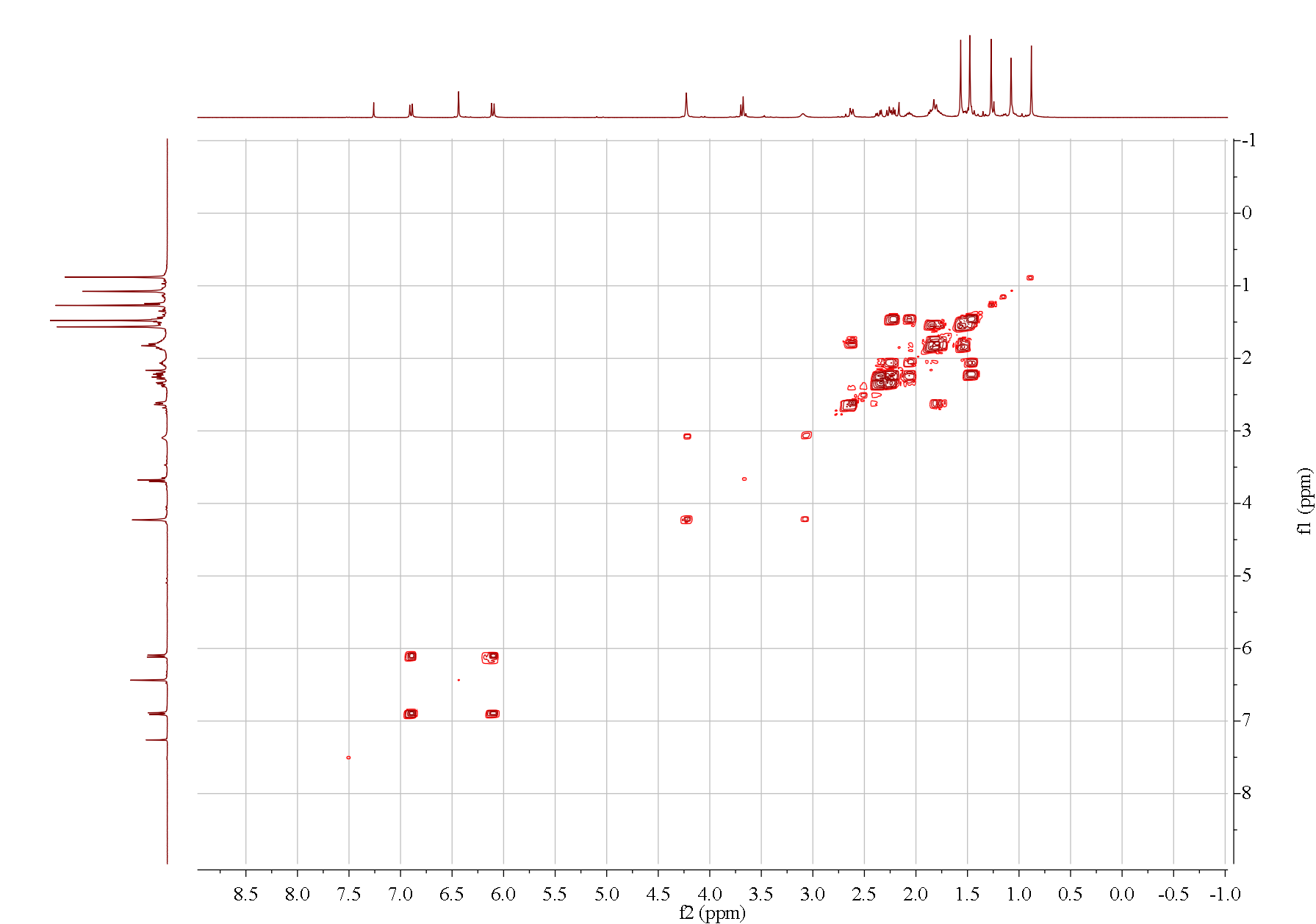
Figure S39. ^1^H-^1^H COSY spectrum (500 MHz) of walsurobustone E (5) in CDCl_3_


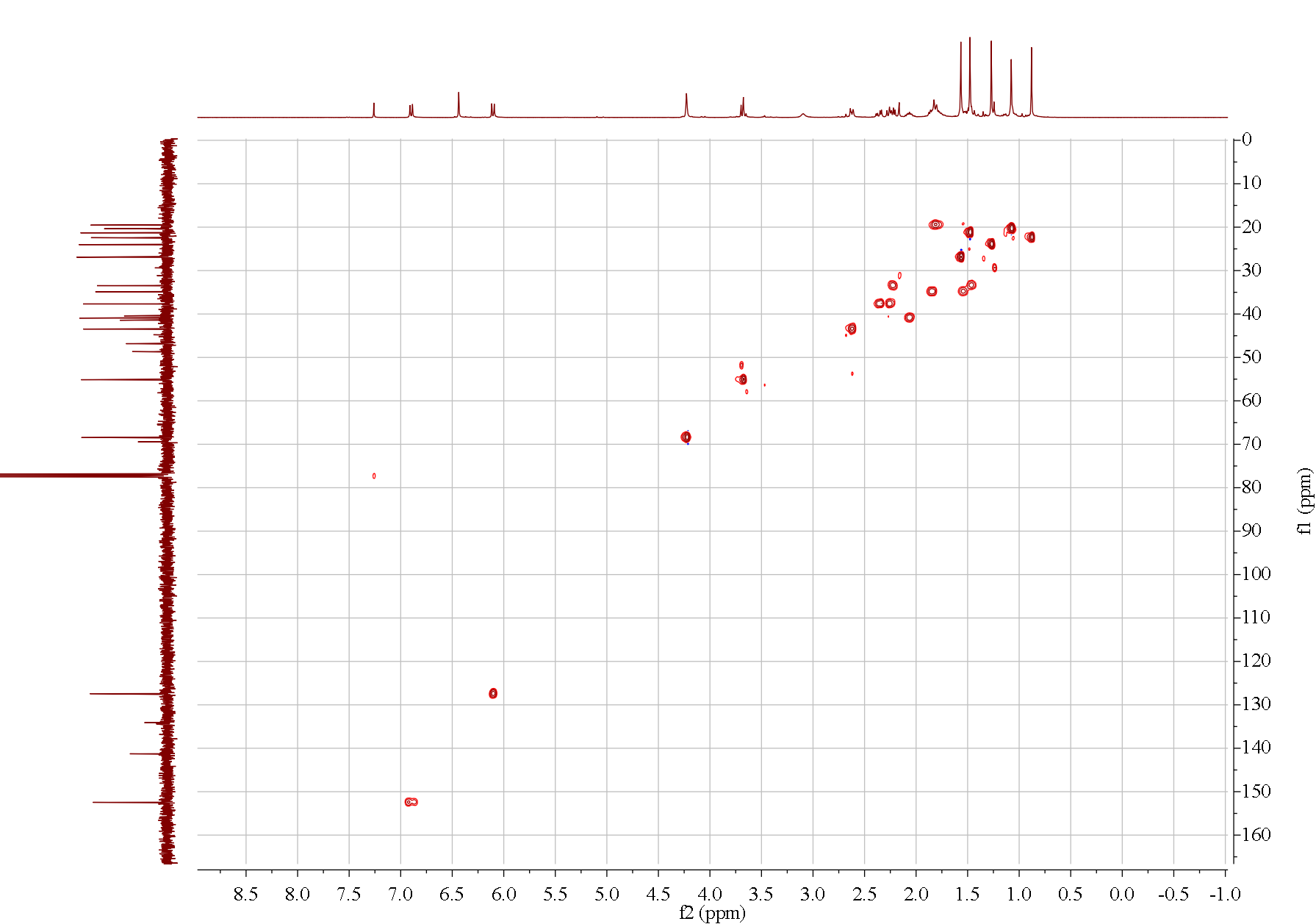
Figure S40. HSQC spectrum (500 MHz) of walsurobustone E (5) in CDCl_3_


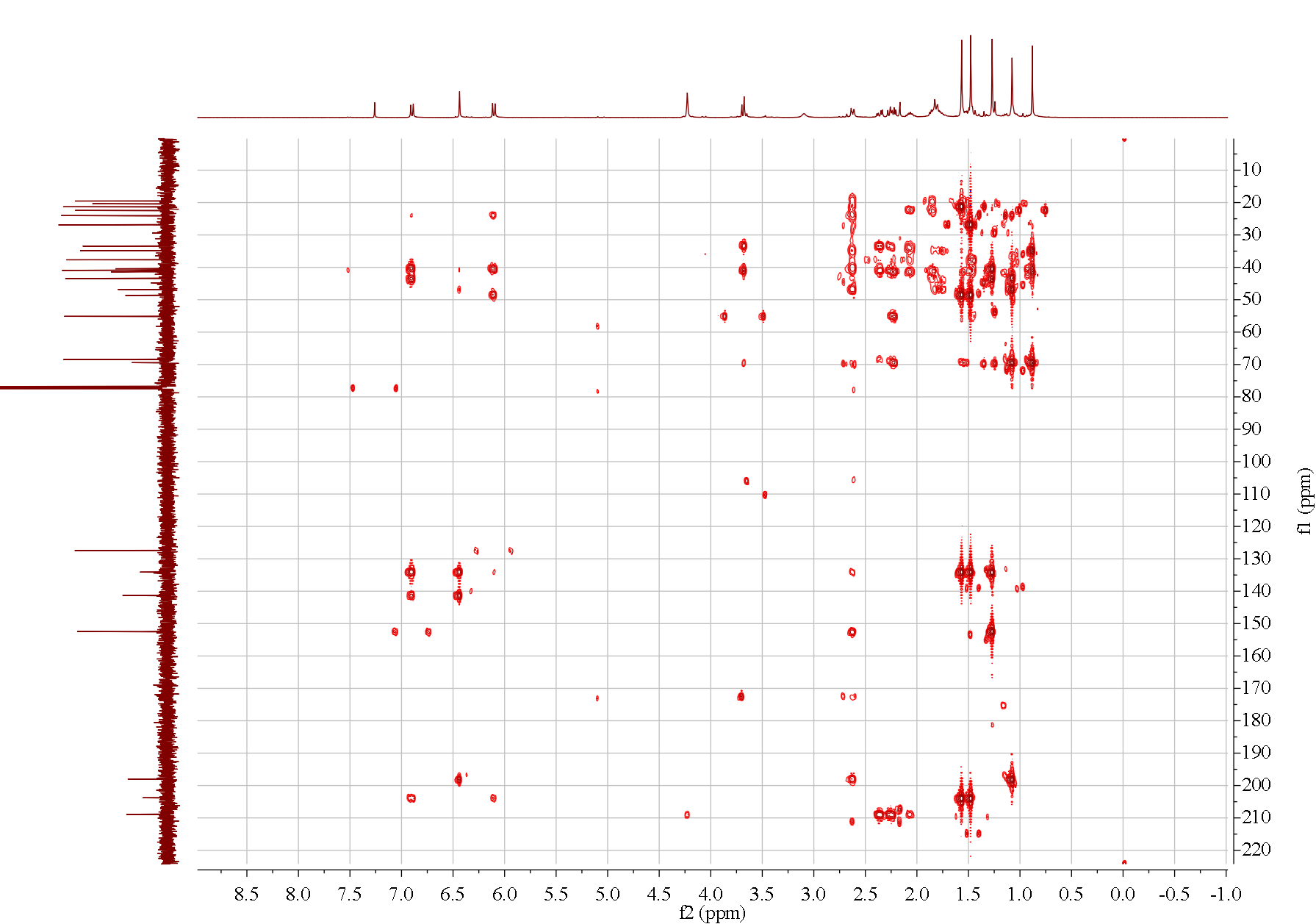
Figure S41. HMBC spectrum (500 MHz) of walsurobustone E (5) in CDCl_3_

Figure S42. ROESY spectrum (500 MHz) of walsurobustone E (5) in CDCl_3_


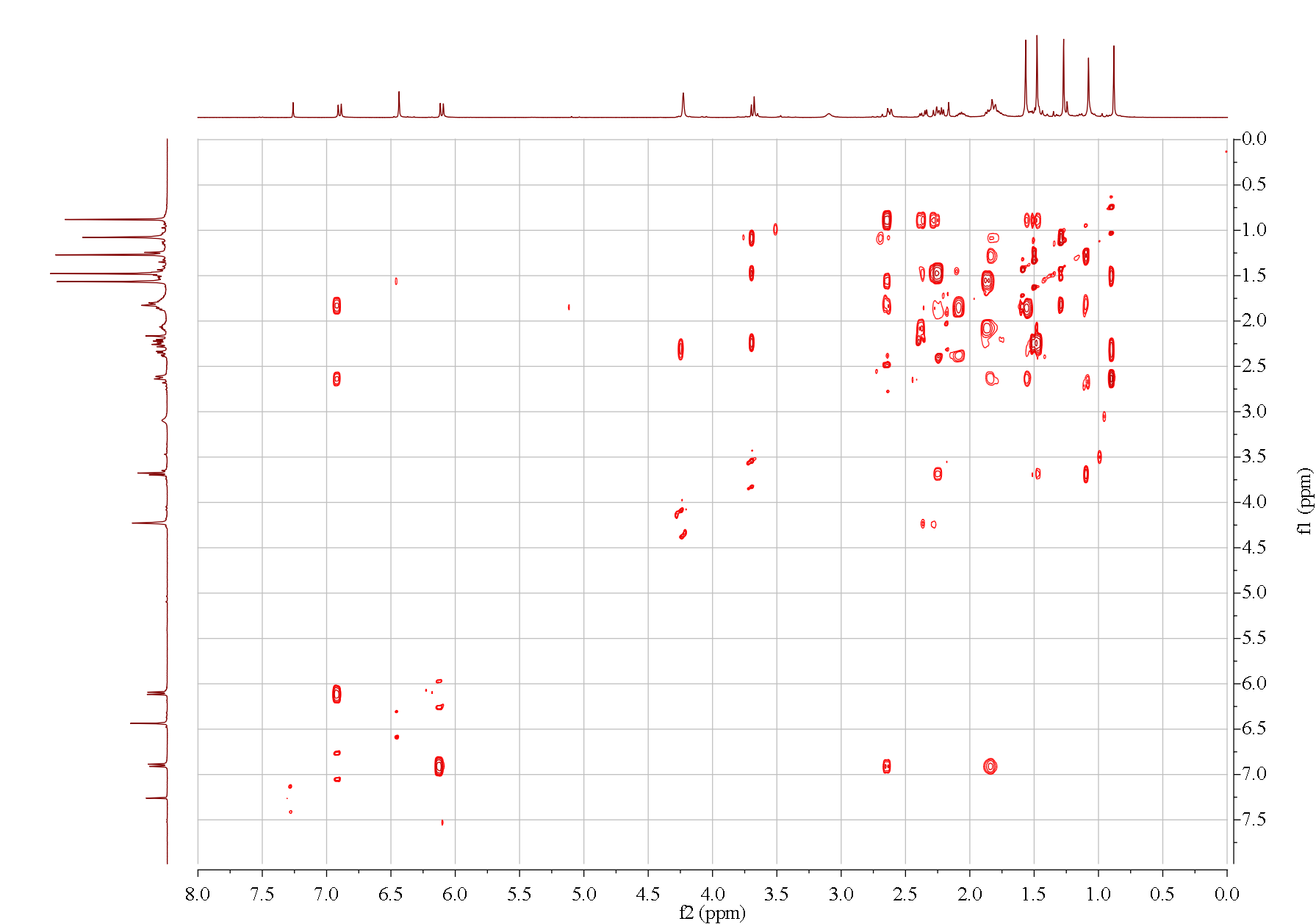


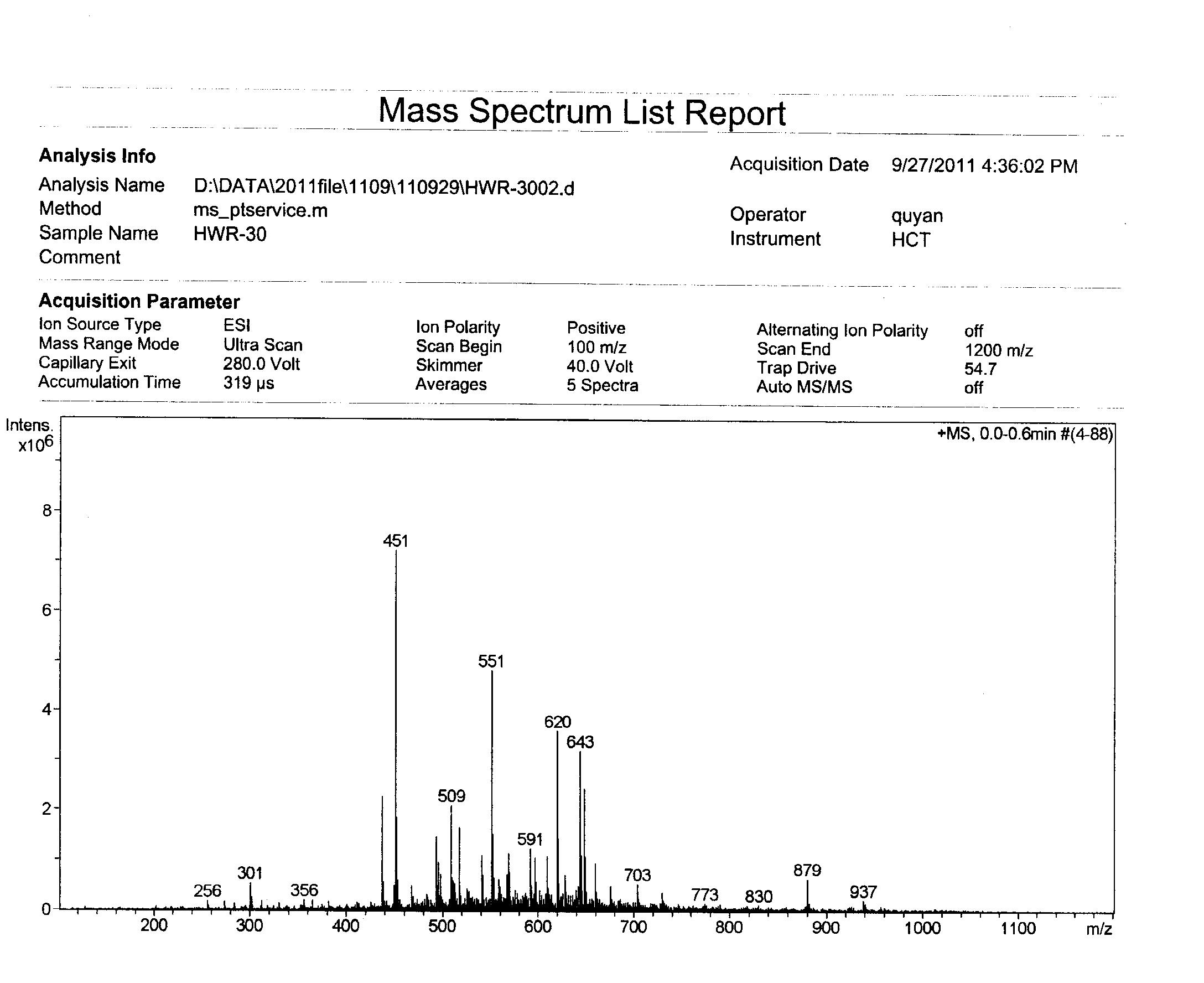
Figure S43. ESIMS spectrum of walsurobustone E (5)

Figure S44. HRESIMS spectrum of walsurobustone E (5)


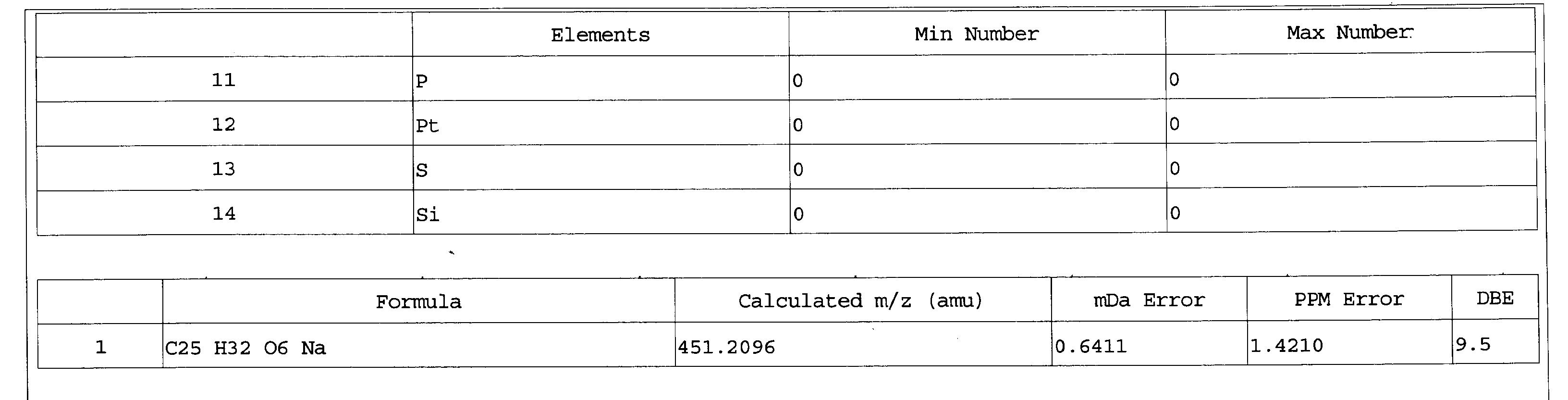

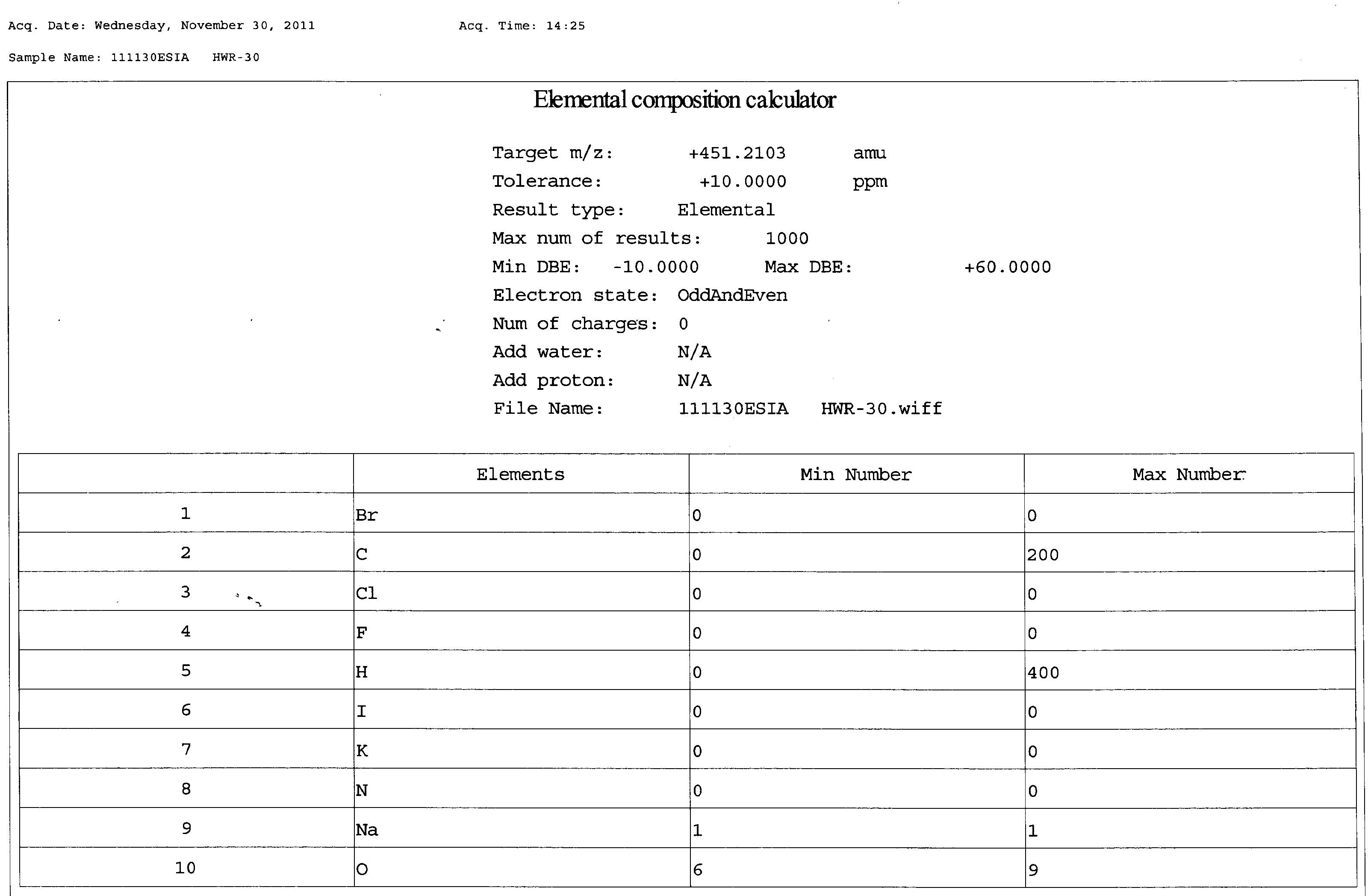


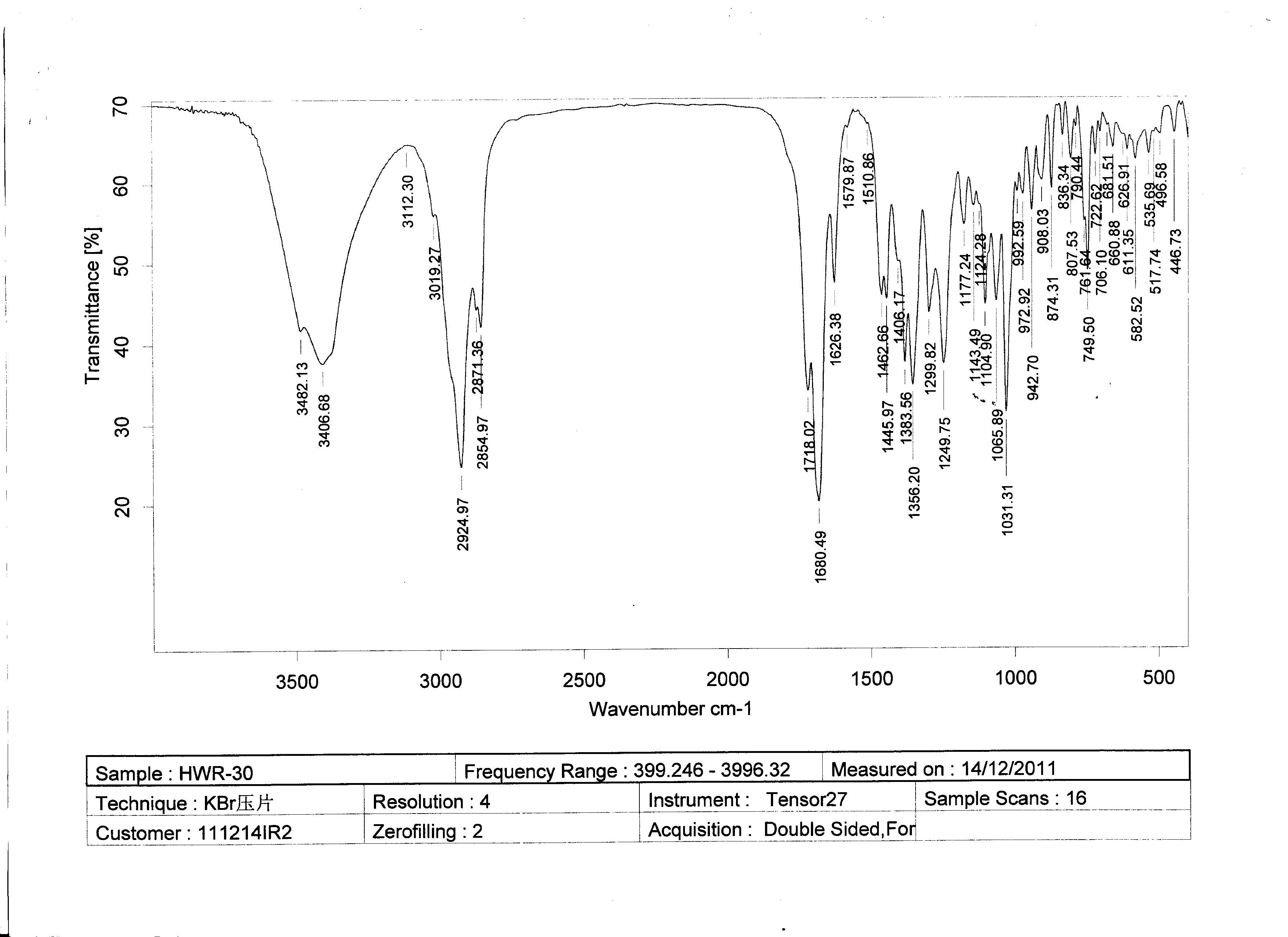
Figure S45. IR (KBr disc) spectrum of walsurobustone E (5)


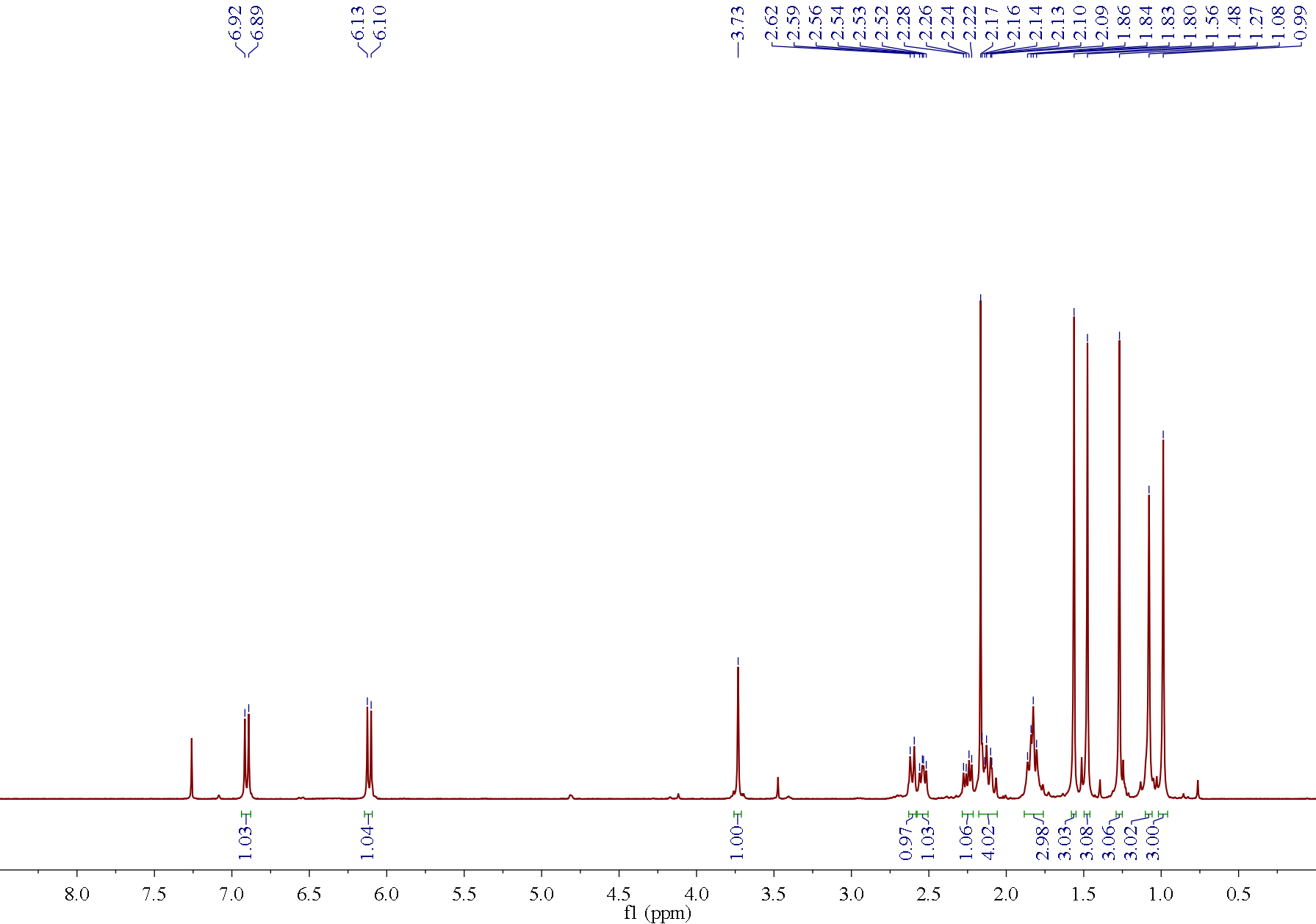


Figure S46.^1^H NMR spectrum (400 MHz) of toonapubesic acid B (6) in CDCl_3_

Figure S47.^13^CNMR spectrum (100 MHz) of toonapubesic acid B (6) in CDCl_3_


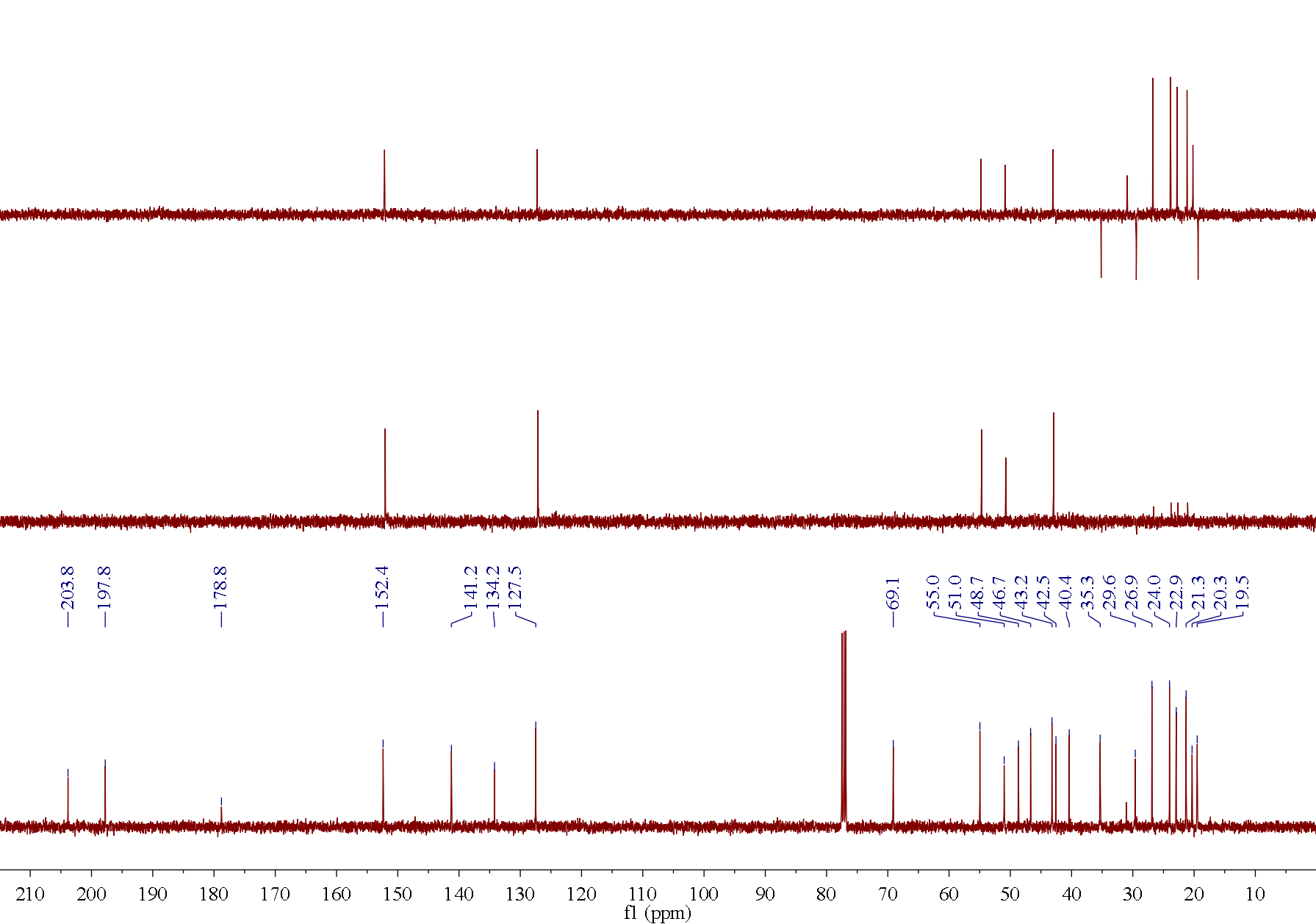


Figure S48. ^1^H-^1^H COSY spectrum (500 MHz) of toonapubesic acid B (6) in CDCl_3_


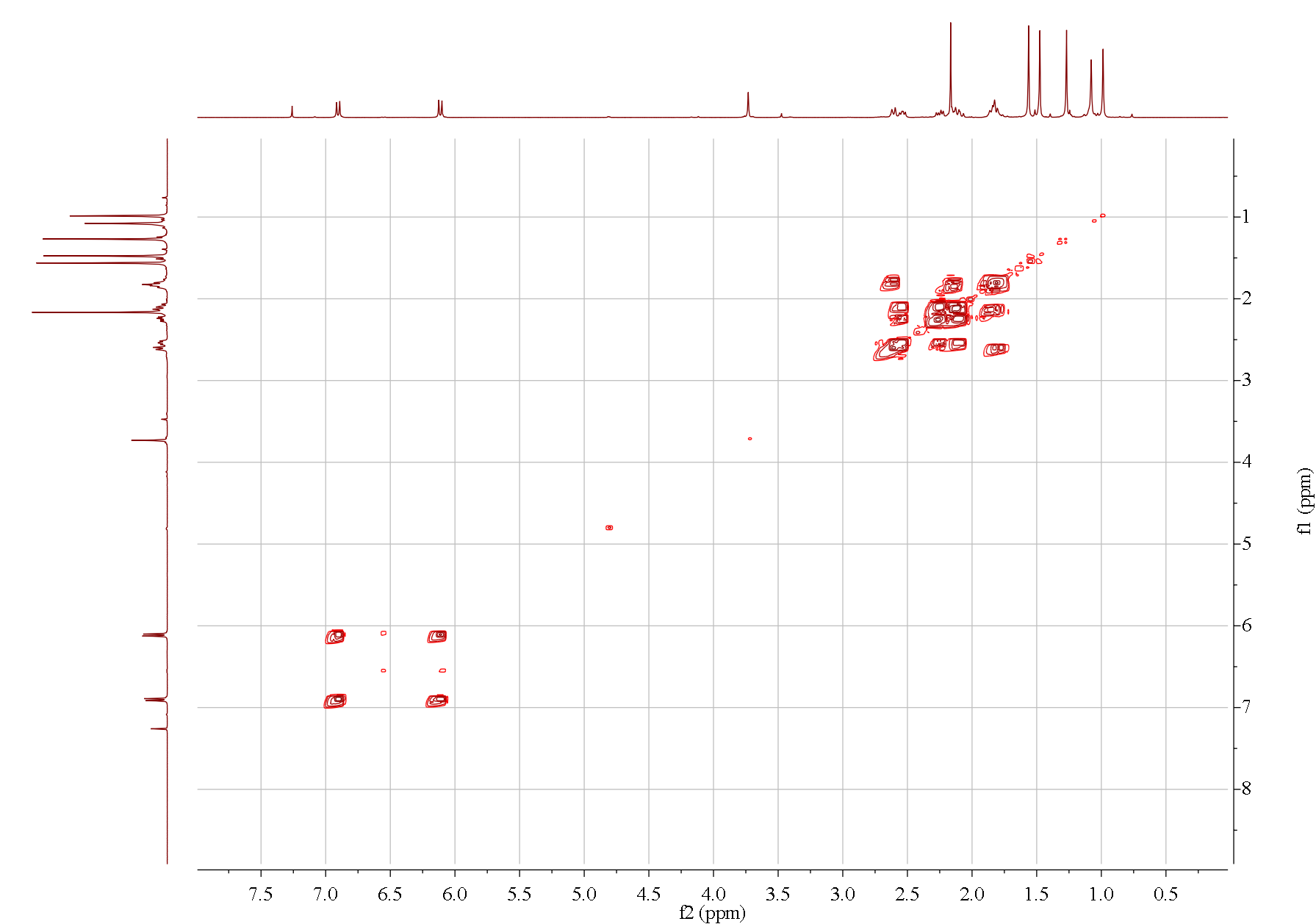


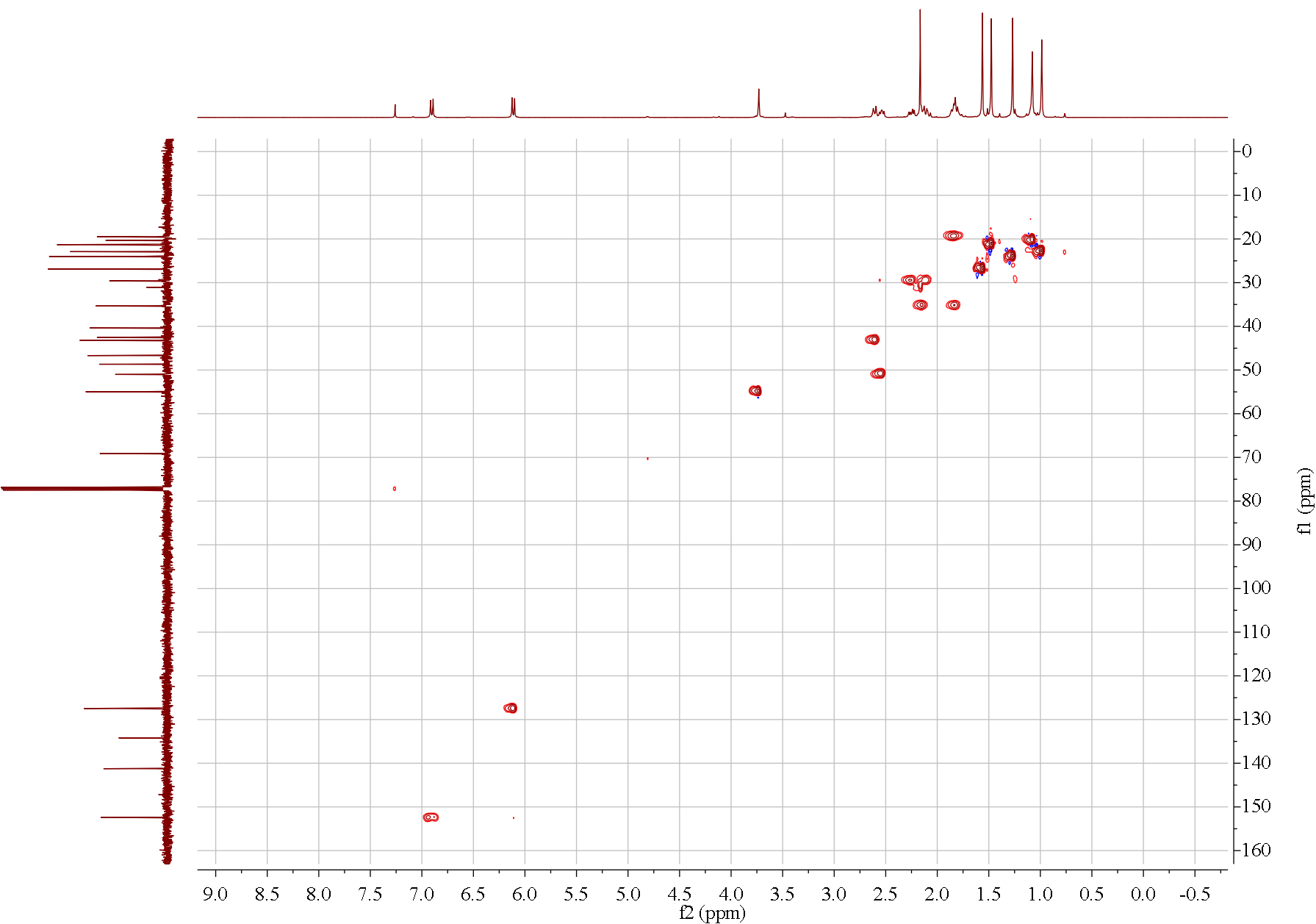
Figure S49. HSQC spectrum (500 MHz) of toonapubesic acid B (6) in CDCl_3_

Figure S50. HMBC spectrum (500 MHz) of toonapubesic acid B (6) in CDCl_3_


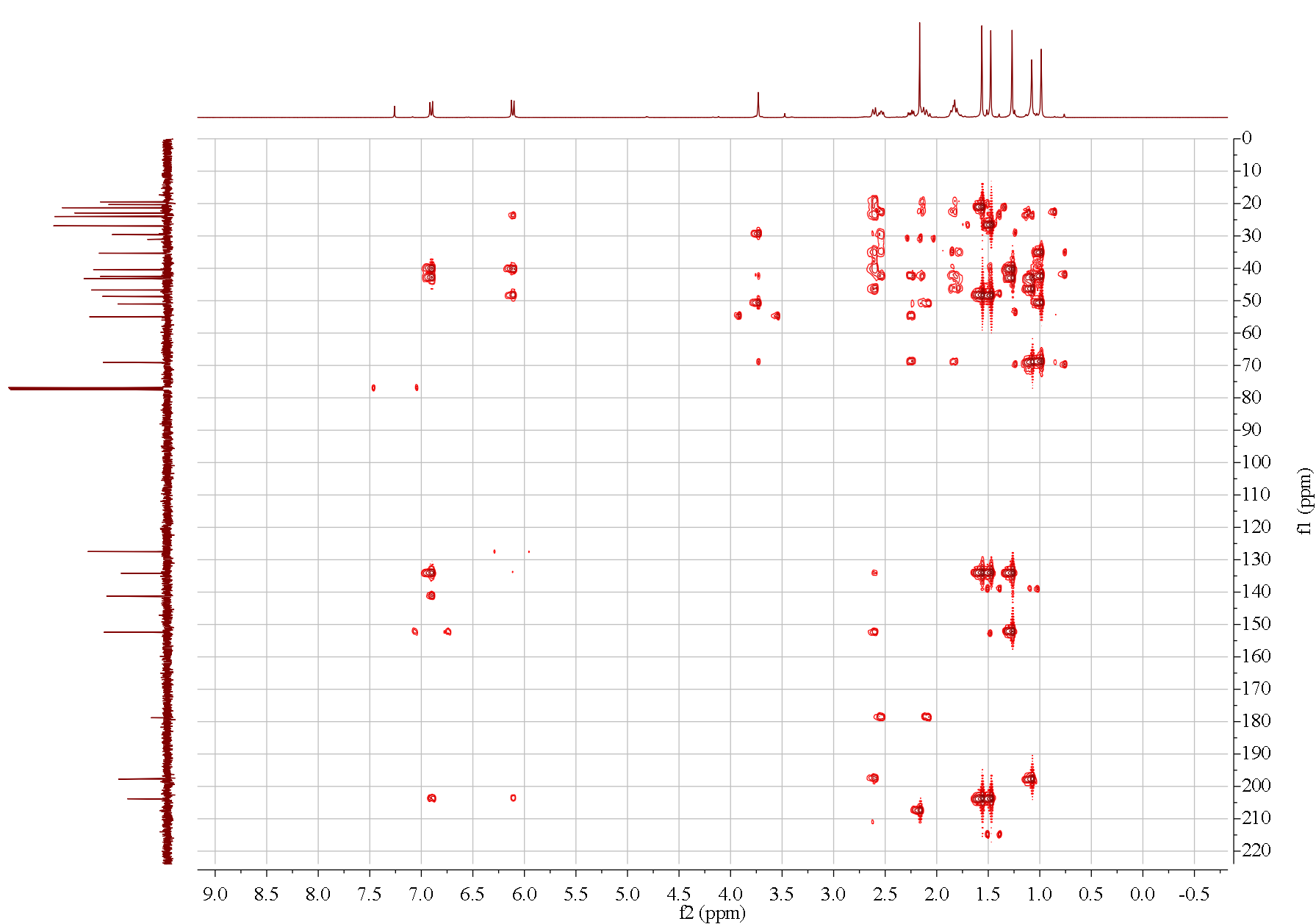


Figure S51. ROESY spectrum (500 MHz) of toonapubesic acid B (6) in CDCl_3_


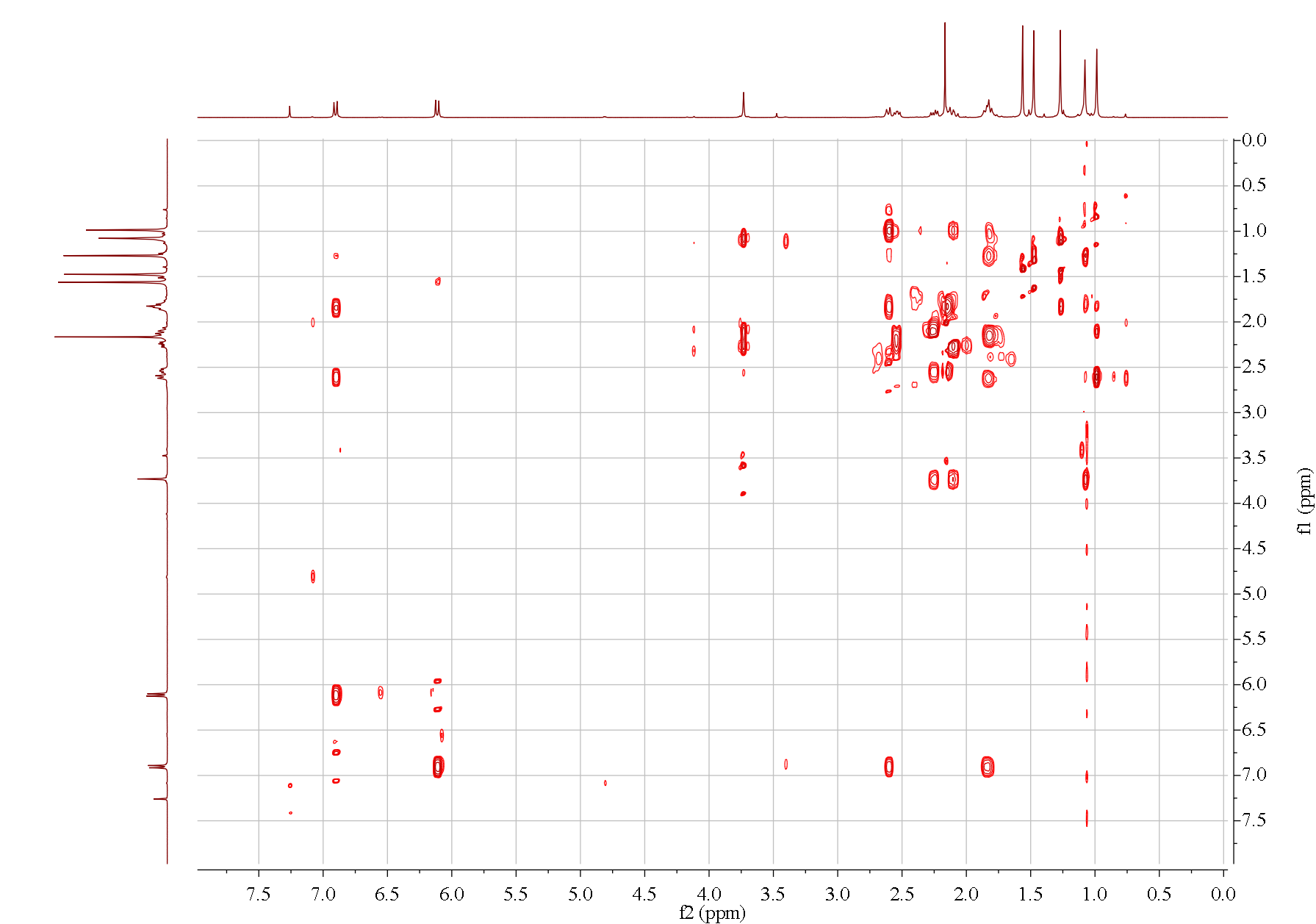


Figure S52. ESIMS spectrum of toonapubesic acid B (6)


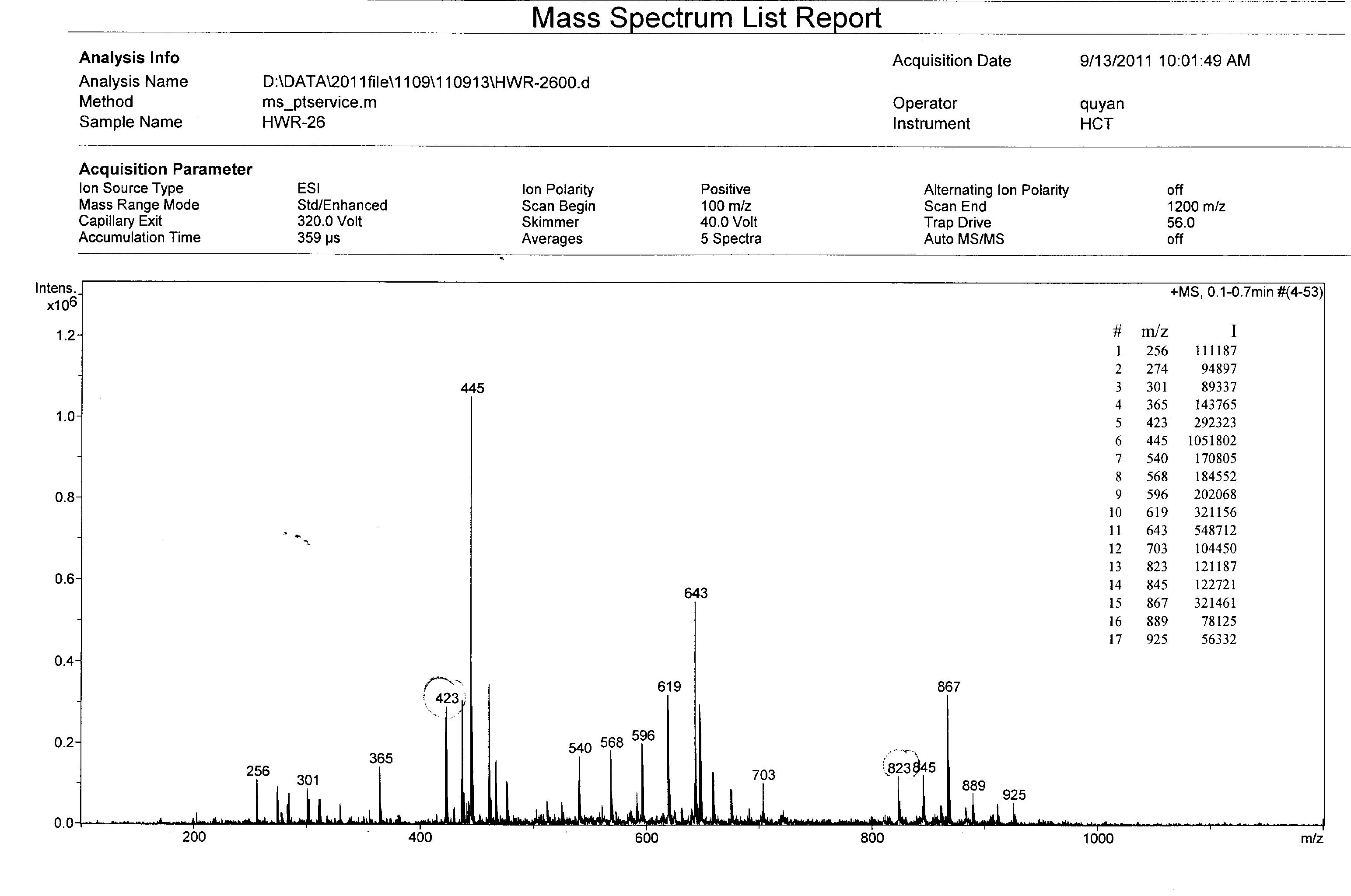


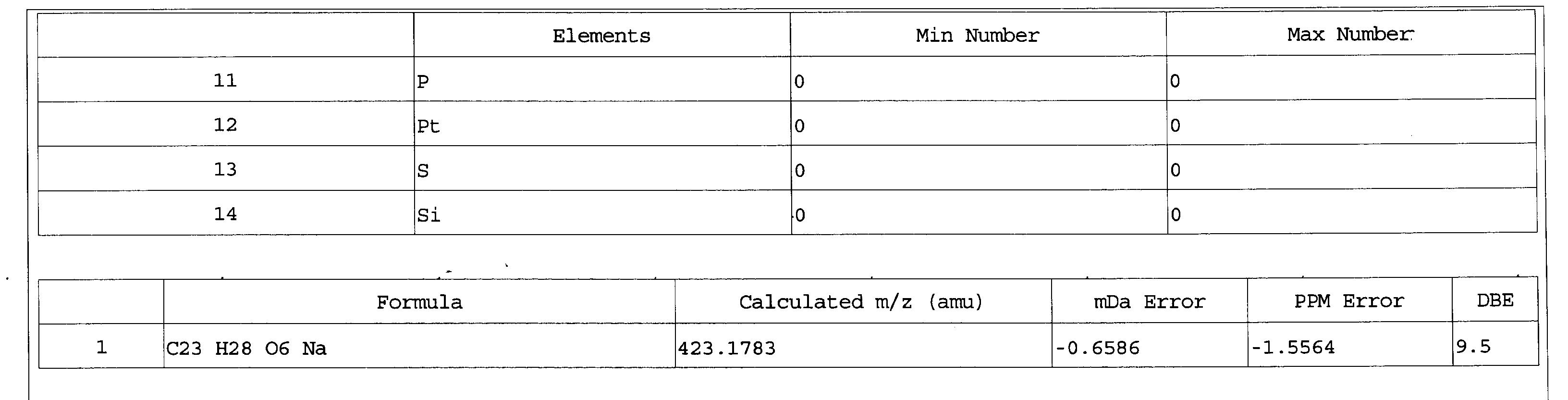

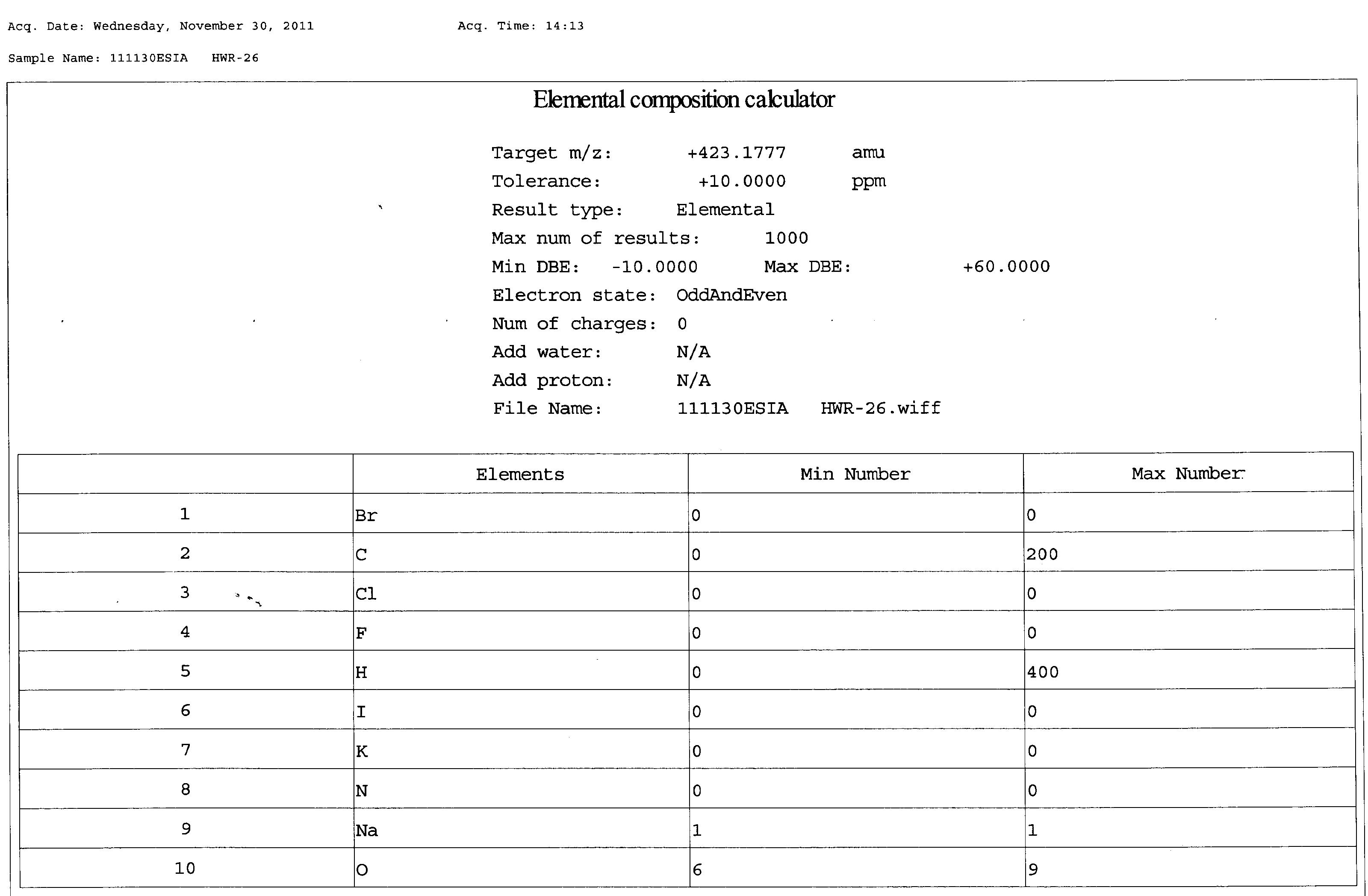

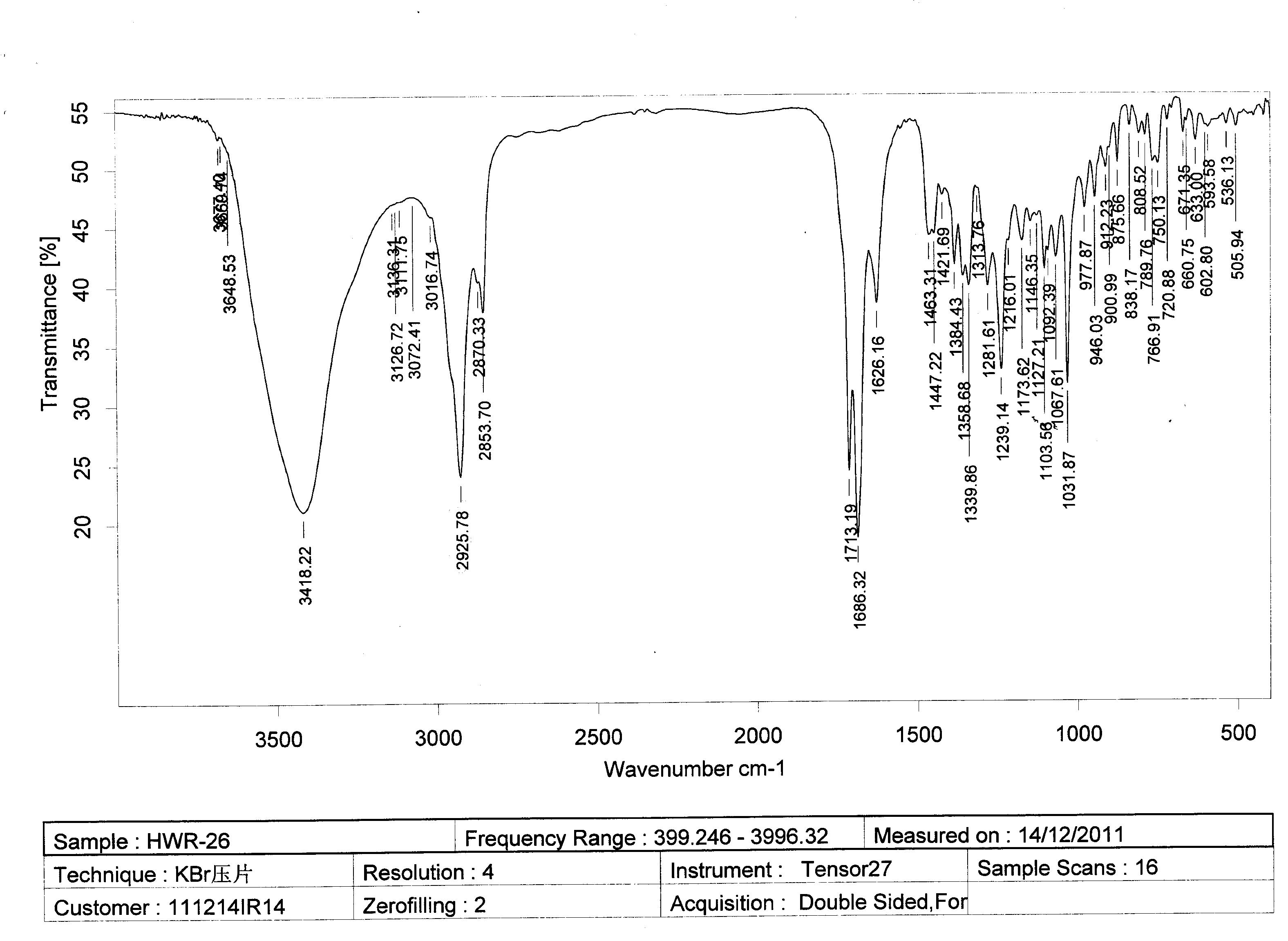
Figure S53. HRESIMS spectrum of toonapubesic acid B (6)

Figure S54. IR (KBr disc) spectrum of toonapubesic acid B (6)


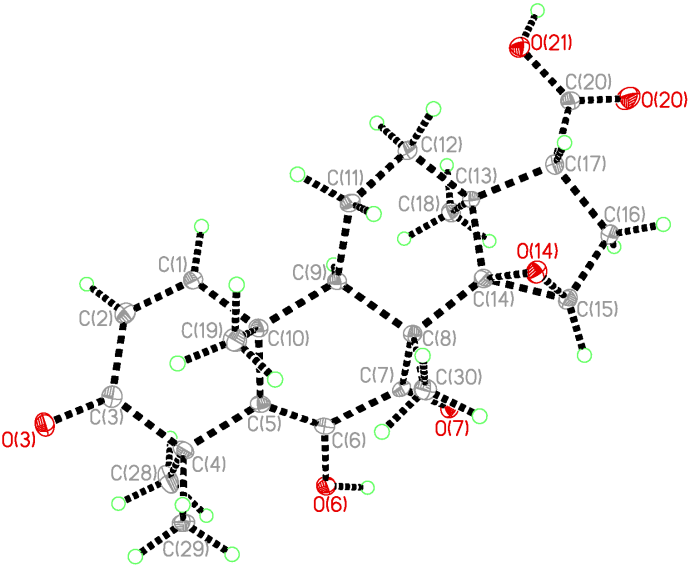
Figure S55. Single-crystal X-ray structure of toonapubesic acid B (6)
